# Secondary prevention of antithrombotic therapy in patients with stable cardiovascular disease at high ischemic risk: A network meta-analysis of randomized controlled trials

**DOI:** 10.3389/fcvm.2022.1040473

**Published:** 2023-01-09

**Authors:** Houyong Zhu, Xiaoqun Xu, Hanxin Wang, Qilan Chen, Xiaojiang Fang, Jianwu Zheng, Beibei Gao, Guoxin Tong, Liang Zhou, Tielong Chen, Jinyu Huang

**Affiliations:** ^1^Department of Cardiology, Hangzhou TCM Hospital Affiliated to Zhejiang Chinese Medical University, Hangzhou, Zhejiang, China; ^2^Affiliated Hangzhou Chest Hospital, Zhejiang University School of Medicine, Hangzhou, Zhejiang, China; ^3^The Fourth School of Clinical Medicine, Zhejiang Chinese Medical University, Hangzhou, Zhejiang, China; ^4^Department of Cardiology, The Affiliated Hangzhou First People’s Hospital, Zhejiang University School of Medicine, Hangzhou, Zhejiang, China

**Keywords:** stable cardiovascular disease, coronary artery disease, peripheral artery disease, secondary prevention, antithrombotic therapy

## Abstract

**Aims:**

Antithrombotic secondary prevention in stable cardiovascular disease (SCVD) patients at high ischemic risk remains unclear. We compared the efficacy and safety of aspirin monotherapy, clopidogrel monotherapy, ticagrelor monotherapy, rivaroxaban monotherapy, clopidogrel plus aspirin, ticagrelor plus aspirin, and rivaroxaban plus aspirin in the high-risk ischemic cohorts.

**Methods and results:**

Eleven randomized controlled trials were included (*n* = 111737). The primary outcomes were major cardiovascular and cerebrovascular events (MACEs) and major bleeding. A random effects model was used for frequentist network meta-analysis. Odds ratio (OR) and 95% credible intervals (CI) were reported as a summary statistic. Compared with aspirin monotherapy, rivaroxaban plus aspirin [OR 0.79 (95% CI, 0.69, 0.89)], ticagrelor plus aspirin [0.88 (0.80, 0.98)], clopidogrel plus aspirin [0.56 (0.41, 0.77)] were associated with a reduced risk of MACEs, but rivaroxaban monotherapy [0.92 (0.79, 1.07)], ticagrelor monotherapy [0.68 (0.45, 1.05)], and clopidogrel monotherapy [0.67 (0.43, 1.05)] showed no statistically significant difference. However, rivaroxaban monotherapy and all dual antithrombotic strategies increased the risk of major bleeding to varying degrees, with ticagrelor plus aspirin associated with the highest risk of major bleeding. The net clinical benefit favored clopidogrel or ticagrelor monotherapy, which have a mild anti-ischemic effect without an increase in bleeding risk.

**Conclusion:**

The present network meta-analysis suggests that clopidogrel or ticagrelor monotherapy may be recommended first in this cohort of SCVD at high ischemic risk. But clopidogrel plus aspirin or rivaroxaban plus aspirin can still be considered for use in patients with recurrent MACEs.

## Introduction

Cardiovascular disease is the leading cause of death worldwide (1). Coronary artery disease (CAD), peripheral artery disease (PAD), and stroke are potent predictors of cardiovascular events (1, 2). Antithrombotic therapy plays an important role in preventing the pathogenesis of atherothrombosis. The antithrombotic strategy for long-term secondary prevention in patients with stable cardiovascular disease (SCAD) is still uncertain. Low-dose aspirin is found to reduce ischemic outcomes in patients over a certain risk threshold (3), but aspirin monotherapy is insufficient for preventing ischemic events in high-risk patients (4).

Recently, the COMPASS [Cardiovascular Outcomes for People Using Anticoagulation Strategies] trial (5) demonstrated that a regimen of rivaroxaban plus aspirin had advantages over a regimen of aspirin alone in secondary prevention with SCAD at high ischemic risk, and this benefit was equally present in the CAD and PAD subgroups in the COMPASS trial (6, 7).

Our previous network meta-analysis (NMA) (8) explored the best strategy for long-term antithrombotic therapy in a broad chronic coronary syndrome (CCS) population. It showed that the combined benefits of rivaroxaban plus aspirin were better than aspirin alone, rivaroxaban alone, and ticagrelor plus aspirin. However, due to the limited high-quality trials meeting the inclusion criteria, fewer exploratory antithrombotic regimens were included in the network analysis structure.

Although arteriosclerotic cardiovascular disease (ASCVD) includes CAD, PAD, and stroke, exploratory dual antiplatelet therapy achieved no significant clinical benefit over aspirin alone for this population in the CHARISMA [Clopidogrel for High Atherothrombotic Risk and Ischemic Stabilization, Management, and Avoidance] trial (9). Several recent studies (10, 11) of antithrombotic therapy in stroke also confirmed the prominent clinical heterogeneity of stroke compared with CAD and PAD, which may be partly explained by hemorrhagic transformation after cerebral infarction. We hypothesized that if this network meta-analysis examined a “COMPASS-like” high-risk secondary prevention population, rivaroxaban plus aspirin or other exploratory antithrombotic regimens would have more net clinical benefits than aspirin monotherapy.

## Methods

The Preferred Reporting Items for Systematic Reviews and Meta Analyses (PRISMA) guidelines (Supplementary Table 1) were used in this systematic review and network meta-analysis (12). As this study is a meta-analysis, all data supporting the findings of this study are available in the included original studies, and data extracted by the authors are also available from the corresponding author upon reasonable request.

### Data sources

The Medline, EMBASE, and Cochrane database were independently searched by two reviewers. The full list of search terms is provided in the Supplementary Table 2. Searches for studies published up to March 2021 were reasonably screened.

### Study selection, data extraction, and quality assessment

An initial eligibility screen of all retrieved titles and abstracts was conducted, and original studies were included in our network meta-analysis if they met the following criteria: (1) randomized controlled trial (RCT) with two or more arms; (2) the inclusion criteria of “COMPASS-like,” including 1) myocardial infarction within 20 years, either multi-vessel CAD, or previous multi-vessel percutaneous coronary intervention (PCI), or previous multi-vessel coronary artery bypass grafting (CABG) surgery; 2) Previous limb revascularization, or previous lower extremity amputation, or PAD with history of intermittent claudication and an ankle/arm blood pressure (BP) ratio < 0.90, or previous carotid revascularization or asymptomatic carotid artery stenosis ≥ 50%; (3) anticoagulant and/or antiplatelet therapy; (4) reported major cardiovascular and cerebrovascular events (MACEs) and major bleeding accompanied by follow-up events for more than 12 months. The exclusion criteria were as follows: (1) Patients originally planned to receive dual antiplatelet therapy or anticoagulant therapy; (2) In addition to intervention drugs, there are other antithrombotic drugs in studies, which are not suitable for the structure of network meta-analysis. The methods of data extraction were outlined in our previous study (8). Two reviewers used the seven domains of the Cochrane risk of bias tool (13) to evaluate the quality of the included studies according to our previous study (8).

### Outcome measures

The primary efficacy outcome was trial-defined MACEs, which was often defined as a combination of death from any cause or cardiovascular death, myocardial infarction (MI), and stroke. The primary safety outcome was major bleeding as defined in the respective trials, usually based on Thrombolysis in Myocardial Infarction (TIMI)-defined major bleeding (14), International Society on Thrombosis and Haemostasis (ISTH)-defined major bleeding (15), Global Utilization of Streptokinase and Tissue Plasminogen Activator for Occluded Arteries (GUSTO)-defined severe bleeding (16), or Bleeding Academic Research Consortium (BARC)-defined type 3 or 5 bleeding (17). Secondary efficacy outcomes were components of these MACEs. Secondary safety outcomes were minor bleeding and intracranial hemorrhage. The net clinical benefit was assessed by combining the results of major efficacy and safety outcomes.

### Subgroup analysis

The primary efficacy outcome, primary safety outcome, all-cause death, and cardiovascular death were analyzed for the population with CAD and PAD. The inclusion criteria for the patients in the subgroup with PAD were as above.

### Statistical analysis

A standard paired meta-analysis was performed using a DerSimonian-Laird random effects model. The odds ratios (ORs) (95% credible intervals (CIs)) served as a summary statistic. Statistically significant results were those results where the 95% CI did not include 1. The heterogeneity test was completed using the χ^2^-based Q-test, and a *P* value <0.1 was considered to indicate heterogeneous results, whereas a *P* value >0.1 was considered to indicate a lack of heterogeneity. If heterogeneity was observed in the results, the degree of heterogeneity was determined using the *I*^2^ test (*I*^2^ = 0–25%, no heterogeneity; *I*^2^ = 25–50%, moderate heterogeneity; *I*^2^ = 50–75%, substantial heterogeneity; and *I*^2^ = 75–100%, extreme heterogeneity).

A frequentist network meta-analysis was performed with a restricted estimation maximum likelihood random effects model. The OR (95% CI) served as a summary statistic. We calculated the surface under the cumulative ranking (SUCRA) value to evaluate the rankings of treatment strategies. SUCRA values are presented as the percentage of the area under the cumulative rank probability curve and the entire plane of the plot. A smaller SUCRA value resulted in a lower incidence of adverse outcomes, indicating better efficacy of the treatment regimen. An examination of the assumption in the network meta-analysis includes homogeneity, transitivity and consistency. The examination of the homogeneity assumption was performed through direct treatment comparisons, and thus the χ^2^-based Q-test and I^2^ test were used for the analysis. The transitivity assumption was assessed by comparing the distribution of clinical variables, which were considered interfering factors that might affect the outcomes. The consistency assumption was tested to verify the feasibility of mixed comparisons. A design-by-treatment approach was used to assess inconsistency in the entire analytical network (18), and a loop-specific approach and node-splitting approach were used to assess local inconsistency.

We performed a number of sensitivity analyses to assess the robustness of primary outcomes, including: (1) well designed trials that did not fully meet the inclusion criteria (CAPRIE [Clopidogrel Versus Aspirin in Patients at Risk of Ischemic Events] (19), CHARISMA, COMMANDER HF [Effectiveness and Safety of Rivaroxaban in Reducing the Risk of Death, Myocardial Infarction, or Stroke in Participants with Heart Failure and Coronary Artery Disease Following an Episode of Decompensated Heart Failure] (20), and DAVID [Drug Evaluation in Atherosclerotic Vascular Disease in Diabetics] (21)) trials; (2) Trials where subgroup data met inclusion criteria but data from the entire study did not strictly meet inclusion criteria (DAPT [Dual Anti-platelet Therapy] (22, 23), THEMIS [Ticagrelor on Health Outcomes in Diabetes Mellitus Patients Intervention] (24), TWILIGHT [Ticagrelor with Aspirin or Alone in High-Risk Patients after Coronary Intervention] (25, 26), and VOYAGER PAD [Vascular Outcomes Study of ASA (acetylsalicylic acid) Along with Rivaroxaban in Endovascular or Surgical Limb Revascularization for PAD] (27, 28) trials); (3) Network meta-analysis structure combining ticagrelor and clopidogrel into P2Y12 inhibitor; (4) Network meta-analysis structure for accurate classification of major bleeding according to individual definitions; (5) Result of adjustment by person-years to reduce potential differences in follow-up time between trials. The verified data were analyzed using Stata software (version 15.0; Stata Corporation, College Station, TX), REVMAN software (version 5.3; Cochrane Collaboration, Oxford, UK), R software (version 3.6.3, the R Foundation, Vienna, Austria) and Word Processing System (version 2.5; Beijing, China).

## Results

### Literature search

Details of literature search, study exclusion, and selection are shown in Supplementary Figure 1 and Supplementary Table 2. After initial screening, 83 unique and full-text published articles remained. The full-text review of these 83 articles found 11 studies suitable for detailed review, of which 7 studies (5, 22, 24, 25, 27, 29, 30) met the inclusion criteria. The TWILIGHT COMPLEX trial (26) included patients with complex PCI, and 74% of them had multi-vessel CAD, and randomization for this cohort was set at 3 months after the index PCI and therefore this cohort was considered to have transitioned to stable status and met the inclusion criteria. Randomization to the VOYAGER PAD trial occurred nearly 10 days after revascularization, and these subjects were considered to have transitioned to stable condition and to meet the inclusion criteria. Four others (9, 19–21) (CHARISMA, COMMANDER HF, CAPRIE and DAVID) had different study designs. Approximately 36% of participants in the CHARISMA trial were stroke patients, and it was considered to have high clinical heterogeneity in safety assessments. The DAVID trial and the subgroups enrolled in the COMMANDER HF and the CAPRIE trial were missing results for the primary safety outcome. The imbalance in numbers for efficacy and safety outcomes was considered to affect the assessment of net clinical benefit, and these 4 studies were only included in the sensitivity analysis. As a result, the main analysis included 7 RCTs.

### Characteristics of the included studies and patients and study quality

The main characteristics of these studies are reported in Supplementary Table 3. The median follow-up time was 24 months. The included studies were all randomized, multicenter, double-blind placebo-controlled trials. The main clinical features of the patients are shown in Supplementary Table 4. A total of 111,737 patients were included in this NMA, of which the number for the main analysis was 83,529 patients.

The quality assessment of the included studies is presented in Supplementary Figure 2 and Supplementary Table 5. Briefly, the RCTs in the main analysis were all judged to have low risk of bias overall.

### Assumption and structure of network meta-analysis

**FIGURE 1:**
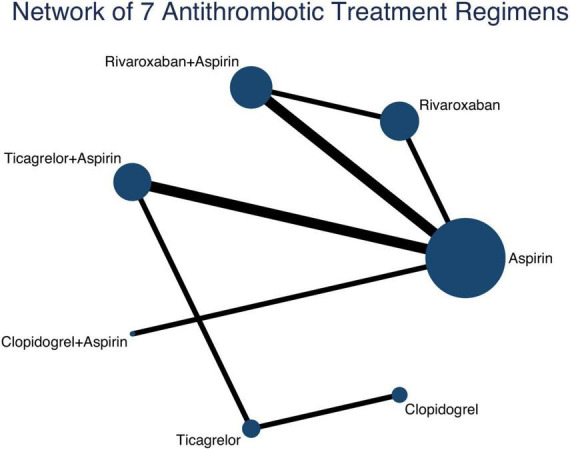
Network of 7 antithrombotic treatment regimens. Lines connect the interventions that have been studied in head-to-head (direct) comparisons in the eligible RCTs. The width of the lines represents the cumulative number of RCTs for each pairwise comparison and the size of every node is proportional to the number of randomized participants (sample size). RCTs, randomized controlled trials.

The results of homogeneity, transitivity, and consistency met the criteria for network meta-analysis, and details are provided in Supplementary Figures 3–5. Figure 1 shows the network of treatment regimens used in the analysis of the major efficacy outcome and major safety outcomes.

### Primary outcomes

#### MACEs

**FIGURE 2:**
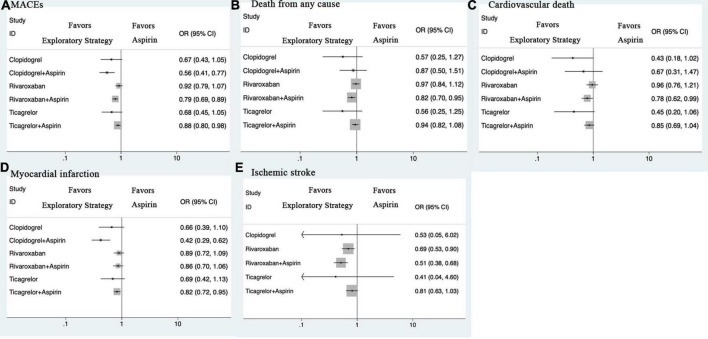
Forest plots for efficacy outcomes. **(A)** MACEs. **(B)** Death from any cause. **(C)** Cardiovascular death. **(D)** Myocardial infarction. **(E)** Ischemic stroke. CI, credible intervals; MACEs, major adverse cardiovascular and cerebrovascular events; OR, odds ratio.

Compared with aspirin monotherapy, rivaroxaban plus aspirin [OR 0.79 (0.69, 0.89)], ticagrelor plus aspirin [0.88 (0.80, 0.98)], and clopidogrel plus aspirin [0.56 (0.41, 0.77)] were associated with a reduced risk of MACEs (Figure 2 and Supplementary Table 6). Compared with rivaroxaban monotherapy, both rivaroxaban plus aspirin [0.86 (0.74, 1.00)] and clopidogrel plus aspirin [0.61 (0.43, 0.87)] were associated with a reduced risk of MACEs. Compared with rivaroxaban plus aspirin, clopidogrel plus aspirin [0.71 (0.51, 1.00)] was associated with a reduced risk of MACEs. Compared with ticagrelor plus aspirin, clopidogrel plus aspirin [0.64 (0.46, 0.89)] was associated with a reduced risk of MACEs.

### Major bleeding

**FIGURE 3:**
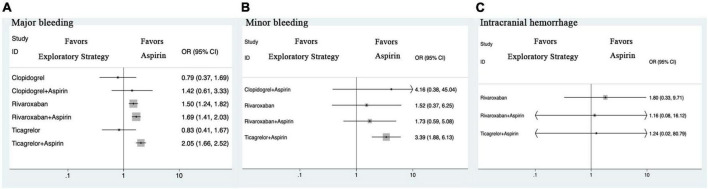
Forest plots for safety outcomes. **(A)** Major bleeding. **(B)** Minor bleeding. **(C)** Intracranial hemorrhage. CI, credible intervals; OR, odds ratio.

Compared with aspirin monotherapy, rivaroxaban monotherapy [OR 1.50 (1.24, 1.82)], rivaroxaban plus aspirin [1.69 (1.41, 2.03)], and ticagrelor plus aspirin [2.05 (1.66, 2.52)] were associated with a higher risk of major bleeding (Figure 3 and Supplementary Table 6). Compared with rivaroxaban monotherapy, ticagrelor plus aspirin [1.36 (1.03, 1.81)] was associated with a higher risk of major bleeding. Compared with ticagrelor plus aspirin, ticagrelor monotherapy [0.40 (0.21, 0.79)] and clopidogrel monotherapy [0.39 (0.19, 0.80)] were associated with a reduced risk of major bleeding.

### Net clinical benefit

**FIGURE 4:**
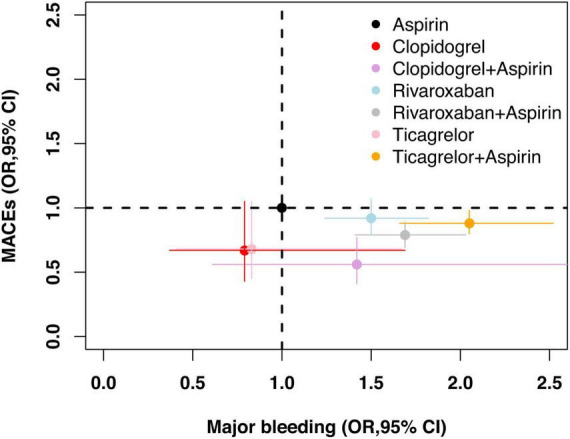
Net clinical benefit. OR of different antithrombotic strategies in comparison with aspirin (reference) and associated 95% CI are plotted. Major bleeding is on the *x*-axis and MACEs is on the *y*-axis. CI, credible intervals; MACEs, major adverse cardiovascular and cerebrovascular events; OR, odds ratio.

Figure 4 illustrates the risk of major bleeding versus MACEs for different antithrombotic strategies compared with aspirin monotherapy. The net clinical benefit favored clopidogrel monotherapy, followed by ticagrelor monotherapy, both of which are associated with a mild anti-ischemic effect and no increase in bleeding risk. Rivaroxaban plus aspirin, clopidogrel plus aspirin, and ticagrelor plus aspirin, although reducing the risk of MACEs, all increased the risk of major bleeding to varying degrees. In addition, treatment with rivaroxaban monotherapy tended to have a negative clinical benefit.

### Secondary outcomes

Compared with aspirin monotherapy, rivaroxaban plus aspirin was associated with a reduced risk of all-cause death (Figure 2 and Supplementary Table 6). Compared with rivaroxaban monotherapy, rivaroxaban plus aspirin was associated with a reduced risk of all-cause death. Compared with aspirin monotherapy, rivaroxaban plus aspirin was associated with a reduced risk of cardiovascular death. Compared with aspirin monotherapy, ticagrelor plus aspirin and clopidogrel plus aspirin were associated with a reduced risk of MI. Compared with rivaroxaban monotherapy, rivaroxaban plus aspirin, ticagrelor plus aspirin, and clopidogrel plus aspirin were associated with a reduced risk of MI. Compared with aspirin monotherapy, rivaroxaban monotherapy, rivaroxaban plus aspirin, and ticagrelor plus aspirin were associated with a reduced risk of ischemic stroke.

Compared with aspirin monotherapy, ticagrelor plus aspirin was associated with a higher risk of minor bleeding (Figure 3 and Supplementary Table 6). Compared with aspirin monotherapy, rivaroxaban monotherapy, rivaroxaban plus aspirin, and ticagrelor plus aspirin showed no statistically significant differences.

### Subgroup analysis

#### Outcomes in patients with CAD

**FIGURE 5:**
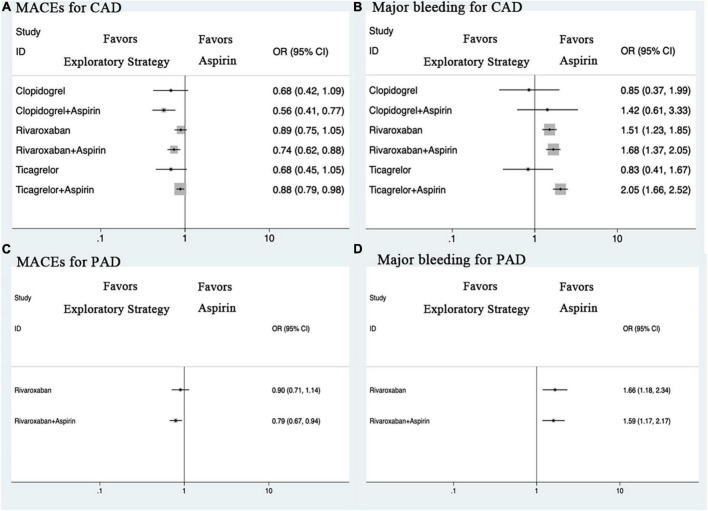
Forest plots for outcomes in patients with CAD or PAD. **(A)** MACEs for CAD. **(B)** Major bleeding for CAD. **(C)** MACEs for PAD. **(D)** Major bleeding for PAD. CAD, coronary artery disease; CI, credible intervals; MACEs, major adverse cardiovascular and cerebrovascular events; OR, odds ratio; PAD, peripheral artery disease.

Compared with aspirin monotherapy, rivaroxaban plus aspirin, ticagrelor plus aspirin, and clopidogrel plus aspirin were associated with a reduced risk of MACEs (Figure 5). Compared with rivaroxaban monotherapy, rivaroxaban plus aspirin and clopidogrel plus aspirin were associated with a reduced risk of MACEs. Compared with ticagrelor plus aspirin, clopidogrel plus aspirin was associated with a reduced risk of MACEs. For major bleeding, compared with aspirin monotherapy, rivaroxaban monotherapy, rivaroxaban plus aspirin, and ticagrelor plus aspirin were associated with a higher risk of major bleeding. Compared with rivaroxaban monotherapy, ticagrelor plus aspirin was associated with a higher risk of major bleeding. Compared with ticagrelor plus aspirin, ticagrelor monotherapy and clopidogrel monotherapy were associated with a reduced risk of major bleeding. For all-cause and cardiovascular death, the results for CAD were also similar to those of the overall cohort study. Sensitivity analyses yielded similar results (Supplementary Table 7 and Supplementary Appendix 1).

#### Outcomes in patients with PAD

Compared with aspirin monotherapy, rivaroxaban plus aspirin was associated with a reduced risk of MACEs (Figure 5). Sensitivity analysis showed that clopidogrel monotherapy was associated with a reduced risk of MACEs compared with aspirin monotherapy. For major bleeding, compared with aspirin monotherapy, rivaroxaban monotherapy and rivaroxaban plus aspirin were associated with a higher risk of major bleeding. Network meta-analysis for all-cause and cardiovascular death was not performed due to insufficient data (Supplementary Table 8 and Supplementary Appendix 1).

#### Ranking of treatment strategies and sensitivity analyses

Table 1 shows the SUCRA values for efficacy outcomes and safety outcomes. Clopidogrel plus aspirin (SUCRA of 9.0) was found to be the best strategy for reducing MACEs but was not very effective for limiting major bleeding (57.7). Clopidogrel monotherapy (15.0) and ticagrelor monotherapy (19.5) were considered the best strategies for reducing major bleeding and were considered very effective strategies for reducing MACEs (26.7 for clopidogrel, 31.5 for ticagrelor) (Supplementary Appendix 1).

**TABLE 1:** SUCRA values* for each treatment regimen and outcomes.

Value	Treatment regimen						
	Aspirin	Rivaroxaban	Rivaroxaban + Aspirin	Ticagrelor + Aspirin	Clopidogrel + Aspirin	Ticagrelor	Clopidogrel
**Efficacy outcome**							
MACEs	96.3	76.7	43.1	66.6	9.0	31.5	26.7
Death from any cause	82.6	72.7	35.7	65.2	54.2	18.5	21.1
Cardiovascular death	89.0	80.1	47.1	58.8	42.5	21.1	11.4
MI	94.6	68.7	62.9	53.5	2.5	39.1	28.7
Ischemic stroke	88.2	48.1	21.6	63.2	NA	26.6	52.3
**Safety outcome**							
Major bleeding	27.2	59.3	76.6	94.7	57.7	19.5	15.0
Minor bleeding	14.2	38.3	45.5	78.6	73.4	NA	NA
Intracranial hemorrhage	38.3	65.8	45.9	49.9	NA	NA	NA

MACEs, major adverse cardiovascular and cerebrovascular events; MI, myocardial infarction; NA, not available; SUCRA, surface under the cumulative ranking.

*SUCRA values are presented as percentage of area under the cumulative rank probability curve and the entire plane of the plot. The smaller the SUCRA value, the less incidence of adverse outcomes, which means the better the treatment regimen performance.

We obtained similar results from sensitivity analyses stratified by trial design, drug type, and bleeding definition. Details of the results are shown in Supplementary Table 9. The results after adjustment by person-years are consistent with the unadjusted results (Supplementary Appendix 2).

## Discussion

To our knowledge, this is the first network meta-analysis and strictly included SCVD patients at high ischemic risk, the results of which showed that compared with aspirin monotherapy, ticagrelor monotherapy and clopidogrel monotherapy appeared to be associated with reduced MACEs and no adverse effects on major bleeding. The effect of ticagrelor monotherapy and clopidogrel monotherapy on the risk of MACEs favored a reduced risk of cardiovascular death and MI. Dual antithrombotic therapy, including rivaroxaban plus aspirin, ticagrelor plus aspirin, and clopidogrel plus aspirin, has all been shown to reduce the risk of MACEs compared with aspirin monotherapy, but all have come at the cost of an increased risk of major bleeding. The results of the CAD and PAD subgroups were similar to those of the whole cohort. Additional sensitivity analyses were carried out, resulting in results similar to the main analysis.

Among patients with SCVD, a high proportion receive antithrombotic secondary prevention therapy, and previous studies suggested that low-dose aspirin can reduce ischemic outcomes in patients at a certain risk threshold (3). However, the limitations of aspirin monotherapy for preventing ischemic events in high-risk patients and numerous adverse effects have been gradually uncovered. Various exploratory antithrombotic regimens have been investigated as alternatives to aspirin for long-term cardiovascular prevention. This network meta-analysis aimed to explore the efficacy and safety of different antithrombotic strategies in a “COMPASS-like,” high ischemic risk secondary prevention population. The recent COMPASS trial (5) demonstrated a net clinical benefit of rivaroxaban plus aspirin compared with aspirin monotherapy in such a high-risk ischemic population. The VOYAGER PAD trial (27), which was designed to assess the addition of rivaroxaban to aspirin in the endovascular or surgical limb revascularization of PAD, similarly showed that the addition of rivaroxaban to aspirin reduced the risk of MACEs more than aspirin alone and was associated with an increased risk of bleeding. The COMMANDER HF trial (20) was designed to assess the clinical benefit of adding rivaroxaban in patients with CAD and worsening chronic heart failure (HF), but the results showed that the addition of rivaroxaban (rivaroxaban plus aspirin versus aspirin in 60% of the population) did not reduce the risk of MACEs and was associated with an increased risk of bleeding. Possible explanations for this inconsistency include differences in the characteristics of the included populations. COMMANDER HF severely limited cardiac function in patients (low ejection fraction with recent deterioration of cardiac function), and adverse effects in HF that cannot be ameliorated by the antithrombotic effects of rivaroxaban. Additionally, although arms such as rivaroxaban plus aspirin, rivaroxaban plus thienopyridines, and rivaroxaban plus dual antiplatelets were included in the subgroup analysis of COMMANDER HF, the trial was designed primarily to add-on treatment to rivaroxaban and lacked a rigorous randomization design for other antiplatelet agents.

Dual antiplatelet therapy is considered necessary in patients with acute coronary syndrome (ACS) or PCI, but its long-term use in patients with SCAD or PAD is controversial. Previous network meta-analyses (31, 32) aimed to explore the optimal treatment strategy for dual antiplatelet therapy after PCI, and the results showed that an extended-term dual antiplatelet regimen, although reducing the risk of ischaemic events at the cost of more frequent major bleeding, did not result in significant net clinical benefit. The bidirectional nature of the clinical benefit of dual antiplatelet therapy for a broad CCS population was also demonstrated in our previous network meta-analysis (8), which included 3 trials of dual antiplatelet therapy. The PEGASUS-TIMI 54 [Prevention of Cardiovascular Events in Patients with Prior Heart Attack Using Ticagrelor Compared to Placebo on a Background of Aspirin - Thrombolysis in Myocardial Infarction 54] trial (29) was designed to explore the clinical benefit of a dose of ticagrelor 60 or 90 mg twice a day in patients with SCAD with more than one year of MI, whereas the THEMIS trial (24) was designed to explore the effect of ticagrelor 90 mg subsequently switched to a dose of 60 mg twice a day for secondary prevention in patients with SCAD with diabetes. Both the PEGASUS-TIMI 54 and THEMIS trials showed that ticagrelor combined with aspirin reduced the risk of MACEs while increasing the risk of major bleeding. Another DAPT trial (22) designed to explore the clinical benefit of continuing thienopyridines in patients with SCAD one year after medical stenting yielded similar results.

The results of our present network meta-analysis showed that clopidogrel plus aspirin appeared to have a superior anti-ischemic effect to ticagrelor plus aspirin in the main analysis, and a possible explanation for this inconsistency with the ACS cohort is that early and permanent discontinuation of medications reached more than 30% in the PEGASUS-TIMI 54 and THEMIS trials. The causes were mainly adverse effects such as dyspnea or bleeding. Additional studies were added to a sensitivity analysis showing similar anti-ischemic effects. Clopidogrel plus aspirin also appeared to have superior anti-ischemic effects to rivaroxaban plus aspirin in the main analysis, but after the addition of the studies by CHARISMA et al. as for a sensitivity analysis, it was shown that the anti-ischemic effects were similar. Both P2Y12 inhibitors plus aspirin and rivaroxaban plus aspirin could reduce the risk of MACEs, but the former might have a reduced risk of MI, whereas the latter might have a reduced risk of all-cause or cardiovascular death or stroke. Ticagrelor plus aspirin was considered the worst strategy for limiting major or minor bleeding.

It seems clear that dual antithrombotic therapy reduces ischemia but increases bleeding risk. Other antithrombotic regimens alone have also been tested to replace aspirin as secondary prevention. The TWILIGHT trial (25), which was designed to explore secondary prevention in patients with CAD and in which randomization was set at 3 months after PCI, showed that ticagrelor monotherapy had a better safety profile that was not accompanied by an increased risk of ischemia compared with ticagrelor plus aspirin. The CAPRIE trial (19) is among the non-contemporary studies, but in which the clinical characteristics of the PAD subgroup were similar to contemporary studies. Its results showed that the anti-ischemic effect of clopidogrel alone was superior to that of aspirin. The EUCLID [Examining Use of Ticagrelor in Peripheral Artery Disease] trial (30, 33), which was designed to explore the clinical benefit of ticagrelor and clopidogrel in symptomatic PAD patients at high ischemic risk, found that the overall benefit was similar between the two medications, but clopidogrel was associated with higher medication adherence than ticagrelor, mainly due to adverse effects such as dyspnea or bleeding in the latter. The recently published HOST-EXAM [HOST-EXtended Antiplatelet Monotherapy] trial (34), which enrolled a broad group of patients post PCI and in stable state to compare the net clinical benefit of aspirin versus clopidogrel alone, found that clopidogrel was superior to aspirin, with similar outcomes remaining in the multivessel or MI subgroup, However, because the outcome of the subgroup meeting the inclusion criteria was not available, it was not included in this present quantitative analysis.

Our main analysis revealed that ticagrelor monotherapy and clopidogrel monotherapy were considered to be similar to aspirin in safety, and even the SUCRA values indicated that they had a better safety profile than aspirin. Ticagrelor monotherapy and clopidogrel monotherapy were associated with a mild anti-ischemic effect compared with aspirin monotherapy. A sensitivity analysis of 11 studies showed that this moderated anti-ischemic effect persisted.

This present network meta-analysis is not considered as an updated analysis of our previous study (8). Compared with our previous analysis, this study strictly included SCVD patients with high ischemic risk for the first time, including those at high ischemic risk, such as CAD with multivessel disease or history of MI, symptomatic PAD or limb revascularization or amputation, or asymptomatic carotid stenosis. These included populations represents a larger CVD cohort than CCS but at the same time has a clear definition of high ischemic risk. Second, the present analysis incorporated more treatment arms, enriched network architecture, and our results found that ticagrelor monotherapy and clopidogrel monotherapy appeared to have the best net clinical benefit. Previous meta-analyses (31, 32, 35, 36) focused mainly on secondary prevention strategies in populations one to two years after ACS or PCI, with results favoring dual antiplatelet therapy followed by P2Y12 inhibitor monotherapy to reduce the risk of major bleeding without an increased risk of ischemic events. Our results may at the same time provide further evidence for more distant secondary prevention in this CVD cohort.

### Limitations

The study has some limitations. First, although clear statistical heterogeneity was not observed in our analysis and strict inclusion criteria greatly reduced the clinical heterogeneity, some clinical heterogeneity was identified among the studies, with potential sources including exclusion criteria, definition of outcomes, treatment dose and course, and follow-up time, which may affect the interpretation of our results. Second, few trials with the same exploratory treatment group were included in this analysis, likely due to strict inclusion criteria, but the overall high-quality and large sample cluster suggests that the quality of evidence was good. Third, there were fewer trials on PAD that met the criteria than CAD, which limited the structure of the NMA of PAD, and more studies with more similarities are needed in the future to provide more robust results. In addition, the network meta-analysis in part uses indirect evidence between multiple therapies, allowing comparisons to be made where direct trial evidence is limited. This approach respects randomization but still does not reach the level of randomized evidence.

## Conclusion

In SCVD populations at high ischemic risk, such as CAD with multi-vessel disease or history of MI, symptomatic PAD or limb revascularization or amputation, ticagrelor plus aspirin, clopidogrel plus aspirin, or rivaroxaban plus aspirin reduced MACEs but increased the risk of major bleeding, with ticagrelor plus aspirin having the highest risk of bleeding. The net clinical benefit appeared to favor either ticagrelor monotherapy or clopidogrel monotherapy regimens. Overall, in patients with complex chronic coronary syndrome or peripheral artery disease at high risk of ischemia, P2Y12 inhibitors alone are the first treatment recommended, excluding patients with recurrent MACEs, but more importantly, individualized treatment regimens after considering the relative and absolute risks of ischemia and bleeding for each patient.

## Data availability statement

The original contributions presented in this study are included in the article/Supplementary material, further inquiries can be directed to the corresponding authors.

## Author contributions

JH and TC conceived and designed the study. HZ and XX performed the study. HZ, XX, and JZ verified the data. HZ, HW, and BG analyzed the data. XX, GT, and LZ drafted the manuscript. XF, QC, and TC performed critical revision for the manuscript. All authors approved the final version of the manuscript.
