# Supplementary material for: Secondary prevention of antithrombotic therapy in patients with stable cardiovascular disease at high ischemic risk: A network meta-analysis of randomized controlled trials
Source: Front Cardiovasc Med. 2023 Jan 9;9:1040473. doi: 10.3389/fcvm.2022.1040473 (PMC9869170; doi:10.3389/fcvm.2022.1040473)
Supplement: Supplementary file 1 [file Data_Sheet_1.DOCX]

**Supplementary Online Content**

Secondary prevention of antithrombotic therapy in patients with stable cardiovascular disease at high ischemic risk: a network meta-analysis of randomized controlled trials

[Data Collection and Quality Evaluation 3](#_Toc532847960)

[eTable 1. PRISMA checklist 4](#_Toc568855730)

[eTable 2. Retrieval and filtering 6](#_Toc161057666)

[eFigure 1. Flow diagram of study search and selection. 18](#_Toc1066797242)

[eTable 3. Main characteristics of trials included in the meta-analysis 19](#_Toc320277491)

[eTable 4. Main characteristics of patients enrolled among trials included in the meta-analysis 23](#_Toc1309771855)

[eFigure 2. Risk of bias of included trials using the Cochrane risk assessment tool 24](#_Toc1628185235)

[eTable 5. Evaluation of risk of bias of included trials 25](#_Toc1672614571)

[eFigure 3. Homogeneity assumption in network meta-analysis 30](#_Toc1072155567)

[eFigure 4. Transitivity assumption in network meta-analysis 32](#_Toc183332592)

[eFigure 5. Consistency assumption in network meta-analysis 34](#_Toc1779323946)

[Additional results 36](#_Toc1387775947)

[eTable 6. Pairwise comparison of efficacy and safety outcomes for the main analysis in the total cohort 37](#_Toc530451162)

[eTable 7. Pairwise comparison of efficacy and safety outcomes in patients with coronary artery disease 39](#_Toc1088061037)

[eTable 8. Pairwise comparison of efficacy and safety outcomes in patients with peripheral artery disease 40](#_Toc1218594654)

[eAppendix 1. Cumulative rank probability plot for efficacy and safety outcomes 41](#_Toc368808339)

[eTable 9. Sensitivity analyses for pairwise comparison of efficacy and safety outcomes in the total cohort 60](#_Toc923948331)

[eAppendix 2. Sensitivity analyses for adjustment of primary efficacy and safety outcomes by person-years in the total cohort 64](#_Toc345347660)

# **Data Collection and Quality Evaluation**

## eTable 1. PRISMA checklist

##

**PRISMA NMA Checklist of Items to Include When Reporting A Systematic Review Involving a Network Meta-analysis**

| **Section/Topic** | **Item #** | **Checklist Item** | **Reported on Page #** |
| --- | --- | --- | --- |
| **TITLE** |  |  |  |
| Title | 1 | Identify the report as a systematic review *incorporating a network meta-analysis (or related form of meta-analysis).* | 1 |
|  |  |  |  |
| **ABSTRACT** |  |  |  |
| Structured summary | 2 | Provide a structured summary including, as applicable:  **Background:** main objectives  **Methods:** data sources; study eligibility criteria, participants, and interventions; study appraisal; and *synthesis methods, such as network meta-analysis.*  **Results:** number of studies and participants identified; summary estimates with corresponding confidence/credible intervals; *treatment rankings may also be discussed. Authors may choose to summarize pairwise comparisons against a chosen treatment included in their analyses for brevity.*  **Discussion/Conclusions:** limitations; conclusions and implications of findings.  **Other:** primary source of funding; systematic review registration number with registry name. | 3-4 |
|  |  |  |  |
| **INTRODUCTION** |  |  |  |
| Rationale | 3 | Describe the rationale for the review in the context of what is already known*, including mention of why a network meta-analysis has been conducted.* | 5 |
| Objectives | 4 | Provide an explicit statement of questions being addressed, with reference to participants, interventions, comparisons, outcomes, and study design (PICOS). | 5 |
|  |  |  |  |
| **METHODS** |  |  |  |
| Protocol and registration | 5 | Indicate whether a review protocol exists and if and where it can be accessed (e.g., Web address); and, if available, provide registration information, including registration number. | 6 |
| Eligibility criteria | 6 | Specify study characteristics (e.g., PICOS, length of follow-up) and report characteristics (e.g., years considered, language, publication status) used as criteria for eligibility, giving rationale. *Clearly describe eligible treatments included in the treatment network, and note whether any have been clustered or merged into the same node (with justification).* | 6-7 |
| Information sources | 7 | Describe all information sources (e.g., databases with dates of coverage, contact with study authors to identify additional studies) in the search and date last searched. | 6 |
| Search | 8 | Present full electronic search strategy for at least one database, including any limits used, such that it could be repeated. | 6-7 |
| Study selection | 9 | State the process for selecting studies (i.e., screening, eligibility, included in systematic review, and, if applicable, included in the meta-analysis). | 6-7 |
| Data collection process | 10 | Describe method of data extraction from reports (e.g., piloted forms, independently, in duplicate) and any processes for obtaining and confirming data from investigators. | 6-7 |
| Data items | 11 | List and define all variables for which data were sought (e.g., PICOS, funding sources) and any assumptions and simplifications made. | 6-7 |
| **Geometry of the network** | **S1** | Describe methods used to explore the geometry of the treatment network under study and potential biases related to it. This should include how the evidence base has been graphically summarized for presentation, and what characteristics were compiled and used to describe the evidence base to readers. | 7-9 |
| Risk of bias within individual studies | 12 | Describe methods used for assessing risk of bias of individual studies (including specification of whether this was done at the study or outcome level), and how this information is to be used in any data synthesis. | 7-9 |
| Summary measures | 13 | State the principal summary measures (e.g., risk ratio, difference in means). *Also describe the use of additional summary measures assessed, such as treatment rankings and surface under the cumulative ranking curve (SUCRA) values, as well as modified approaches used to present summary findings from meta-analyses.* | 7-9 |
| Planned methods of analysis | 14 | Describe the methods of handling data and combining results of studies for each network meta-analysis. This should include, but not be limited to:   - *Handling of multi-arm trials;* - *Selection of variance structure;* - *Selection of prior distributions in Bayesian analyses; and* - *Assessment of model fit.* | 8 |
| **Assessment of Inconsistency** | **S2** | Describe the statistical methods used to evaluate the agreement of direct and indirect evidence in the treatment network(s) studied. Describe efforts taken to address its presence when found. | 7-8 |
| Risk of bias across studies | 15 | Specify any assessment of risk of bias that may affect the cumulative evidence (e.g., publication bias, selective reporting within studies). | 6-7 |
| Additional analyses | 16 | Describe methods of additional analyses if done, indicating which were pre-specified. This may include, but not be limited to, the following:   - Sensitivity or subgroup analyses; - Meta-regression analyses; - *Alternative formulations of the treatment network; and* - *Use of alternative prior distributions for Bayesian analyses (if applicable).* | 7-9 |
|  |  |  |  |
| **RESULTS†** |  |  |  |
| Study selection | 17 | Give numbers of studies screened, assessed for eligibility, and included in the review, with reasons for exclusions at each stage, ideally with a flow diagram. | 9 |
| **Presentation of network structure** | **S3** | Provide a network graph of the included studies to enable visualization of the geometry of the treatment network. | 10 |
| **Summary of network geometry** | **S4** | Provide a brief overview of characteristics of the treatment network. This may include commentary on the abundance of trials and randomized patients for the different interventions and pairwise comparisons in the network, gaps of evidence in the treatment network, and potential biases reflected by the network structure. | 10 |
| Study characteristics | 18 | For each study, present characteristics for which data were extracted (e.g., study size, PICOS, follow-up period) and provide the citations. | 9-10 |
| Risk of bias within studies | 19 | Present data on risk of bias of each study and, if available, any outcome level assessment. | 9-10 |
| Results of individual studies | 20 | For all outcomes considered (benefits or harms), present, for each study: 1) simple summary data for each intervention group, and 2) effect estimates and confidence intervals. *Modified approaches may be needed to deal with information from larger networks.* | 10-11 |
| Synthesis of results | 21 | Present results of each meta-analysis done, including confidence/credible intervals. *In larger networks, authors may focus on comparisons versus a particular comparator (e.g. placebo or standard care), with full findings presented in an appendix. League tables and forest plots may be considered to summarize pairwise comparisons.* If additional summary measures were explored (such as treatment rankings), these should also be presented. | 10-13 |
| **Exploration for inconsistency** | **S5** | Describe results from investigations of inconsistency. This may include such information as measures of model fit to compare consistency and inconsistency models, *P* values from statistical tests, or summary of inconsistency estimates from different parts of the treatment network. | 10 |
| Risk of bias across studies | 22 | Present results of any assessment of risk of bias across studies for the evidence base being studied. | 9-10 |
| Results of additional analyses | 23 | Give results of additional analyses, if done (e.g., sensitivity or subgroup analyses, meta-regression analyses*, alternative network geometries studied, alternative choice of prior distributions for Bayesian analyses,* and so forth). | 12-13 |
|  |  |  |  |
| **DISCUSSION** |  |  |  |
| Summary of evidence | 24 | Summarize the main findings, including the strength of evidence for each main outcome; consider their relevance to key groups (e.g., healthcare providers, users, and policy-makers). | 13-16 |
| Limitations | 25 | Discuss limitations at study and outcome level (e.g., risk of bias), and at review level (e.g., incomplete retrieval of identified research, reporting bias). *Comment on the validity of the assumptions, such as transitivity and consistency. Comment on any concerns regarding network geometry (e.g., avoidance of certain comparisons).* | 16-17 |
| Conclusions | 26 | Provide a general interpretation of the results in the context of other evidence, and implications for future research. | 17 |
|  |  |  |  |
| **FUNDING** |  |  |  |
| Funding | 27 | Describe sources of funding for the systematic review and other support (e.g., supply of data); role of funders for the systematic review. This should also include information regarding whether funding has been received from manufacturers of treatments in the network and/or whether some of the authors are content experts with professional conflicts of interest that could affect use of treatments in the network. | 20 |

PICOS = population, intervention, comparators, outcomes, study design.

* Text in italics indicateS wording specific to reporting of network meta-analyses that has been added to guidance from the PRISMA statement.

† Authors may wish to plan for use of appendices to present all relevant information in full detail for items in this section.

## eTable 2. Retrieval and filtering

Search code

| Data search | Duplicates | Total |
| --- | --- | --- |
| 1. Pubmed | 0 | +1725 |
| 2. Embase | 1272 | +1040 |
| 3. Cochrane | 1349 | +33 |
|  |  | 2798 articles to screen |

PubMed/MEDLINE search:

| Name search | Search | PubMed query | Results |
| --- | --- | --- | --- |
| ASCVD or CAD or PAD | #1 | (((((((((((((((((((((((atherosclerotic cardiovascular disease[Title/Abstract]) OR (atherosclerotic vascular disease[Title/Abstract])) OR (coronary heart disease[Title/Abstract])) OR (coronary artery atherosclerosis[Title/Abstract])) OR (coronary artery disease[Title/Abstract])) OR (coronary disease[Title/Abstract])) OR (coronary artery bypass graft surgery[Title/Abstract])) OR (prior myocardial infarction[Title/Abstract])) OR (prior acute coronary syndrome[Title/Abstract])) OR (prior revascularization[Title/Abstract])) OR (prior percutaneous coronary intervention[Title/Abstract])) OR (prior stent[Title/Abstract])) OR (angina[Title/Abstract])) OR (chronic coronary syndrome[Title/Abstract])) OR (peripheral artery disease[Title/Abstract])) OR (arterial vascular disease[Title/Abstract])) OR (aorto-femoral bypass surgery[Title/Abstract])) OR (limb bypass surgery[Title/Abstract])) OR (percutaneous transluminal angioplasty revascularization[Title/Abstract])) OR (limb[Title/Abstract] OR foot amputation[Title/Abstract])) OR (intermittent claudication[Title/Abstract])) OR (carotid revascularization[Title/Abstract])) OR (carotid artery stenosis[Title/Abstract])) OR (((((coronary artery disease[MeSH Terms]) OR (coronary artery bypass graft surgery[MeSH Terms])) OR (chronic coronary syndrome[MeSH Terms])) OR (peripheral artery disease[MeSH Terms])) OR (carotid artery stenosis[MeSH Terms])) | 455,843 |
| Antiplatelet therapy | #2 | (((((((((ticlopidine[MeSH Terms]) OR (clopidogrel[MeSH Terms])) OR (prasugrel[MeSH Terms])) OR (ticagrelor[MeSH Terms])) OR (cangrelor[MeSH Terms])) OR (tirofiban[MeSH Terms])) OR (vorapaxar[MeSH Terms])) OR (dipyridamole[MeSH Terms])) OR (cilostazol[MeSH Terms])) OR (((((((((((((((P2Y12 inhibitors[Title/Abstract]) OR (ticlopidine[Title/Abstract])) OR (clopidogrel[Title/Abstract])) OR (prasugrel[Title/Abstract])) OR (ticagrelor[Title/Abstract])) OR (cangrelor[Title/Abstract])) OR (GPIIb/IIIa inhibitors[Title/Abstract])) OR (tirofiban[Title/Abstract])) OR (abciximab[Title/Abstract])) OR (PAR-1 inhibitors[Title/Abstract])) OR (vorapaxar[Title/Abstract])) OR (atopaxar[Title/Abstract])) OR (phosphodiesterase inhibitors[Title/Abstract])) OR (dipyridamole[Title/Abstract])) OR (cilostazol[Title/Abstract])) | 35,956 |
| Anticoagulant therapy | #3 | ((((((((((((vitamin K antagonists[Title/Abstract]) OR (warfarin[Title/Abstract])) OR (new oral anticoagulants[Title/Abstract])) OR (dabigatran[Title/Abstract])) OR (factor Xa inhibitor[Title/Abstract])) OR (rivaroxaban[Title/Abstract])) OR (apixaban[Title/Abstract])) OR (edoxaban[Title/Abstract])) OR (warfarin[MeSH Terms])) OR (dabigatran[MeSH Terms])) OR (rivaroxaban[MeSH Terms])) OR (apixaban[MeSH Terms])) OR (edoxaban[MeSH Terms]) | 41,736 |
| Combined search |  | #1 AND (#2 OR #3) AND (Clinical Trial OR Randomized Controlled Trial) | 1,725 |

OVID/EMBASE Search:

| Name search | Search | EMBASE query | Results |
| --- | --- | --- | --- |
| ASCVD or CAD or PAD | #1 | 'atherosclerotic cardiovascular disease':ab,ti OR 'atherosclerotic vascular disease':ab,ti OR 'coronary heart disease':ab,ti OR 'coronary artery atherosclerosis':ab,ti OR 'coronary artery disease':ab,ti OR 'coronary artery bypass graft surgery':ab,ti OR 'prior myocardial infarction':ab,ti OR 'prior acute coronary syndrome':ab,ti OR 'prior revascularization':ab,ti OR 'prior percutaneous coronary intervention':ab,ti OR 'prior stent':ab,ti OR angina:ab,ti OR 'chronic coronary syndrome':ab,ti OR 'peripheral artery disease':ab,ti OR 'arterial vascular disease':ab,ti OR 'aorto-femoral bypass surgery':ab,ti OR 'limb bypass surgery':ab,ti OR 'percutaneous transluminal angioplasty revascularization':ab,ti OR limb:ab,ti OR 'foot amputation':ab,ti OR 'intermittent claudication':ab,ti OR 'carotid revascularization':ab,ti OR 'carotid artery stenosis':ab,ti | 508,699 |
| Antiplatelet therapy | #2 | aspirin:ab,ti OR 'p2y12 inhibitors':ab,ti OR ticlopidine:ab,ti OR clopidogrel:ab,ti OR prasugrel:ab,ti OR ticagrelor:ab,ti OR cangrelor:ab,ti OR 'gpiib/iiia inhibitors':ab,ti OR tirofiban:ab,ti OR abciximab:ab,ti OR 'par-1 inhibitors':ab,ti OR vorapaxar:ab,ti OR atopaxar:ab,ti OR 'phosphodiesterase inhibitor':ab,ti OR dipyridamole:ab,ti OR 'arterial vascular disease':ab,ti OR cilostazol:ab,ti | 112,294 |
| Anticoagulant therapy | #3 | 'vitamin k antagonists':ab,ti OR warfarin:ab,ti OR 'new oral anticoagulants':ab,ti OR dabigatran:ab,ti OR 'factor xa inhibitor':ab,ti OR rivaroxaban:ab,ti OR apixaban:ab,ti OR edoxaban:ab,ti | 60,034 |
| Combined search |  | #1 AND (#2 OR #3) AND ([controlled clinical trial]/lim OR [randomized controlled trial]/lim) | 2,312 |

Cochrane Database search:

| Name search | Search | Cochrane query | Results |
| --- | --- | --- | --- |
| ASCVD or CAD or PAD | #6= #1 or #2 or #3 or #4 or #5 | #1 = (atherosclerotic cardiovascular disease):ti,ab,kw OR (atherosclerotic vascular disease):ti,ab,kw OR (coronary heart disease):ti,ab,kw OR (coronary artery atherosclerosis):ti,ab,kw OR (coronary artery disease):ti,ab,kw  #2 - (coronary disease):ti,ab,kw OR (coronary artery bypass graft surgery):ti,ab,kw OR (prior myocardial infarction):ti,ab,kw OR (prior acute coronary syndrome):ti,ab,kw OR (prior revascularization):ti,ab,kw  #3 = (prior percutaneous coronary intervention):ti,ab,kw OR (prior stent):ti,ab,kw OR (angina):ti,ab,kw OR (chronic coronary syndrome):ti,ab,kw OR (peripheral artery disease):ti,ab,kw  #4 = (arterial vascular disease):ti,ab,kw OR (aorto-femoral bypass surgery):ti,ab,kw OR (limb bypass surgery):ti,ab,kw OR (percutaneous transluminal angioplasty revascularization):ti,ab,kw OR (limb or foot amputation):ti,ab,kw  #5 = (intermittent claudication):ti,ab,kw OR (carotid revascularization):ti,ab,kw OR (carotid artery stenosis):ti,ab,kw | 86,862 |
| Antiplatelet therapy | #10= #7 or #8 or #9 | #7 = (aspirin):ti,ab,kw OR (P2Y12 inhibitors):ti,ab,kw OR (ticlopidine):ti,ab,kw OR (clopidogrel):ti,ab,kw OR (prasugrel):ti,ab,kw  #8 = (PAR-1 inhibitors):ti,ab,kw OR (vorapaxar):ti,ab,kw OR (atopaxar):ti,ab,kw OR (phosphodiesterase inhibitors):ti,ab,kw OR (dipyridamole):ti,ab,kw  #9 = (ticagrelor):ti,ab,kw OR (cangrelor):ti,ab,kw OR (tirofiban):ti,ab,kw OR (abciximab):ti,ab,kw OR (cilostazol):ti,ab,kw | 23,100 |
| Anticoagulant therapy | #13= #11 or #12 | #11 = (vitamin K antagonists):ti,ab,kw OR ("Warfarin"):ti,ab,kw OR (new oral anticoagulants):ti,ab,kw OR (dabigatran):ti,ab,kw OR (factor Xa inhibitor):ti,ab,kw  #12 = (rivaroxaban):ti,ab,kw OR (apixaban):ti,ab,kw OR (edoxaban):ti,ab,kw | 8,289 |
| Combined search |  | #6 and (#10 or #13) AND (Clinical Trial or Randomized Controlled Trial) | 1,382 |

Articles excluded after full text screening:

| Number | Excluded references | Reason for exclusion |
| --- | --- | --- |
| 1 | Eisen A, Harrington RA, Stone GW, Steg PG, Gibson CM, Hamm CW, Price MJ, Prats J, Deliargyris EN, Mahaffey KW, et al. Cangrelor compared with clopidogrel in patients with prior myocardial infarction - Insights from the CHAMPION trials. *INT J CARDIOL.* 2018;250:49-55. | Failure to meet a “COMPASS-like” inclusion criteria |
| 2 | Wiviott SD, Braunwald E, McCabe CH, Montalescot G, Ruzyllo W, Gottlieb S, Neumann FJ, Ardissino D, De Servi S, Murphy SA, et al. Prasugrel versus clopidogrel in patients with acute coronary syndromes. *N Engl J Med.* 2007;357(20):2001-2015. | Failure to meet a “COMPASS-like” inclusion criteria |
| 3 | Roe MT, Armstrong PW, Fox KA, White HD, Prabhakaran D, Goodman SG, Cornel JH, Bhatt DL, Clemmensen P, Martinez F, et al. Prasugrel versus clopidogrel for acute coronary syndromes without revascularization. *N Engl J Med.* 2012;367(14):1297-1309. | Failure to meet a “COMPASS-like” inclusion criteria |
| 4 | Orme RC, Parker W, Thomas MR, Judge HM, Baster K, Sumaya W, Morgan KP, McMellon HC, Richardson JD, Grech ED, et al. Study of Two Dose Regimens of Ticagrelor Compared with Clopidogrel in Patients Undergoing Percutaneous Coronary Intervention for Stable Coronary Artery Disease (STEEL-PCI). *CIRCULATION.* 2018;138(13):1290-1300. | Endpoint events of no interest |
| 5 | Ohman EM, Roe MT, Steg PG, James SK, Povsic TJ, White J, Rockhold F, Plotnikov A, Mundl H, Strony J, et al. Clinically significant bleeding with low-dose rivaroxaban versus aspirin, in addition to P2Y12 inhibition, in acute coronary syndromes (GEMINI-ACS-1): a double-blind, multicentre, randomised trial. *LANCET.* 2017;389(10081):1799-1808. | Failure to meet a “COMPASS-like” inclusion criteria |
| 6 | Bohula EA, Aylward PE, Bonaca MP, Corbalan RL, Kiss RG, Murphy SA, Scirica BM, White H, Braunwald E, Morrow DA. Efficacy and Safety of Vorapaxar With and Without a Thienopyridine for Secondary Prevention in Patients With Previous Myocardial Infarction and No History of Stroke or Transient Ischemic Attack: Results from TRA 2°P-TIMI 50. *CIRCULATION.* 2015;132(20):1871-1879. | Failure to meet a “COMPASS-like” inclusion criteria |
| 7 | Morrow DA, Braunwald E, Bonaca MP, Ameriso SF, Dalby AJ, Fish MP, Fox KA, Lipka LJ, Liu X, Nicolau JC, et al. Vorapaxar in the secondary prevention of atherothrombotic events. *N Engl J Med.* 2012;366(15):1404-1413. | Failure to meet a “COMPASS-like” inclusion criteria |
| 8 | Bonaca MP, Scirica BM, Creager MA, Olin J, Bounameaux H, Dellborg M, Lamp JM, Murphy SA, Braunwald E, Morrow DA. Vorapaxar in patients with peripheral artery disease: results from TRA2{degrees}P-TIMI 50. *CIRCULATION.* 2013;127(14):1522-1529, 1521e-1529e. | Antithrombotic regimens difficult to be included |
| 9 | Alexander JH, Lopes RD, James S, Kilaru R, He Y, Mohan P, Bhatt DL, Goodman S, Verheugt FW, Flather M, et al. Apixaban with antiplatelet therapy after acute coronary syndrome. *N Engl J Med.* 2011;365(8):699-708. | Failure to meet a “COMPASS-like” inclusion criteria |
| 10 | Hurlen M, Abdelnoor M, Smith P, Erikssen J, Arnesen H. Warfarin, aspirin, or both after myocardial infarction. *N Engl J Med.* 2002;347(13):969-974. | Failure to meet a “COMPASS-like” inclusion criteria |
| 11 | Mega JL, Braunwald E, Wiviott SD, Bassand JP, Bhatt DL, Bode C, Burton P, Cohen M, Cook-Bruns N, Fox KA, et al. Rivaroxaban in patients with a recent acute coronary syndrome. *N Engl J Med.* 2012;366(1):9-19. | Failure to meet a “COMPASS-like” inclusion criteria |
| 12 | Vranckx P, Valgimigli M, Jüni P, Hamm C, Steg PG, Heg D, van Es GA, McFadden EP, Onuma Y, van Meijeren C, et al. Ticagrelor plus aspirin for 1 month, followed by ticagrelor monotherapy for 23 months vs aspirin plus clopidogrel or ticagrelor for 12 months, followed by aspirin monotherapy for 12 months after implantation of a drug-eluting stent: a multicentre, open-label, randomised superiority trial. *LANCET.* 2018;392(10151):940-949.. | Failure to meet a “COMPASS-like” inclusion criteria |
| 13 | Moll F, Baumgartner I, Jaff M, Nwachuku C, Tangelder M, Ansel G, Adams G, Zeller T, Rundback J, Grosso M, et al. Edoxaban Plus Aspirin vs Dual Antiplatelet Therapy in Endovascular Treatment of Patients With Peripheral Artery Disease: Results of the ePAD Trial. *J ENDOVASC THER.* 2018;25(2):158-168. | Failure to meet a “COMPASS-like” inclusion criteria |
| 14 | Belch JJ, Dormandy J, Biasi GM, Cairols M, Diehm C, Eikelboom B, Golledge J, Jawien A, Lepäntalo M, Norgren L, et al. Results of the randomized, placebo-controlled clopidogrel and acetylsalicylic acid in bypass surgery for peripheral arterial disease (CASPAR) trial. *J VASC SURG.* 2010;52(4):825-833, 831-833. | Failure to meet a “COMPASS-like” inclusion criteria |
| 15 | Belch J, MacCuish A, Campbell I, Cobbe S, Taylor R, Prescott R, Lee R, Bancroft J, MacEwan S, Shepherd J, et al. The prevention of progression of arterial disease and diabetes (POPADAD) trial: factorial randomised placebo controlled trial of aspirin and antioxidants in patients with diabetes and asymptomatic peripheral arterial disease. *BMJ.* 2008;337:a1840. | Failure to meet a “COMPASS-like” inclusion criteria |
| 16 | Wong KS, Chen C, Fu J, Chang HM, Suwanwela NC, Huang YN, Han Z, Tan KS, Ratanakorn D, Chollate P, et al. Clopidogrel plus aspirin versus aspirin alone for reducing embolisation in patients with acute symptomatic cerebral or carotid artery stenosis (CLAIR study): a randomised, open-label, blinded-endpoint trial. *LANCET NEUROL.* 2010;9(5):489-497. | Failure to meet a “COMPASS-like” inclusion criteria |
| 17 | Rogers RK, Hiatt WR, Patel MR, Shishehbor MH, White R, Khan ND, Bhalla NP, Jones WS, Low WC. Ticagrelor in Peripheral Artery Disease Endovascular Revascularization (TI-PAD): Challenges in clinical trial execution. *VASC MED.* 2018;23(6):513-522. | Unrelated studies |
| 18 | Rosenson RS, Chen Q, Najera SD, Krishnan P, Lee ML, Cho DJ. Ticagrelor improves blood viscosity-dependent microcirculatory flow in patients with lower extremity arterial disease: the Hema-kinesis clinical trial. *CARDIOVASC DIABETOL.* 2019;18(1):77. | Endpoint events of no interest |
| 19 | Hernandez-Suarez DF, Núñez-Medina H, Scott SA, Lopez-Candales A, Wiley JM, Garcia MJ, Melin K, Nieves-Borrero K, Rodriguez-Ruiz C, Marshall L, et al. Effect of cilostazol on platelet reactivity among patients with peripheral artery disease on clopidogrel therapy. *Drug Metab Pers Ther.* 2018;33(1):49-55. | Endpoint events of no interest |
| 20 | Miura T, Miyashita Y, Soga Y, Hozawa K, Doijiri T, Ikeda U, Kuwahara K. Drug-Eluting Versus Bare-Metal Stent Implantation With or Without Cilostazol in the Treatment of the Superficial Femoral Artery. *Circ Cardiovasc Interv.* 2018;11(8):e6564. | Failure to meet a “COMPASS-like” inclusion criteria |
| 21 | Ntalas IV, Kalantzi KI, Tsoumani ME, Bourdakis A, Charmpas C, Christogiannis Z, Dimoulis N, Draganigos A, Efthimiadis I, Giannakoulas G, et al. Salts of Clopidogrel: Investigation to Ensure Clinical Equivalence: A 12-Month Randomized Clinical Trial. *J Cardiovasc Pharmacol Ther.* 2016;21(6):516-525. | Antithrombotic regimens difficult to be included |
| 22 | Ntalas IV, Kalantzi KI, Tsoumani ME, Vakalis JN, Vasilakopoulos V, Vardakis K, Vemmos KN, Voukelatou M, Giannakoulas G, Giatrakos I, et al. Generic Clopidogrel Besylate in the Secondary Prevention of Atherothrombotic Events: A 6-month Follow-up of a Randomised Clinical Trial. *CURR VASC PHARMACOL.* 2015;13(6):809-818. | Failure to meet a “COMPASS-like” inclusion criteria |
| 23 | Tsoumani ME, Kalantzi KI, Dimitriou AA, Ntalas IV, Goudevenos IA, Tselepis AD. Antiplatelet efficacy of long-term treatment with clopidogrel besylate in patients with a history of acute coronary syndrome: comparison with clopidogrel hydrogen sulfate. *ANGIOLOGY.* 2012;63(7):547-551. | Failure to meet a “COMPASS-like” inclusion criteria |
| 24 | Soga Y, Takahara M, Iida O, Yamauchi Y, Hirano K, Fukunaga M, Zen K, Suzuki K, Shintani Y, Miyashita Y, et al. Efficacy of CilostAzol for Below-the-Knee Artery Disease after Balloon AnGioplasty in PatiEnts with Severe Limb Ischemia (CABBAGE Trial). *ANN VASC SURG.* 2017;45:22-28. | Failure to meet a “COMPASS-like” inclusion criteria |
| 25 | Kato T, Sakai H, Takagi T, Nishimura Y. Cilostazol prevents progression of asymptomatic carotid artery stenosis in patients with contralateral carotid artery stenting. *AJNR Am J Neuroradiol.* 2012;33(7):1262-1266. | Non randomized controlled trial |
| 26 | Huibers A, Halliday A, Bulbulia R, Coppi G, de Borst GJ. Antiplatelet Therapy in Carotid Artery Stenting and Carotid Endarterectomy in the Asymptomatic Carotid Surgery Trial-2. *Eur J Vasc Endovasc Surg.* 2016;51(3):336-342. | Unrelated studies |
| 27 | Yoshimoto T, Fujimoto S, Muraki M, Kobayashi R, Yoshidumi T, Yamauchi T, Tokuda K, Kaneko S. Cilostazol may suppress restenosis and new contralateral carotid artery stenosis after carotid endarterectomy. *Neurol Med Chir (Tokyo).* 2010;50(7):525-529. | Non randomized controlled trial |
| 28 | Soga Y, Hamasaki T, Edahiro R, Iida O, Inoue N, Suzuki K, Yokoi Y, Kawasaki D, Zen K, Urasawa K, et al. Sustained Effectiveness of Cilostazol After Endovascular Treatment of Femoropopliteal Lesions: Midterm Follow-up From the Sufficient Treatment of Peripheral Intervention by Cilostazol (STOP-IC) Study. *J ENDOVASC THER.* 2018;25(3):306-312. | Endpoint events of no interest |
| 29 | Iida O, Yokoi H, Soga Y, Inoue N, Suzuki K, Yokoi Y, Kawasaki D, Zen K, Urasawa K, Shintani Y, et al. Cilostazol reduces angiographic restenosis after endovascular therapy for femoropopliteal lesions in the Sufficient Treatment of Peripheral Intervention by Cilostazol study. *CIRCULATION.* 2013;127(23):2307-2315. | Failure to meet a “COMPASS-like” inclusion criteria |
| 30 | Liang GZ, Zhang FX, Luo XY, Zhang CM, Hu L, Feng YP, Niu LY, Zhang H, Ma BB, Qi HS, et al. [A prospective randomized control clinical trial about clopidogrel combined with warfarin versus clopidogrel alone in the prevention of restenosis after femoral-popliteal artery angioplasty]. *Zhonghua Wai Ke Za Zhi.* 2012;50(8):704-708. | Failure to meet a “COMPASS-like” inclusion criteria |
| 31 | Katakami N, Kim YS, Kawamori R, Yamasaki Y. The phosphodiesterase inhibitor cilostazol induces regression of carotid atherosclerosis in subjects with type 2 diabetes mellitus: principal results of the Diabetic Atherosclerosis Prevention by Cilostazol (DAPC) study: a randomized trial. *CIRCULATION.* 2010;121(23):2584-2591. | Failure to meet a “COMPASS-like” inclusion criteria |
| 32 | Zen K, Takahara M, Iida O, Soga Y, Kawasaki D, Nanto S, Yokoi H, Matoba S. Drug-eluting stenting for femoropopliteal lesions, followed by cilostazol treatment, reduces stent restenosis in patients with symptomatic peripheral artery disease. *J VASC SURG.* 2017;65(3):720-725. | Non randomized controlled trial |
| 33 | Lewis RJ, Connor JT, Teerlink JR, Murphy JR, Cooper LT, Hiatt WR, Brass EP. Application of adaptive design and decision making to a phase II trial of a phosphodiesterase inhibitor for the treatment of intermittent claudication. *TRIALS.* 2011;12:134. | Failure to meet a “COMPASS-like” inclusion criteria |
| 34 | Wang J, Zhu YQ, Li MH, Zhao JG, Tan HQ, Wang JB, Liu F, Cheng YS. Batroxobin plus aspirin reduces restenosis after angioplasty for arterial occlusive disease in diabetic patients with lower-limb ischemia. *J VASC INTERV RADIOL.* 2011;22(7):987-994. | Failure to meet a “COMPASS-like” inclusion criteria |
| 35 | Soga Y, Yokoi H, Kawasaki T, Nakashima H, Tsurugida M, Hikichi Y, Nobuyoshi M. Efficacy of cilostazol after endovascular therapy for femoropopliteal artery disease in patients with intermittent claudication. *J AM COLL CARDIOL.* 2009;53(1):48-53. | Failure to meet a “COMPASS-like” inclusion criteria |
| 36 | Hiatt WR, Money SR, Brass EP. Long-term safety of cilostazol in patients with peripheral artery disease: the CASTLE study (Cilostazol: A Study in Long-term Effects). *J VASC SURG.* 2008;47(2):330-336. | Failure to meet a “COMPASS-like” inclusion criteria |
| 37 | Catalano M, Born G, Peto R. Prevention of serious vascular events by aspirin amongst patients with peripheral arterial disease: randomized, double-blind trial. *J INTERN MED.* 2007;261(3):276-284. | Failure to meet a “COMPASS-like” inclusion criteria |
| 38 | Edmondson RA, Cohen AT, Das SK, Wagner MB, Kakkar VV. Low-molecular weight heparin versus aspirin and dipyridamole after femoropopliteal bypass grafting. *LANCET.* 1994;344(8927):914-918. | Endpoint events of no interest |
| 39 | Satiani B. A prospective randomized trial of aspirin in femoral popliteal and tibial bypass grafts. *ANGIOLOGY.* 1985;36(9):608-616. | Endpoint events of no interest |
| 40 | Dawson DL, Cutler BS, Meissner MH, Strandness DJ. Cilostazol has beneficial effects in treatment of intermittent claudication: results from a multicenter, randomized, prospective, double-blind trial. *CIRCULATION.* 1998;98(7):678-686. | Endpoint events of no interest |
| 41 | Ranke C, Creutzig A, Luska G, Wagner HH, Galanski M, Bode-Böger S, Frölich J, Avenarius HJ, Hecker H, ALexander K. Controlled trial of high- versus low-dose aspirin treatment after percutaneous transluminal angioplasty in patients with peripheral vascular disease. *Clin Investig.* 1994;72(9):673-680. | Endpoint events of no interest |
| 42 | Hobson RN, Krupski WC, Weiss DG. Influence of aspirin in the management of asymptomatic carotid artery stenosis. VA Cooperative Study Group on Asymptomatic Carotid Stenosis. *J VASC SURG.* 1993;17(2):257-263, 263-265. | Endpoint events of no interest |
| 43 | Leizorovicz A, Becker F. Oral buflomedil in the prevention of cardiovascular events in patients with peripheral arterial obstructive disease: a randomized, placebo-controlled, 4-year study. *CIRCULATION.* 2008;117(6):816-822. | Unrelated studies |
| 44 | Fiotti N, Altamura N, Cappelli C, Schillan M, Guarnieri G, Giansante C. Long term prognosis in patients with peripheral arterial disease treated with antiplatelet agents. *Eur J Vasc Endovasc Surg.* 2003;26(4):374-380. | Non randomized controlled trial |
| 45 | Fowkes FG, Price JF, Stewart MC, Butcher I, Leng GC, Pell AC, Sandercock PA, Fox KA, Lowe GD, Murray GD. Aspirin for prevention of cardiovascular events in a general population screened for a low ankle brachial index: a randomized controlled trial. *JAMA.* 2010;303(9):841-848. | Failure to meet a “COMPASS-like” inclusion criteria |
| 46 | Beebe HG, Dawson DL, Cutler BS, Herd JA, Strandness DJ, Bortey EB, Forbes WP. A new pharmacological treatment for intermittent claudication: results of a randomized, multicenter trial. *Arch Intern Med.* 1999;159(17):2041-2050. | Failure to meet a “COMPASS-like” inclusion criteria |
| 47 | Berent R, Auer J, Franklin B, Schmid P, von Duvillard SP. Platelet response to aspirin 50 and 100 mg in patients with coronary heart disease over a five-year period. *AM J CARDIOL.* 2011;108(5):644-650. | Endpoint events of no interest |
| 48 | Bergqvist D, Almgren B, Dickinson JP. Reduction of requirement for leg vascular surgery during long-term treatment of claudicant patients with ticlopidine: results from the Swedish Ticlopidine Multicentre Study (STIMS). *Eur J Vasc Endovasc Surg.* 1995;10(1):69-76. | Endpoint events of no interest |
| 49 | Burdess A, Nimmo AF, Garden OJ, Murie JA, Dawson AR, Fox KA, Newby DE. Randomized controlled trial of dual antiplatelet therapy in patients undergoing surgery for critical limb ischemia. *ANN SURG.* 2010;252(1):37-42. | Failure to meet a “COMPASS-like” inclusion criteria |
| 50 | Strobl FF, Brechtel K, Schmehl J, Zeller T, Reiser MF, Claussen CD, Tepe G. Twelve-month results of a randomized trial comparing mono with dual antiplatelet therapy in endovascularly treated patients with peripheral artery disease. *J ENDOVASC THER.* 2013;20(5):699-706. | Endpoint events of no interest |
| 51 | Arcan JC, Blanchard J, Boissel JP, Destors JM, Panak E. Multicenter double-blind study of ticlopidine in the treatment of intermittent claudication and the prevention of its complications. *ANGIOLOGY.* 1988;39(9):802-811. | Failure to meet a “COMPASS-like” inclusion criteria |
| 52 | Hess H, Mietaschk A, Deichsel G. Drug-induced inhibition of platelet function delays progression of peripheral occlusive arterial disease. A prospective double-blind arteriographically controlled trial. *LANCET.* 1985;1(8426):415-419. | Failure to meet a “COMPASS-like” inclusion criteria |
| 53 | Janzon L, Bergqvist D, Boberg J, Boberg M, Eriksson I, Lindgärde F, Persson G, Almgren B, Fagher B, Kjellström T, et al. Prevention of myocardial infarction and stroke in patients with intermittent claudication; effects of ticlopidine. Results from STIMS, the Swedish Ticlopidine Multicentre Study. *J INTERN MED.* 1990;227(5):301-308. | Failure to meet a “COMPASS-like” inclusion criteria |
| 54 | Colwell JA, Bingham SF, Abraira C, Anderson JW, Comstock JP, Kwaan HC, Nuttall F. V.A. Cooperative Study of antiplatelet agents in diabetic patients after amputation for gangrene: unobserved, sudden, and unexpected deaths. *J Diabet Complications.* 1989;3(4):191-197. | Failure to meet a “COMPASS-like” inclusion criteria |
| 55 | Shigematsu H, Komori K, Tanemoto K, Harada Y, Nakamura M. Clopidogrel for Atherothrombotic Event Management in Patients with Peripheral Arterial Disease (COOPER) Study: Safety and Efficacy of Clopidogrel versus Ticlopidine in Japanese Patients. *Ann Vasc Dis.* 2012;5(3):364-375. | Failure to meet a “COMPASS-like” inclusion criteria |
| 56 | Becquemin JP. Effect of ticlopidine on the long-term patency of saphenous-vein bypass grafts in the legs. Etude de la Ticlopidine après Pontage Fémoro-Poplité and the Association Universitaire de Recherche en Chirurgie. *N Engl J Med.* 1997;337(24):1726-1731. | Failure to meet a “COMPASS-like” inclusion criteria |
| 57 | McCollum C, Alexander C, Kenchington G, Franks PJ, Greenhalgh R. Antiplatelet drugs in femoropopliteal vein bypasses: a multicenter trial. *J VASC SURG.* 1991;13(1):150-161, 161-162. | Failure to meet a “COMPASS-like” inclusion criteria |
| 58 | Platelet inhibition with ASA/dipyridamole after percutaneous balloon angioplasty in patients with symptomatic lower limb arterial disease. A prospective double-blind trial. Study group on pharmacological treatment after PTA. *Eur J Vasc Surg.* 1994;8(1):83-88. | Endpoint events of no interest |
| 59 | Balsano F, Coccheri S, Libretti A, Nenci GG, Catalano M, Fortunato G, Grasselli S, Violi F, Hellemans H, Vanhove P. Ticlopidine in the treatment of intermittent claudication: a 21-month double-blind trial. *J Lab Clin Med.* 1989;114(1):84-91. | Endpoint events of no interest |
| 60 | Johnson WC, Williford WO. Benefits, morbidity, and mortality associated with long-term administration of oral anticoagulant therapy to patients with peripheral arterial bypass procedures: a prospective randomized study. *J VASC SURG.* 2002;35(3):413-421. | Endpoint events of no interest |
| 61 | Monaco M, Di Tommaso L, Pinna GB, Lillo S, Schiavone V, Stassano P. Combination therapy with warfarin plus clopidogrel improves outcomes in femoropopliteal bypass surgery patients. *J VASC SURG.* 2012;56(1):96-105. | Failure to meet a “COMPASS-like” inclusion criteria |
| 62 | Li H, Zhang F, Liang G, Luo X, Zhang C, Feng Y, Guo M. A prospective randomized controlled clinical trial on clopidogrel combined with warfarin versus clopidogrel alone in the prevention of restenosis after endovascular treatment of the femoropopliteal artery. *ANN VASC SURG.* 2013;27(5):627-633. | Failure to meet a “COMPASS-like” inclusion criteria |
| 63 | Patel MR, Becker RC, Wojdyla DM, Emanuelsson H, Hiatt WR, Horrow J, Husted S, Mahaffey KW, Steg PG, Storey RF, et al. Cardiovascular events in acute coronary syndrome patients with peripheral arterial disease treated with ticagrelor compared with clopidogrel: Data from the PLATO Trial. *EUR J PREV CARDIOL.* 2015;22(6):734-742. | Failure to meet a “COMPASS-like” inclusion criteria |
| 64 | Jones WS, Tricoci P, Huang Z, Moliterno DJ, Harrington RA, Sinnaeve PR, Strony J, Van de Werf F, White HD, Held C, et al. Vorapaxar in patients with peripheral artery disease and acute coronary syndrome: insights from Thrombin Receptor Antagonist for Clinical Event Reduction in Acute Coronary Syndrome (TRACER). *AM HEART J.* 2014;168(4):588-596. | Failure to meet a “COMPASS-like” inclusion criteria |
| 65 | Kawashima H, Tomaniak M, Ono M, Wang R, Hara H, Gao C, Takahashi K, Sharif F, Thury A, Suryapranata H, et al. Safety and Efficacy of 1-Month Dual Antiplatelet Therapy (Ticagrelor + Aspirin) Followed by 23-Month Ticagrelor Monotherapy in Patients Undergoing Staged Percutaneous Coronary Intervention (A Sub-Study from GLOBAL LEADERS). *AM J CARDIOL.* 2021;138:1-10. | Failure to meet a “COMPASS-like” inclusion criteria |
| 66 | Chichareon P, Modolo R, Kawashima H, Takahashi K, Kogame N, Chang CC, Tomaniak M, Ono M, Walsh S, Suryapranata H, et al. DAPT Score and the Impact of Ticagrelor Monotherapy During the Second Year After PCI. *JACC Cardiovasc Interv.* 2020;13(5):634-646. | Failure to meet a “COMPASS-like” inclusion criteria |
| 67 | Kulik A, Le May MR, Voisine P, Tardif JC, Delarochelliere R, Naidoo S, Wells GA, Mesana TG, Ruel M. Aspirin plus clopidogrel versus aspirin alone after coronary artery bypass grafting: the clopidogrel after surgery for coronary artery disease (CASCADE) Trial. *CIRCULATION.* 2010;122(25):2680-2687. | Failure to meet a “COMPASS-like” inclusion criteria |
| 68 | Ueda H, Kido A, Matsuhisa S, Asawa K, Yoshida N, Tsujimoto M, Sasaki Y, Kuga Y, Yamasaki M, Ueda K, et al. Addition of cilostazol to aspirin therapy for secondary prevention of cardiovascular and cerebrovascular disease in patients undergoing percutaneous coronary intervention: A randomized, open-label trial. *AM HEART J.* 2016;173:134-142. | Failure to meet a “COMPASS-like” inclusion criteria |
| 69 | Saw J, Wong GC, Mayo J, Bernstein V, Mancini GB, Ye J, Skarsgard P, Starovoytov A, Cairns J. Ticagrelor and aspirin for the prevention of cardiovascular events after coronary artery bypass graft surgery. *HEART.* 2016;102(10):763-769. | Failure to meet a “COMPASS-like” inclusion criteria |
| 70 | Hahn JY, Song YB, Oh JH, Chun WJ, Park YH, Jang WJ, Im ES, Jeong JO, Cho BR, Oh SK, et al. Effect of P2Y12 Inhibitor Monotherapy vs Dual Antiplatelet Therapy on Cardiovascular Events in Patients Undergoing Percutaneous Coronary Intervention: The SMART-CHOICE Randomized Clinical Trial. *JAMA.* 2019;321(24):2428-2437. | Failure to meet a “COMPASS-like” inclusion criteria |
| 71 | Watanabe H, Domei T, Morimoto T, Natsuaki M, Shiomi H, Toyota T, Ohya M, Suwa S, Takagi K, Nanasato M, et al. Effect of 1-Month Dual Antiplatelet Therapy Followed by Clopidogrel vs 12-Month Dual Antiplatelet Therapy on Cardiovascular and Bleeding Events in Patients Receiving PCI: The STOPDAPT-2 Randomized Clinical Trial. *JAMA.* 2019;321(24):2414-2427. | Failure to meet a “COMPASS-like” inclusion criteria |
| 72 | Koo BK, Kang J, Park KW, Rhee TM, Yang HM, Won KB, Rha SW, Bae JW, Lee NH, Hur SH, et al. Aspirin versus clopidogrel for chronic maintenance monotherapy after percutaneous coronary intervention (HOST-EXAM): an investigator-initiated, prospective, randomised, open-label, multicentre trial. *LANCET.* 2021;397(10293):2487-2496. | Failure to meet a “COMPASS-like” inclusion criteria |

## eFigure 1. Flow diagram of study search and selection.

2621 Records excluded (duplicated studies)

Primary analysis (7 trials)

## Included

## Eligibility

72 studies excluded:

48 Failure to meet inclusion criteria

15 Endpoint events of no interest

4 Non randomized controlled trial

3 Unrelated studies

2 Antithrombotic regimens difficult to be included

## Identification

5419 Records identified through database searching: 1725 records in Medline, 2312 records in EMBASE and 1382 records in Cochrane

2798 Records screened through titles or abstracts

83 Full-text articles assessed for eligibility

2716 Records excluded: Unrelated studies

## Screening

11 Trials included in the network meta-analysis

Sensitivity analysis (11 trials)

1 additional record identified by the update time (2021 July)

## eTable 3. Main characteristics of trials included in the meta-analysis

|  | Main characteristics of trials included in the meta-analysis | | | | | |
| --- | --- | --- | --- | --- | --- | --- |
| Administration method | Main inclusion criteria | Main exclusion criteria | Primary efficacy endpoints | Primary safety endpoints | Follow-up | Analysis |
| COMPASS: From 12, 2013 to 10, 2016; randomised, multicenter, double-blind, placebo-controlled trial | | | | | | |
| Patients were assigned to receive low-dose rivaroxaban (2.5 mg twice daily) plus aspirin (100 mg once daily) or rivaroxaban alone (5 mg twice daily), or aspirin alone (100 mg once daily) | 1. Patients had either myocardial infarction within 20 years, or multivessel coronary disease with symptoms or with history of stable or unstable angina, or previous multi-vessel PCI, or previous multi-vessel CABG.   OR   1. Patients had either previous limb or carotid revascularization, previous limb or foot amputation, or history of intermittent claudication with an ABI < 0.9 or significant stenosis (≥50%) of peripheral arteries. | 1. The use of dual antiplatelet therapy, anticoagulation, or other antithrombotic therapy. 2. Patients had stroke within 1 month or any history of hemorrhagic or lacunar stroke. 3. High risk of bleeding | Composite of cardiovascular death, stroke, or myocardial infarction | A modification of the ISTH criteria for major bleeding and included fatal bleeding, symptomatic bleeding into a critical organ, bleeding into a surgical site requiring reoperation, and bleeding that led to hospitalization (including presentation to an acute care facility without an overnight stay) | 23-month | The efficacy and safety outcomes were conducted on an ITT basis. |
| VOYAGER PAD: From 8, 2015 to 1, 2018; randomised, multicenter, double-blind, placebo-controlled trial | | | | | | |
| Patients were assigned to receive low-dose rivaroxaban (2.5 mg twice daily) or placebo.  All patients were to receive aspirin  at a dose of 100 mg daily as background therapy. 51% took clopidogrel and the median duration of clopidogrel was 29 days. | Patients had documented lower-extremity peripheral artery disease, including symptoms, anatomical evidence, and hemodynamic evidence, with an ABI ≤ 0.80 or TBI ≤ 0.60 for patients without a prior history of limb revascularization, or with an ABI ≤ 0.85 or TBI ≤ 0.65 for patients with a prior history of limb revascularization | 1. Patients were taking or were anticipated to begin taking prohibited concomitant medications, including long-term treatment with clopidogrel. 2. Planned dual antiplatelet therapy use for the qualifying revascularization procedure of clopidogrel in addition to aspirin for >6 months after the qualifying revascularization procedure; it is strongly recommended that any course of clopidogrel is kept to the minimum necessary in accordance with local standard of care and international practice guidelines. 3. Patients had any documented history of intracranial hemorrhage, stroke, or transient ischemic attack. 4. High risk of bleeding | Composite of acute limb ischemia, major amputation for vascular causes, myocardial infarction, ischemic stroke, or death from cardiovascular causes | Major bleeding defined according to the TIMI classification | 28-month | 1. The efficacy outcomes were conducted on an ITT basis.  2. The safety outcomes were conducted on an on-treatment basis, which included all patients who underwent randomization and received at least one dose of trial medication.  3. Clopidogrel was used by half of the patients in the rivaroxaban plus aspirin and aspirin arms in the original study, which may have significantly biased the primary outcomes, so data from the non clopidogrel subgroup were selected for this meta-analysis, while data from the whole study were simultaneously included in a sensitivity analysis. |
| COMMANDER HF: From 9, 2013 to 10, 2017; randomised, multicenter, double-blind, placebo-controlled trial; only in a sensitivity analysis | | | | | | |
| Patients were assigned to receive 2.5 mg of rivaroxaban twice daily or matching placebo. In addition, aspirin, alone or in combination with a thienopyridine, was taken by 93.1% of the patients, with 34.8% taking dual antiplatelet therapy at baseline. | Patients who had at least a 3-month history of chronic heart failure, a left ventricular ejection fraction of 40% or less, and coronary artery disease and who had been treated for an episode of worsening heart failure. In addition, more than 75% of the patients actually included had a history of myocardial infarction. | 1. Atrial fibrillation or another condition that required long-term anticoagulation. 2. Acute myocardial infarction or surgical or percutaneous coronary artery intervention during the index event 3. High risk of bleeding | Composite of death from any cause, myocardial infarction, or stroke | The principal safety outcome was the composite of fatal bleeding or bleeding into a critical space with a potential for causing permanent disability. However, there was no safety outcome in the subgroup. | 21.1-month | 1. The efficacy outcomes were conducted on an ITT basis.  2. The safety outcomes werewere restricted to patients who took at least one dose of rivaroxaban or placebo.  3. In addition to the inclusion of those intervention drugs in COMMANDER HF, thienopyridines were allowed to exist as needed on an actual basis. However, clear groupings were performed in subgroup analyses of the effect outcomes, including aspirin, aspirin plus rivaroxaban, clopidogrel, clopidogrel plus rivaroxaban, and aspirin plus clopidogrel plus rivaroxaban. Consequently, COMMANDER HF was included for sensitivity analysis only. |
| PEGASUS-TIMI 54: From 10, 2010 to 5, 2013; randomised, multicenter, double-blind, placebo-controlled trial | | | | | | |
| Patients were assigned receive ticagrelor orally at a dose of 90 mg twice daily, ticagrelor orally at a dose of 60 mg twice daily, or placebo. And all the patients also received aspirin (75 to 150 mg once daily). | Patients had documented history of presumed spontaneous MI (excluding known peri-procedural or definite secondary MI [eg, due to profound hypotension, hypertensive emergency, tachycardia, or profound anemia]) with their most recent MI occurring 1 to 3 years prior to randomization. | 1. Patients were ineligible if there was planned use of a P2Y12 receptor antagonist, dipyridamole, cilostazol, or anticoagulant therapy during the study period. 2. Patients had history of ischemic stroke at any time. 3. High risk of bleeding | Composite of cardiovascular death, myocardial infarction, or stroke | Major bleeding defined according to the TIMI classification | 33-month | 1. The efficacy outcomes were conducted on an ITT basis.  2. The safety analyses included all the patients who underwent randomization and received at least one dose of study drug. |
| THEMIS: From 2, 2014 to 5, 2016; randomised, multicenter, double-blind, placebo-controlled trial | | | | | | |
| Patients were assigned to receive ticagrelor at a dose of 90 mg twice daily and later changed to 60 mg twice daily, or placebo alone. And all the patients also received aspirin (75 to 150 mg once daily). | Patients had a known history of at least one vessel stenosis ≥ 50% after PCI or CABG or angiography, and a history of type 2 diabetes mellitus. | 1. Patients originally planned to receive dual antiplatelet therapy or anticoagulant therapy. 2. Patients had previous spontaneous MI except for definite secondary MI (e.g., due to coronary revascularization procedure, profound hypotension, hypertensive emergency, tachycardia, or profound anemia). 3. Patients had previous stroke (TIA is not included in the stroke definition). 4. High risk of bleeding | Composite of cardiovascular death, myocardial infarction, or stroke | Major bleeding defined according to the TIMI classification | 39.9-month | 1. The efficacy outcomes were conducted on an ITT basis. 2. The safety analyses were performed in the safety analysis set, which included all the patients who had received at least one dose of ticagrelor or placebo, with patients evaluated according to the one they actually received. 3. Data from the subgroup with multivessel coronary disease were included according to the inclusion criteria of the present meta-analysis, whereas data from the whole study were included in a sensitivity analysis. |
| DAPT: From 8, 2009 to 7, 2011; randomised, multicenter, double-blind, placebo-controlled trial | | | | | | |
| Patients were assigned receive clopidogrel at a maintenance dose of 75 mg daily or prasugrel at a maintenance dose of 10 mg daily (with a dose of 5 mg daily or placebo. And all the patients also received aspirin (75 to 162 mg once daily). | Patients had undergoing PCI with stent deployment for 12 months and there was no MACEs and major bleeding during this period. | 1. Patients originally planned to receive dual antiplatelet therapy or anticoagulant therapy. 2. High risk of bleeding | Definite or probable stent thrombosis (as assessed according to the Academic Research Consortium definitions) and of MACEs (defined as the composite of death, myocardial infarction, or stroke). | Moderate or severe bleeding during this same period (as assessed according to the GUSTO criteria | 18-month | 1. The efficacy outcomes were conducted on an ITT basis. 2. The safety analyses was performed on data from patients who underwent randomization, were treated with drugeluting stents, and completed at least 17 months of follow-up (the minimum window allowed for the 18-month postrandomization visit) or had a moderate or severe bleeding event. 3. The intervention drugs included clopidogrel (65%) and prasugrel (35%), in which the proportion of clopidogrel was high among DAPT and the other thienopyridines in this meta-analysis were all clopidogrel, so clopidogrel was represented in this analysis. 4. Data from the subgroup with MI were included according to the inclusion criteria of the present meta-analysis, whereas data from the whole study were included in a sensitivity analysis. |
| CHARISMA: From 10, 2002 to 11, 2003; randomised, multicenter, double-blind, placebo-controlled trial; only in a sensitivity analysis | | | | | | |
| Patients were assigned receive clopidogrel at a maintenance dose of 75 mg daily or placebo. And all the patients also received aspirin (75 to 162 mg once daily). | Patients had one of the following conditions: multiple atherothrombotic risk factors, documented coronary disease, documented cerebrovascular disease, or documented symptomatic peripheral arterial disease. | 1. Patients were also excluded if, in the judgment of the investigator, they had established indications for clopidogrel therapy (such as a recent acute coronary syndrome). Patients who were scheduled to undergo a revascularization were not allowed to enroll until the procedure had been completed; such patients were excluded if they were considered to require clopidogrel after revascularization. 2. High risk of bleeding | Composite of myocardial infarction, stroke (of any cause), or death from cardiovascular causes (including hemorrhage) | Severe bleeding defined according to the GUSTO criteria | 28-month | 1. The efficacy and safety outcomes were conducted on an ITT basis. 2. Thirty five percent of patients with a history of stroke or TIA were included in CHARISMA, and antithrombotic regimens in these patients have been generally considered recently to have significant clinical heterogeneity with those of two other antithrombotic regimens for ASCVD, so data from CHARISMA were included for sensitivity analysis only. |
| EUCLID: From 10, 2012 to 3, 2014; randomised, multicenter, double-blind, placebo-controlled trial | | | | | | |
| Patients were assigned receive ticagrelor (90 mg twice daily) or clopidogrel (75 mg once daily). | Patients had previous revascularization of the lower limbs for symptomatic disease more than 30 days before randomization, or had hemodynamic evidence of peripheral artery disease, as evidenced by an ABI ≤ 0.80 or TBI ≤ 0.60. | 1. Patients originally planned to receive dual antiplatelet therapy or anticoagulant therapy. 2. High risk of bleeding | Composite of cardiovascular death, myocardial infarction, or ischemic stroke (defined as any stroke not shown to be primarily hemorrhagic) | Major bleeding defined according to the TIMI classification | 30-month | 1. The efficacy outcomes were conducted on an ITT basis. 2. The safety analyses included all the patients who received at least one dose of a trial drug during the treatment period, which was defined as up to 7 days after the last dose of a trial drug. |
| TWILIGHT COMPLEX: From 7, 2015 to 10, 2017; randomised, multicenter, double-blind, placebo-controlled trial | | | | | | |
| Patients were assigned receive aspirin at a maintenance dose of 81 to 100 mg daily or placebo. And all the patients also received ticagrelor (90 mg twice daily). | Patients who underwent successful PCI with at least one locally approved drug-eluting stent and whom the treating clinician intended to discharge with a regimen of ticagrelor plus aspirin were eligible to participate. Patients also had to have at least one additional clinical feature and one angiographic feature associated with a high risk of ischemic or bleeding events. TWILIGHT COMPLEX included patients with complex PCI, and 74% of them had multi-vessel lesions. | 1. After receiving dual antiplatelet therapy for 3 months post PCI, patients were still planed to require dual antiplatelet therapy. 2. Patients were receiving or were originally needed to receive anticoagulant therapy. 3. Salvage PCI for cardiogenic shock or STEMI presentation 4. Prior stroke 5. High risk of bleeding | Composite of death from any cause, nonfatal myocardial infarction, or nonfatal stroke | 1. BARC type 2, 3, or 5 bleeding 2. The secondary outcomes included BARC type 3 or 5 bleeding, which served as the primary outcomes in this meta-analysis. | 12-month | 1. The efficacy outcomes were conducted on an per-protocol populations basis. Patients who underwent randomization and did not fulfill enrollment criteria, were not eligible for randomization, or never received protocol-mandated therapy were excluded from the per-protocol analysis. 2. The safety outcomes were conducted on an ITT basis. 3. Data from the subgroup with complex PCI were included according to the inclusion criteria of the present meta-analysis, whereas data from the whole study were included in a sensitivity analysis. |
| CAPRIE: From 3, 1992 to 2, 1995; randomised, multicenter, blind, placebo-controlled trial; only in a sensitivity analysis | | | | | | |
| Patients were assigned to receive 75 mg tablets of clopidogrel plus aspirin placebo or 325 mg tablets of aspirin plus clopidogrel placebo. | 1. Patients had one of the following conditions: ischaemic stroke, MI, or PAD. 2. Patients with PAD had one of the following conditions: previous leg amputation, or previous revascularization, or history of intermittent claudication with an ABI ≤ 0.85. | 1. Anticipated requirement for long-term anticoagulants, non-study antiplatelet drugs or non-steroidal anti-inflammatory drugs affecting platelet function. 2. High risk of bleeding | Composite of ischaemic stroke, myocardial infarction, or vascular death | None | 23-month | 1. The efficacy outcomes were conducted on an ITT basis. 2. Safety assessments were based on the proportion of patients experiencing one or more episodes of a specific adverse event. 3. The PAD subgroup in the CAPRIE met the inclusion criteria for this meta-analysis, but because there was no outcome of major bleeding (an analysis of net clinical benefit would be biased), data on PAD were only included as a sensitivity analysis. |
| DAVID: From 2, 1996 to 10, 1998; randomised, multicenter, blind, placebo-controlled trial; only in a sensitivity analysis | | | | | | |
| Patients were assigned to receive one picotamide 600 mg tablet twice daily or one aspirin 320 mg tablet once daily in the morning plus one placebo tablet once daily in the evening. | Patients with a history of type 2 diabetes for 5 years or more and PAD were eligible for inclusion in the study. PAD was defined as the presence of two or more of the following: (1) history of intermittent; (2) loss of posterior tibial pulse in the foot; (3) ABI <0.90; (4) amputation or reconstructive surgery in patients with previous history of intermittent claudication; (5) angioplasty with no persisting complication from intervention. | 1. Myocardial infarction, stroke or unstangina in the 6 months prior to enrolment. 2. High risk of bleeding | 1. The primary endpoint was the overall mortality. 2. The secondary outcomes included the occurrence of MACEs (defined as the composite of amputation. death, myocardial infarction, or stroke) | None | 24-month | 1. The outcomes were conducted on an ITT basis. |

ABI: ankle brachial index, ASCVD: arteriosclerotic cardiovascular disease, BARC: Bleeding Academic Research Consortium, CABG: coronary-artery bypass grafting, GUSTO: Global Utilization of Streptokinase and Tissue Plasminogen Activator for Occluded Arteries, ITT: intention-to-treat, ISTH: International Society on Thrombosis and Haemostasis, MACEs: major adverse cardiovascular and cerebrovascular events, MI: myocardial infarction, TBI: toe-brachial index, TIMI: Thrombolysis in Myocardial Infarction, TIA: transient ischemic attack

## eTable 4. Main characteristics of patients enrolled among trials included in the meta-analysis

|  | Treatment Regimen | | | | | | | | | | |
| --- | --- | --- | --- | --- | --- | --- | --- | --- | --- | --- | --- |
| Characteristic | Rivaroxaban + Aspirin VS Aspirin | | | Ticagrelor +Aspirin VS Aspirin | | Clopidogrel +Aspirin VS Aspirin | | Ticagrelor VS Clopidogrel | Ticagrelor VS Ticagrelor + Aspirin | Clopidogrel VS Aspirin | Picotamide VS Aspirin |
| Study name | COMPASS^*^ | VOYAGER PAD^†^ | COMMANDER HF^*‡^ | PEGASUS-TIMI 54 | THEMIS^†^ | DAPT^†^ | CHARISMA^‡^ | EUCLID | TWILIGHT COMPLEX^†^ | CAPRIE^‡^ | DAVID^‡^ |
| No. of participants in NMA | 27395 | 3234 | 4944 | 21162 | 11935 | 3576 | 15603 | 13885 | 2342 | 6452 | 1209 |
| Age, mean ±SD, years | 68.2±7.9 | 67.0±8.9 | 66.4±10.2 | 65.3±8.4 | 66.0±8.1 | 57.8±10.5 | 64.0±13.0 | 66±9.3 | 66.0±10.4 | 64.3±9.6 | 64.2±7.3 |
| Female sex, % | 22.0 | 23.7 | 22.1 | 23.9 | 31.4 | 21.8 | 29.8 | 28.0 | 21.3 | 27.5 | 27.4 |
| BMI, mean ±SD, kg/m² | 28.3±4.7 | 25.7±5.6 | 27.7±5.2 |  | 29.0±4.9 | 29.9±5.5 |  |  | 28.1±5.3 |  | 28.4±5.4 |
| Current smoker, % | 21.4 | 35.2 |  | 16.7 | 10.9 | 41.8 | 20.2 | 28.0 | 20.6 | 38.0 | 29.4 |
| White race, % | 62.5 | 82.1 | 82.2 | 86.6 | 71.3 | 91.6 | 80.1 |  | 65.7 | 98.0 |  |
| Hypertension, % | 75.3 | 80.3 | 75.3 | 77.5 | 92.5 | 58.1 | 73.6 | 78.2 | 71.2 | 51.0 | 56.9 |
| Dyslipidemia, % |  | 54.6 |  | 76.7 | 87.2 |  | 73.9 | 75.5 | 58.2 | 45.0 | 38.2 |
| Diabetes, % | 37.8 | 34.1 | 40.8 | 32.2 | 100.0 | 20.9 | 42.0 | 38.5 | 37.0 | 21.0 | 100.0 |
| Previous stroke, % | 3.8 | 0 | 9.0 | 0 | 0 | 2.1 | 24.6 | 8.2 | 0 | 6 | 10.3 |
| History of atherosclerosis |  |  |  |  |  |  |  |  |  |  |  |
| CAD, % | 90.6 | 28.9 | 100.0 | 100.0 | 100.0 | 100.0 | 37.4 | 29.0 | 100.0 |  | 19.2 |
| MCAD, % | 56.5 |  |  | 59.2 | 100.0 |  | 11.4 |  | 74.0 |  |  |
| Previous MI, % | 62.2 | 10.7 | 75.7 | 100 | 0 | 100.0 | 34.6 | 18.2 | 28.7 | 21.0 |  |
| Previous PCI, % | 54.3 | 10.1 |  | 83.0 | 58.0 | 100.0 | 22.8 | 15.6 | 41.5 |  |  |
| Previous CABG, % | 28.6 | 7.4 |  |  | 28.8 | 4.8 | 19.7 | 11.1 | 15.4 |  |  |
| PAD characteristics |  |  |  |  |  |  |  |  |  |  |  |
| PAD, % | 27.3 | 100.0 |  | 5.4 | 8.8 |  | 22.6 | 100 | 7.9 | 100.0 | 100.0 |
| Previous limb revascularization, % | 7.5 | 31.2 |  |  |  |  | 11.1 | 56.7 |  |  |  |
| Previous limb or foot amputation, % | 1.2 | 6.0 |  |  |  |  |  | 6.6 |  |  |  |

BMI: body mass index, CABG: coronary-artery bypass grafting, CAD: coronary artery disease, MCAD: multivessel coronary artery disease, MI: myocardial infarction, NMA: network meta analysis; PAD: peripheral arterial disease, PCI: percutaneous coronary intervention, SD: standard deviation.

^*^ COMPASS is a three arm study that included rivaroxaban, aspirin, and rivaroxaban plus aspirin. The values in this table provide the results for these three groups combined. COMMANDER HF is a two arm study, but according to the purpose of this meta-analysis and combined with the subgroup data of COMMANDER HF, it can be divided into four arms, including aspirin, aspirin plus rivaroxaban, clopidogrel, clopidogrel plus rivaroxaban, and aspirin plus clopidogrel and rivaroxaban. The sample size of aspirin plus rivaroxaban versus aspirin accounted for 60% of the total sample size. In addition, due to the lack of baseline data for subgroups, the data in this table are from the whole study.

^†^ The present meta-analysis was performed on the subgroup data of four studies VOYAGER PAD, THEMIS, DAPT and TWILIGHT COMPLEX according to the inclusion criteria, but in which the subgroup of THEMIS had missing baseline data, so the baseline data of the whole study was used instead.

‡ COMMANDER HF, CHARISMA, CAPRIE and DAVID do not completely meet the inclusion criteria but are among the well-designed large-scale randomized controlled trials and are therefore included in sensitivity analyses.

## eFigure 2. Risk of bias of included trials using the Cochrane risk assessment tool


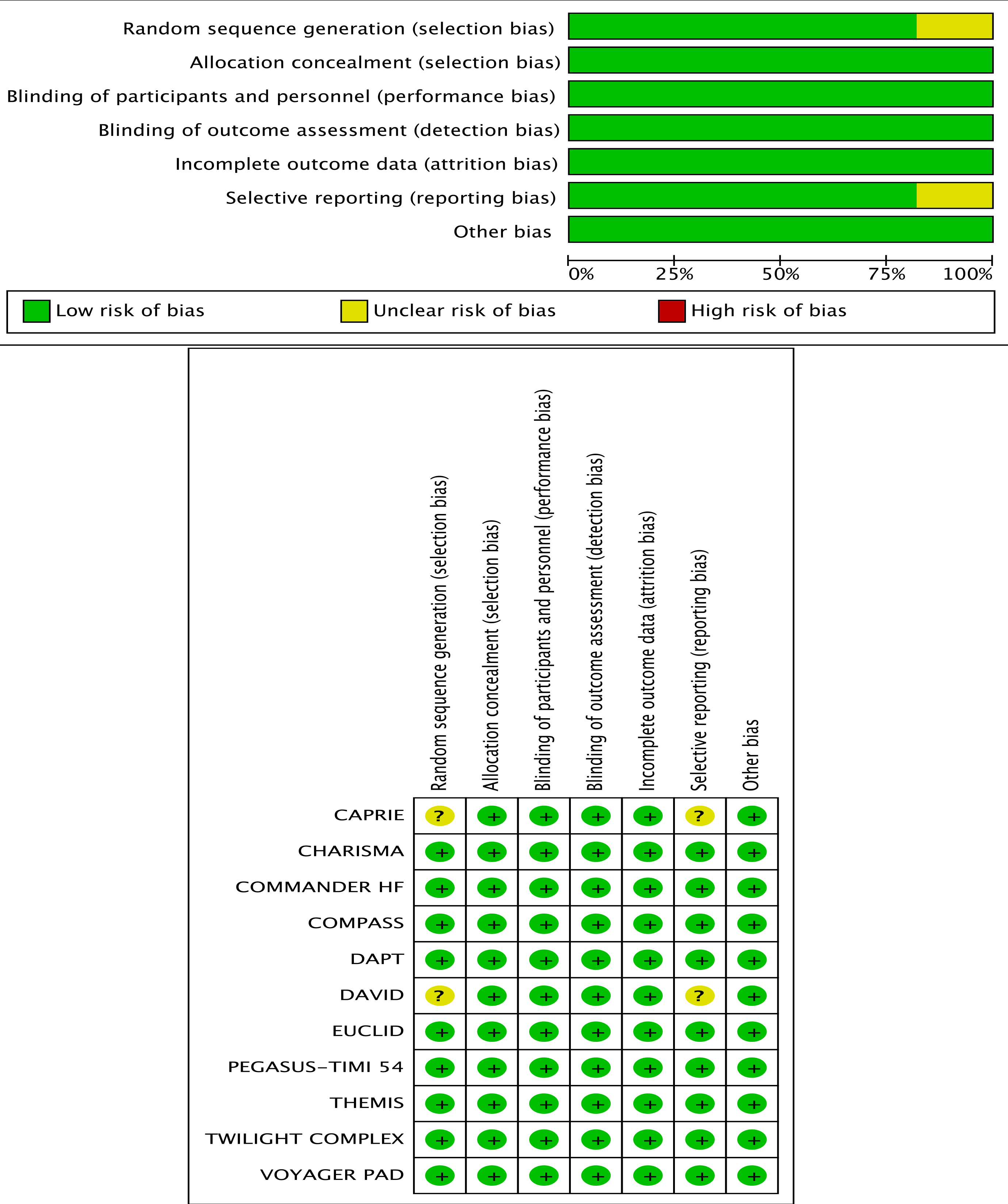


## eTable 5. Evaluation of risk of bias of included trials

|  | Risk bias assessment | | | | | | |
| --- | --- | --- | --- | --- | --- | --- | --- |
| Random sequence generation | | Allocation concealment | Blinding of participants &personnel | Blinding of outcome assessment | Incomplete outcome data | Selective reporting | Other bias (Status of pharmaceutical sponsor) |
| COMPASS | | | | | | | |
| A computer-generated randomisation schedule was generated by the Population Health Research Institute and used to allocate participants to treatment.  Authors' judgement: Low risks | A central internet web-based randomisation was used for the allocation of participants to receive one of the three antithrombotic therapy treatments in a double-blind manner.  Authors' judgement: Low risks | | Each treatment group was double dummy, and the patients, investigators, and central study staff were masked to treatment allocation. And because the endpoint events are objective events, there is no subjective evaluation, so the blind method will not be destroyed.  Authors' judgement: Low risks | Each treatment group was double dummy, and the patients, investigators, and central study staff were masked to treatment allocation. And because the endpoint events are objective events, there is no subjective evaluation, so the blind method will not be destroyed.  Authors' judgement: Low risks | Data regarding vital status were available or 27,331 participants (99.8%).  Authors' judgement: Low risks | It was consistent with the outcomes of the protocol.  Authors' judgement: Low risks | The study was designed by the Steering Committee, which included scientists from the sponsor, Bayer AG, who collaborated in study design, manuscript review and decision to publish. Site management and data collection and analysis were done at the Population Health Research Institute, Hamilton Health Sciences, and McMaster University in Hamilton, ON, Canada.  Authors' judgement: Low risks |
| VOYAGER PAD | | | | | | | |
| Randomization was performed with a centralized computerized system.  Authors' judgement: Low risks | Randomization was performed with a centralized computerized system, which belonged to the central allocation.  Authors' judgement: Low risks | | The trial-group assignment was conducted in a double blind manner.  And because the endpoint events are objective events, there is no subjective evaluation, so the blind method will not be destroyed.  Authors' judgement: Low risks | An independent academic clinical events committee adjudicated all deaths, potential ischemic cardiovascular and limb events, and bleeding events in a blinded manner.  Authors' judgement: Low risks | At the end of the trial, data on vital status were available for 6538 patients (99.6%) and  were missing for 8 patients and 12 patients in the rivaroxaban and placebo groups, respectively, who had withdrawn consent, as well as 3 patients in each group who were lost to follow-up.  Authors' judgement: Low risks | It was consistent with the outcomes of the protocol.  Authors' judgement: Low risks | It was designed and overseen by a collaborative group that included Colorado Prevention Center (CPC) Clinical Research (an academic research organization affiliated with the University of Colorado), the academic executive committee, and the sponsors, Bayer and Janssen Pharmaceuticals. Bayer participated in the trial design, trial oversight, site selection, and the drafting of the manuscript. A contract research organization (Covance) was responsible for site selection, data storage, and data monitoring. The CPC and the executive committee, which included employees of the sponsors, were responsible for trial design and oversight, data interpretation, and the drafting of the manuscript and the decision to submit the manuscript for publication.  Authors' judgement: Low risks |
| COMMANDER HF; only in a sensitivity analysis | | | | | | | |
| An interactive Web response system was used.  Authors' judgement: Low risks | Using an interactive Web response system and  permuted blocks of four, we randomly assigned  patients in a 1:1 ratio to receive 2.5 mg of rivaroxaban twice daily or matching placebo, within  strata defined according to country.  Authors' judgement: Low risks | | The trial-group assignment was conducted in a double blind manner.  And because the endpoint events are objective events, there is no subjective evaluation, so the blind method will not be destroyed.  Authors' judgement: Low risks | An independent data and safety monitoring committee had complete access to unblinded data during the conduct of the trial and were responsible for the safety of the enrolled patients as well as for the performance of a single, prespecified interim analysis for efficacy. And because the endpoint events are objective events, there is no subjective evaluation, so the blind method will not be destroyed.  Authors' judgement: Low risks | On the global treatment end date, data on vital status were available for 5013 patients (99.8%).  Authors' judgement: Low risks | It was consistent with the outcomes of the protocol.  Authors' judgement: Low risks | An international steering committee, made up of members from academic institutions and one member from Janssen, designed the trial, were responsible for overseeing the conduct of the trial, retained the ability to present the data, and made the decision to submit the manuscript for publication.  Authors' judgement: Low risks |
| PEGASUS-TIMI 54 | | | | | | | |
| Randomization was performed with the use of a central computerized telephone or Web-based system.  Authors' judgement: Low risks | Randomization was performed with the use of a central computerized telephone or Web-based system.  Authors' judgement: Low risks | | Assignment was double-blinded. And because the endpoint events are objective events, there is no subjective evaluation, so the blind method will not be destroyed.  Authors' judgement: Low risks | A central clinical-events committee, whose members were unaware of the treatment assignments, adjudicated all efficacy end points and bleeding episodes. And because the endpoint events are objective events, there is no subjective evaluation, so the blind method will not be destroyed.  Authors' judgement: Low risks | Ascertainment of the primary end point was complete for 99.2% of the potential patient-years of follow-up.  Authors' judgement: Low risks | It was consistent with the outcomes of the protocol.  Authors' judgement: Low risks | The raw database was provided to the TIMI Study Group, which conducted all the data analyses independently of the sponsor.  Authors' judgement: Low risks |
| THEMIS | | | | | | | |
| Randomization codes were generated in blocks of constant size.  Authors' judgement: Low risks | Eligible patients were randomly assigned in a 1:1 ratio to the ticagrelor group or the placebo group by means of an interactive voice-response or Web-response system. Randomization codes were generated in blocks of constant size.  Authors' judgement: Low risks | | The trial-group assignment was conducted in a double blind manner. And because the endpoint events are objective events, there is no subjective evaluation, so the blind method will not be destroyed.  Authors' judgement: Low risks | An academic clinical events committee adjudicated endpoint events in a blinded manner. And because the endpoint events are objective events, there is no subjective evaluation, so the blind method will not be destroyed.  Authors' judgement: Low risks | Data regarding vital status were available for 99.9% of the patients at the end of the trial and were missing for 21 patients (13 in the ticagrelor group and 8 in the placebo group); of these patients, 10 were lost to follow-up, and 11 withdrew consent and had unknown vital status.  Authors' judgement: Low risks | It was consistent with the outcomes of the protocol.  Authors' judgement: Low risks | Site selection was conducted jointly by the national lead investigators and representatives of AstraZeneca, who performed site monitoring and supervision and handled the collection, storage, and analysis of the data. The Baim Clinical Research Institute independently validated all the data that are reported, with funding from AstraZeneca.  Authors' judgement: Low risks |
| DAPT | | | | | | | |
| A computer-generated randomization schedule stratified patients according to the type of stent they had received (drug-eluting vs. bare-metal), hospital site, type of thienopyridine drug, and presence or absence of at least one prespecified clinical or lesion-related risk factor for stent thrombosis.  Authors' judgement: Low risks | Randomization was performed by a central Interactive Voice Response System (IVRS) for all studies, except the Boston Scientific Liberté study, which used its own IVRS system.  Authors' judgement: Low risks | | Assignment was double-blinded. And because the endpoint events are objective events, there is no subjective evaluation, so the blind method will not be destroyed.  Authors' judgement: Low risks | A single clinical-events committee whose members were unaware of the group assignments adjudicated events, and an unblinded, independent, central data and safety monitoring committee oversaw the safety of all patients. And because the endpoint events are objective events, there is no subjective evaluation, so the blind method will not be destroyed.  Authors' judgement: Low risks | 94.3% of the participants completed the follow-up  Authors' judgement: Low risks | It was consistent with the outcomes of the protocol.  Authors' judgement: Low risks | The stent manufacturers who funded the trial had contributing roles in the design of the trial and in the collection of the data. The Harvard Clinical Research Institute was responsible for the scientific conduct of the trial and an independent analysis of the data.  Authors' judgement: Low risks |
| CHARISMA; only in a sensitivity analysis | | | | | | | |
| Study-drug assignment was performed centrally by an interactive voice-response system on the basis of a preestablished randomization scheme, stratified according to site.  Authors' judgement: Low risks | Study-drug assignment was performed centrally by an interactive voice-response system.  Authors' judgement: Low risks | | Assignment was double-blinded. And because the endpoint events are objective events, there is no subjective evaluation, so the blind method will not be destroyed.  Authors' judgement: Low risks | The locked, cleaned database was transferred to the Cleveland Clinic Cardiovascular Coordinating Center, where data analysis was performed. And because the endpoint events are objective events, there is no subjective evaluation, so the blind method will not be destroyed.  Authors' judgement: Low risks | Follow-up with respect to the primary efficacy end point was complete in 99.5 percent of the patients randomly assigned to receive clopidogrel and aspirin and 99.6 percent of those randomly assigned to receive placebo and aspirin.  Authors' judgement: Low risks | It was consistent with the outcomes of the protocol.  Authors' judgement: Low risks | Funding for the CHARISMA trial was provided by Sanofi-Aventis and Bristol-Myers Squibb. The sponsor and cosponsor had advisory input in the design of the study, had nonvoting input in the executive committee, and were responsible for auditing at individual study sites. The executive committee bears complete responsibility for the analysis of the results, the veracity and completeness of the reporting, and the writing of the manuscript; the sponsors did have the opportunity to review the manuscript.  Authors' judgement: Low risks |
| EUCLID | | | | | | | |
| Randomization was performed with the use of an interactive voice-response or Web-response system.  Authors' judgement: Low risks | Randomization was performed with the use of an interactive voice-response or Web-response system.  Authors' judgement: Low risks | | Assignment was double-blinded. And because the endpoint events are objective events, there is no subjective evaluation, so the blind method will not be destroyed.  Authors' judgement: Low risks | All primary efficacy and safety end points were adjudicated by an independent clinical events committee in a blinded fashion. An independent data and safety monitoring committee provided safety oversight and performed one formal interim analysis after 798 primary events had been adjudicated. And because the endpoint events are objective events, there is no subjective evaluation, so the blind method will not be destroyed.  Authors' judgement: Low risks | At the completion of the trial, vital status was unknown for 14 patients (1%); of these patients, 5 were lost to follow-up.  Authors' judgement: Low risks | It was consistent with the outcomes of the protocol.  Authors' judgement: Low risks | AstraZeneca provided financial support for the conduct of the study. The Duke Clinical Research Institute held the clinical database and conducted all analyses for publication independent of the sponsor.  Authors' judgement: Low risks |
| TWILIGHT COMPLEX | | | | | | | |
| Randomization was performed with a secure Web-based system; an independent statistician who was not involved with the trial generated the randomization sequence, which was stratified according to site with randomly varying block sizes of 4, 6, and 8.  Authors' judgement: Low risks | Randomization was performed with a secure Web-based system.  Authors' judgement: Low risks | | Assignment was double-blinded. And because the endpoint events are objective events, there is no subjective evaluation, so the blind method will not be destroyed.  Authors' judgement: Low risks | The executive and steering committees were responsible for trial conduct, the integrity of the data analysis, and the reporting of results. And because the endpoint events are objective events, there is no subjective evaluation, so the blind method will not be destroyed.  Authors' judgement: Low risks | Ascertainment of the primary end point was complete in 98.4% of the patients who underwent randomization, and data on vital status were obtained in 99.7%.  Authors' judgement: Low risks | It was consistent with the outcomes of the protocol.  Authors' judgement: Low risks | AstraZeneca provided financial support and supplied ticagrelor for the trial but had no role in the design, collection, analysis, or  interpretation of the data, in the preparation of the manuscript, or in the decision to submit the manuscript for publication.  Authors' judgement: Low risks |
| CAPRIE; only in a sensitivity analysis | | | | | | | |
| The information generated by the sequence is unknown.  Authors' judgement: unclear risks | Patients were allocated study drugs sequentially from supplies at the clinical centre packaged in a predetermined order in a carton that contained supplies for four patients. These supplies were in the form of blister packs containing either 75 mg tablets of clopidogrel plus aspirin placebo tablets or 325 mg aspirin tablets plus clopidogrel placebo tablets, such blister packs being indistinguishable from one another.  Authors' judgement: Low risks | | Blinding was set to the patients and the implementation process of blinding was considered to be well maintained. And because the endpoint events are objective events, there is no subjective evaluation, so the blind method will not be destroyed.  Authors' judgement: Low risks | This committee had an associated Independent Statistical Centre in Lyon, France, that received an updated copy of the study database every 3 months from the Coordinating and Methods Centre. Information on study-drug allocation was merged with study data and routine aggregate safety summaries produced.  Authors' judgement: Low risks | During the study, 42 patients (0.22%) were lost to follow-up, 22 in the clopidogrel group and 20 in the aspirin group.  Authors' judgement: Low risks | The lack of pre-published protocol makes it impossible to make clear whether it is completely consistent with the previous plan, which may be related to the earlier implementation of the study.  Authors' judgement: unclear risks | The Central Validation Committee was responsible for validating all reported non-fatal outcome events and reported classifications of cause of death, with a secretariat at the Coordinating and Methods Centre in Hamilton, Ontario. After an outcome event dossier was received, only the secretariat had any communication with the reporting investigator about the validation of the event. The secretariat maintained a database of validated outcome events, a copy of which was not provided to the industrial backers before the end of the study.  Authors' judgement: Low risks |
| DAVID; only in a sensitivity analysis | | | | | | | |
| The information generated by the sequence is unknown.  Authors' judgement: unclear risks | Blinding was maintained by the use of indistinguishable active drugs and placebo tablets in separate bottles labelled for morning and evening intake.  Authors' judgement: Low risks | | Assignment was double-blinded. And because the endpoint events are objective events, there is no subjective evaluation, so the blind method will not be destroyed.  Authors' judgement: Low risks | The endpoint events are objective events, there is no subjective evaluation, so the blind method will not be destroyed.  Authors' judgement: Low risks | During the study, 32 patients (5.3%) in the picotamide group and 26 (4.3%) in the aspirin group were lost to follow-up and therefore had no endpoint data.  Authors' judgement: Low risks | The lack of pre-published protocol makes it impossible to make clear whether it is completely consistent with the previous plan, which may be related to the earlier implementation of the study.  Authors' judgement: unclear risks | N.B. Financial support to the study was provided by Novartis S.p.a., Milan, Italy, as payment of fees to an independent CRO in charge of monitoring.  Authors' judgement: Low risks |

## eFigure 3. Homogeneity assumption in network meta-analysis

The homogeneity assumption was completed by χ^2^-based Q-test, and if the P value was greater than 0.1, it was considered that the results were homogeneous, otherwise, there was heterogeneity. If the results were heterogeneous, the degree of heterogeneity was completed by I^2^ test (I2= 0–25%, no heterogeneity; I^2^= 25–50%, moderate heterogeneity; I^2^= 50–75%, large heterogeneity; I^2^= 75–100%, extreme heterogeneity). We conducted this investigation for the two primary outcomes (major adverse cardiovascular and cerebrovascular events and major bleeding) under the main network structure.

1. Major adverse cardiovascular and cerebrovascular events


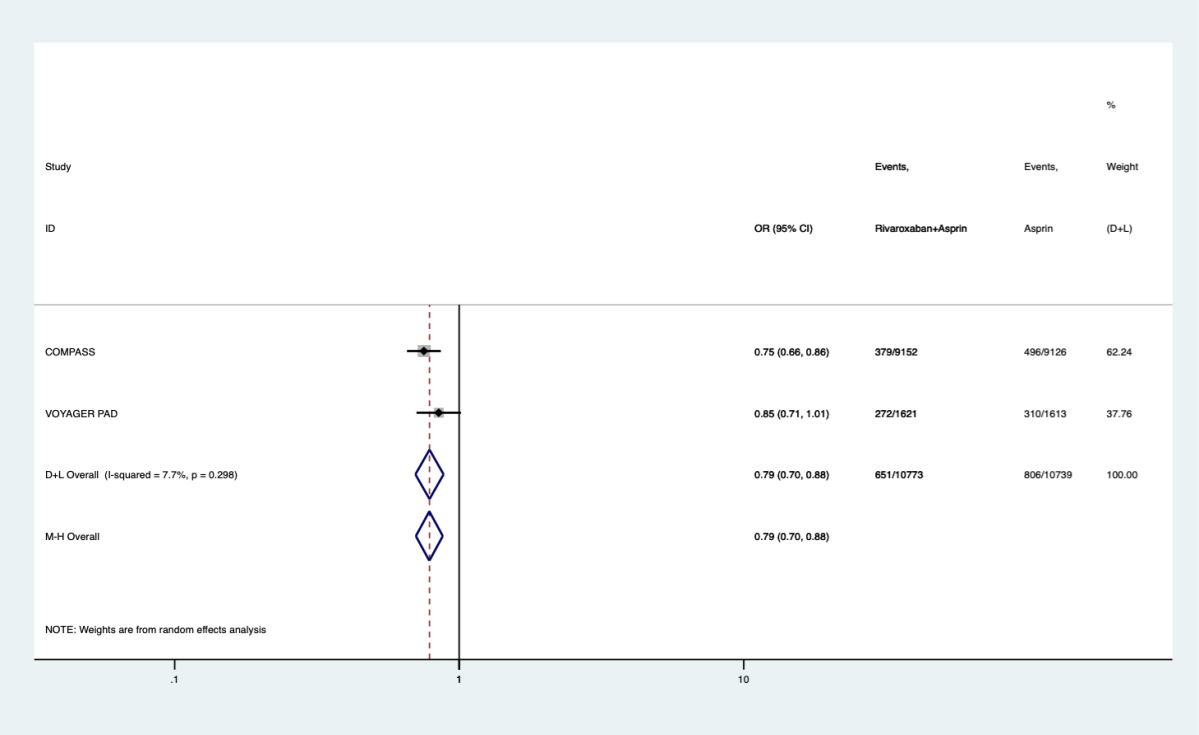


D+L: DerSimonian-Laird random effects model, M-H: Mantel-Haenszel fixed effects model.


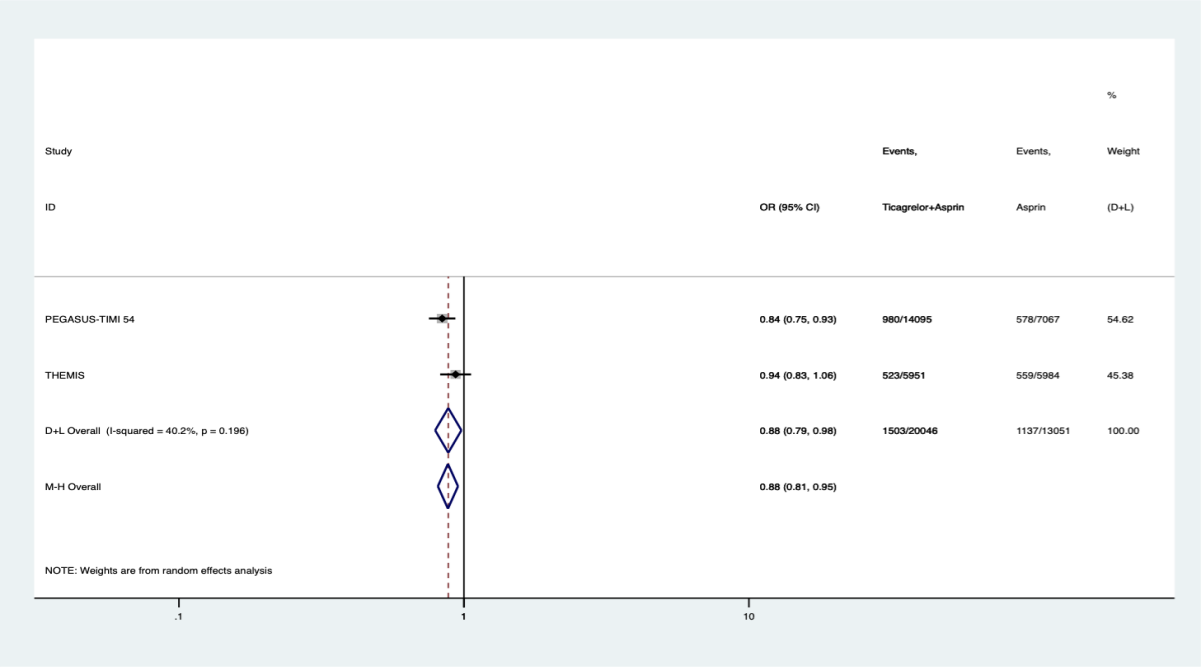


D+L: DerSimonian-Laird random effects model, M-H: Mantel-Haenszel fixed effects model.

1. Major bleeding


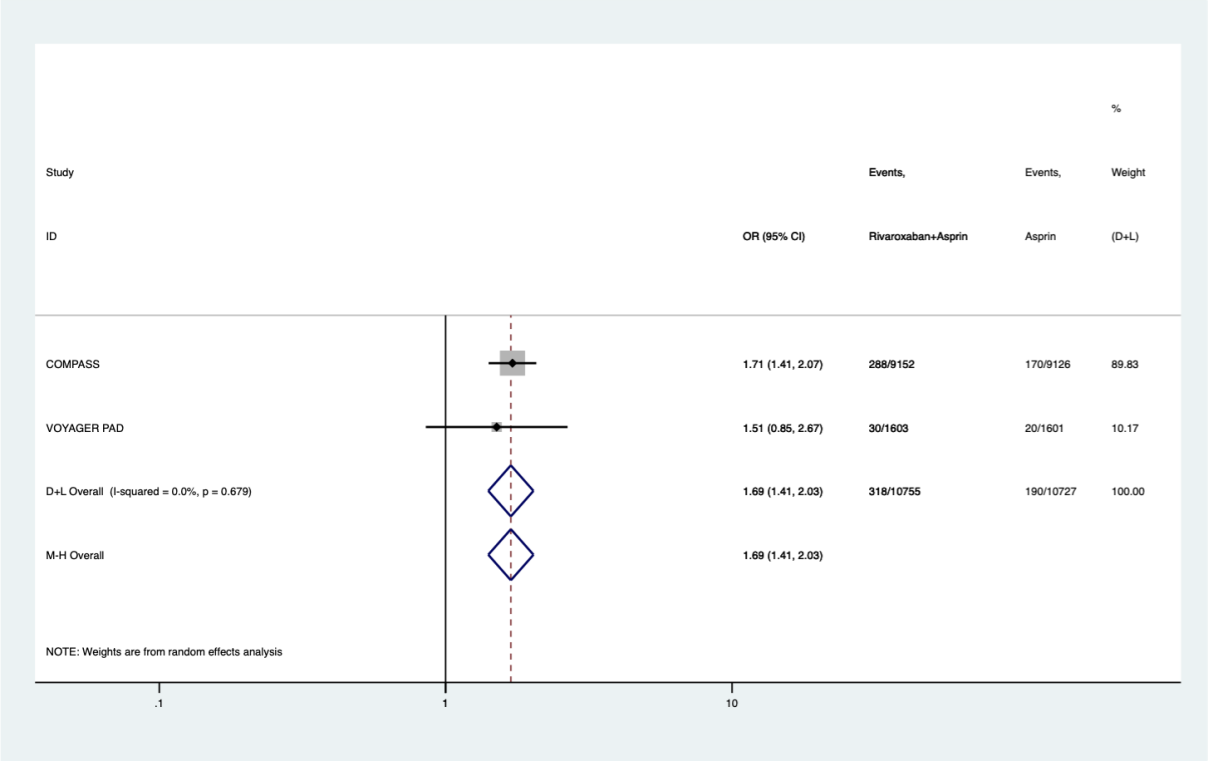


D+L: DerSimonian-Laird random effects model, M-H: Mantel-Haenszel fixed effects model.


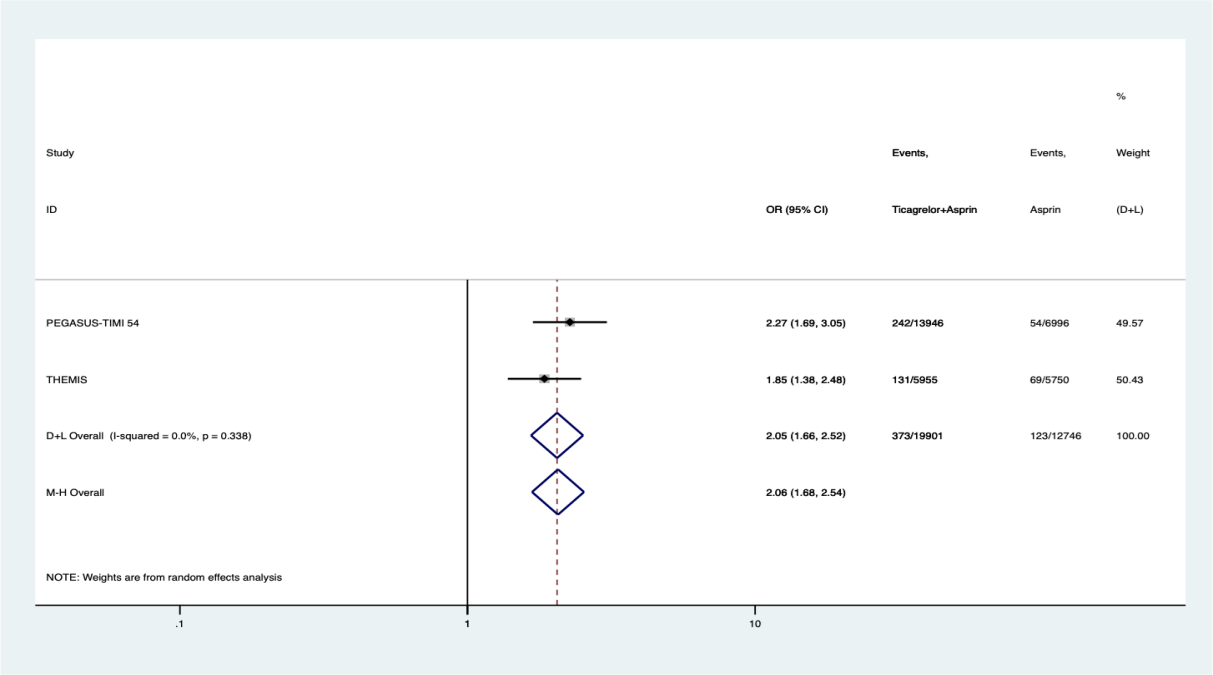


D+L: DerSimonian-Laird random effects model, M-H: Mantel-Haenszel fixed effects model.

## eFigure 4. Transitivity assumption in network meta-analysis

The transitivity assumption was completed by comparing the distribution of clinical variables, which were considered as interfering factors that might affect outcomes.

1. Age

1 = Asprin; 2 = Rivaroxaban+Asprin; 3 = Rivaroxaban; 4 = Ticagrelor+Asprin; Clopidogrel+Asprin; 6 = Ticagrelor; 7 = Clopidogrel;

1. Female

1 = Asprin; 2 = Rivaroxaban+Asprin; 3 = Rivaroxaban; 4 = Ticagrelor+Asprin; Clopidogrel+Asprin; 6 = Ticagrelor; 7 = Clopidogrel;

1. Hypertension

1 = Asprin; 2 = Rivaroxaban+Asprin; 3 = Rivaroxaban; 4 = Ticagrelor+Asprin; Clopidogrel+Asprin; 6 = Ticagrelor; 7 = Clopidogrel;

1. Diabetes

1 = Asprin; 2 = Rivaroxaban+Asprin; 3 = Rivaroxaban; 4 = Ticagrelor+Asprin; Clopidogrel+Asprin; 6 = Ticagrelor; 7 = Clopidogrel;

## eFigure 5. Consistency assumption in network meta-analysis

A design-by-treatment approach was used to assess inconsistency in the entire analytical network, and a loop-specific approach and node-splitting approach were used to assess local inconsistency. We conducted this investigation for the two primary outcomes (major adverse cardiovascular and cerebrovascular events and major bleeding) under the main network structure.

1. Major adverse cardiovascular and cerebrovascular events
2. Inconsistency in the entire analytical network

P_global inconsistency_ = 0.3174 > 0.05

1. loop-specific approach


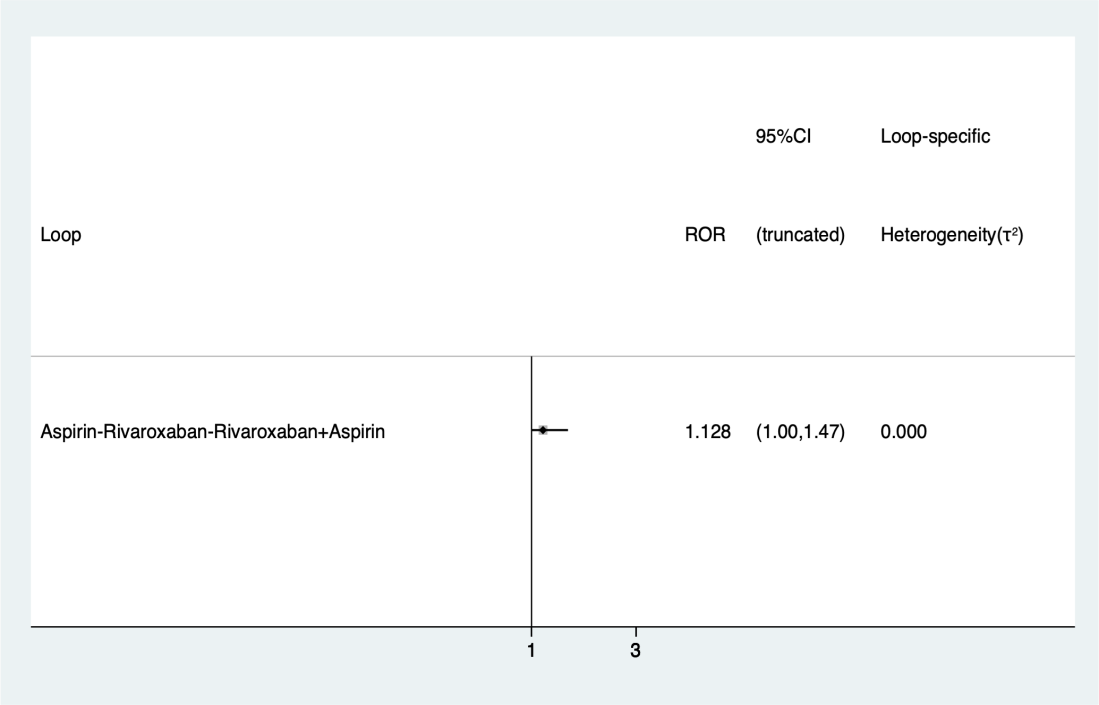


ROR represents the OR value of direct comparison divided by the OR value of indirect comparison. The 95% credible interval of the ROR included 1, which was considered to be the absence of local inconsistency.

1. Node-splitting approach

The node-splitting method was not applied to this network structure, as all the evidence about these contrasts came from trials which directly compare them.

1. Major bleeding

1) Inconsistency in the entire analytical network

P_global inconsistency_ = 0.6791 > 0.05

2) loop-specific approach


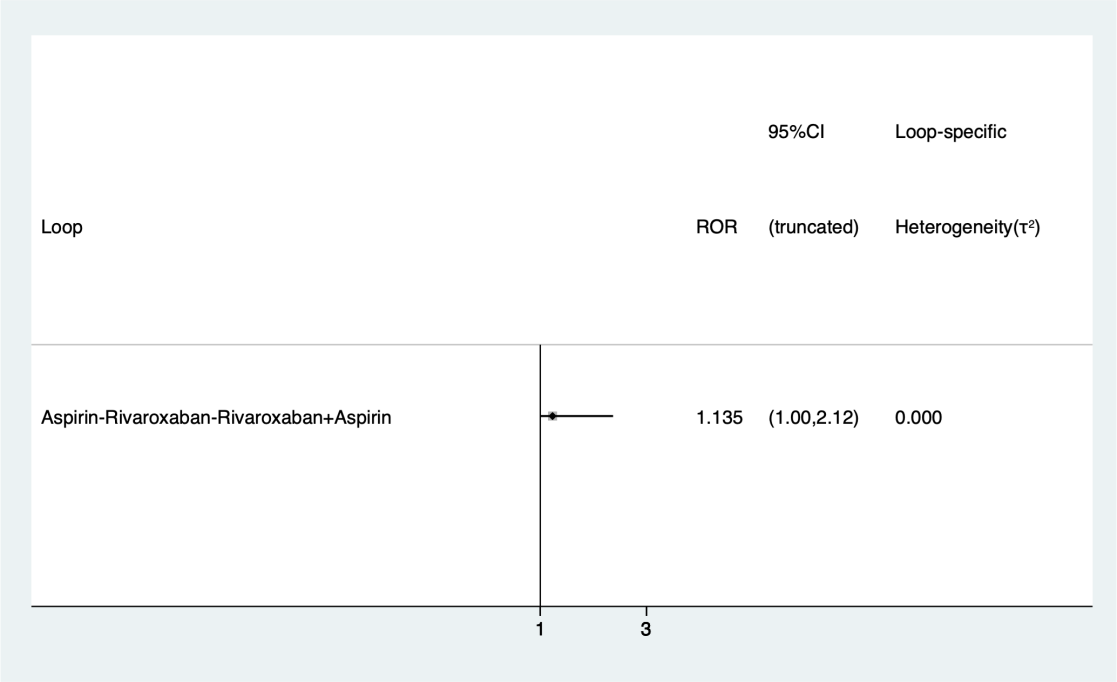


ROR represents the OR value of direct comparison divided by the OR value of indirect comparison. The 95% credible interval of the ROR included 1, which was considered to be the absence of local inconsistency.

3) Node-splitting approach

The node-splitting method was not applied to this network structure, as all the evidence about these contrasts came from trials which directly compare them.

# Additional results

## eTable 6. Pairwise comparison of efficacy and safety outcomes for the main analysis in the total cohort

Odds ratio (95% credible intervals) between column and row treatment regimens are reported. Odds ratio smaller than 1 means that the odds of having an event for the column treatment regimen is lower than the row treatment regimen. Statistically significant results, where the 95% credible interval does not include 1.

1. Major adverse cardiovascular and cerebrovascular events

| Clopidogrel | 0.84 (0.48,1.45) | 1.37 (0.85,2.20) | 1.18 (0.74,1.87) | 1.02 (0.89,1.17) | 1.31 (0.85,2.03) | 1.49 (0.95,2.34) |
| --- | --- | --- | --- | --- | --- | --- |
| 1.19 (0.69,2.06) | Clopidogrel+Aspirin | 1.64 (1.15,2.32) | 1.40 (1.00,1.97) | 1.22 (0.72,2.07) | 1.57 (1.12,2.19) | 1.78 (1.30,2.44) |
| 0.73 (0.45,1.17) | 0.61 (0.43,0.87) | Rivaroxaban | 0.86 (0.74,1.00) | 0.74 (0.47,1.17) | 0.96 (0.80,1.15) | 1.09 (0.94,1.26) |
| 0.85 (0.53,1.35) | 0.71 (0.51,1.00) | 1.17 (1.00,1.36) | Rivaroxaban+Aspirin | 0.87 (0.56,1.35) | 1.12 (0.95,1.31) | 1.27 (1.12,1.44) |
| 0.98 (0.85,1.12) | 0.82 (0.48,1.40) | 1.34 (0.85,2.11) | 1.15 (0.74,1.80) | Ticagrelor | 1.29 (0.85,1.95) | 1.46 (0.95,2.24) |
| 0.76 (0.49,1.18) | 0.64 (0.46,0.89) | 1.04 (0.87,1.25) | 0.89 (0.76,1.05) | 0.78 (0.51,1.18) | Ticagrelor+Aspirin | 1.14 (1.03,1.26) |
| 0.67 (0.43,1.05) | 0.56 (0.41,0.77) | 0.92 (0.79,1.07) | 0.79 (0.69,0.89) | 0.68 (0.45,1.05) | 0.88 (0.80,0.98) | Aspirin |

1. Major bleeding

| Clopidogrel | 1.79 (0.57,5.59) | 1.90 (0.87,4.13) | 2.13 (0.98,4.63) | 1.04 (0.80,1.36) | 2.58 (1.25,5.33) | 1.26 (0.59,2.68) |
| --- | --- | --- | --- | --- | --- | --- |
| 0.56 (0.18,1.74) | Clopidogrel+Aspirin | 1.06 (0.44,2.54) | 1.19 (0.50,2.84) | 0.58 (0.19,1.76) | 1.44 (0.60,3.47) | 0.70 (0.30,1.65) |
| 0.53 (0.24,1.15) | 0.94 (0.39,2.26) | Rivaroxaban | 1.12 (0.95,1.33) | 0.55 (0.26,1.14) | 1.36 (1.03,1.81) | 0.67 (0.55,0.81) |
| 0.47 (0.22,1.02) | 0.84 (0.35,2.01) | 0.89 (0.75,1.05) | Rivaroxaban+Aspirin | 0.49 (0.24,1.01) | 1.21 (0.92,1.60) | 0.59 (0.49,0.71) |
| 0.96 (0.74,1.25) | 1.72 (0.57,5.21) | 1.82 (0.88,3.79) | 2.05 (0.99,4.25) | Ticagrelor | 2.48 (1.26,4.87) | 1.21 (0.60,2.46) |
| 0.39 (0.19,0.80) | 0.69 (0.29,1.67) | 0.73 (0.55,0.97) | 0.82 (0.63,1.09) | 0.40 (0.21,0.79) | Ticagrelor+Aspirin | 0.49 (0.40,0.60) |
| 0.79 (0.37,1.69) | 1.42 (0.61,3.33) | 1.50 (1.24,1.82) | 1.69 (1.41,2.03) | 0.83 (0.41,1.67) | 2.05 (1.66,2.52) | Aspirin |

1. Death from any cause

| Clopidogrel | 1.53 (0.58,4.07) | 1.70 (0.75,3.86) | 1.44 (0.63,3.27) | 0.99 (0.88,1.11) | 1.66 (0.75,3.67) | 1.76 (0.79,3.94) |
| --- | --- | --- | --- | --- | --- | --- |
| 0.65 (0.25,1.74) | Clopidogrel+Aspirin | 1.11 (0.63,1.97) | 0.94 (0.53,1.67) | 0.65 (0.25,1.71) | 1.08 (0.61,1.92) | 1.15 (0.66,2.00) |
| 0.59 (0.26,1.33) | 0.90 (0.51,1.59) | Rivaroxaban | 0.85 (0.73,0.99) | 0.58 (0.26,1.31) | 0.97 (0.80,1.19) | 1.03 (0.89,1.20) |
| 0.69 (0.31,1.58) | 1.06 (0.60,1.89) | 1.18 (1.01,1.38) | Rivaroxaban+Aspirin | 0.69 (0.31,1.55) | 1.15 (0.94,1.41) | 1.22 (1.05,1.42) |
| 1.01 (0.90,1.13) | 1.54 (0.58,4.07) | 1.72 (0.76,3.86) | 1.45 (0.65,3.27) | Ticagrelor | 1.67 (0.76,3.67) | 1.77 (0.80,3.93) |
| 0.60 (0.27,1.33) | 0.92 (0.52,1.63) | 1.03 (0.84,1.25) | 0.87 (0.71,1.07) | 0.60 (0.27,1.31) | Ticagrelor+Aspirin | 1.06 (0.92,1.22) |
| 0.57 (0.25,1.27) | 0.87 (0.50,1.51) | 0.97 (0.84,1.12) | 0.82 (0.70,0.95) | 0.56 (0.25,1.25) | 0.94 (0.82,1.08) | Aspirin |

1. Cardiovascular death

| Clopidogrel | 1.58 (0.49,5.06) | 2.25 (0.92,5.52) | 1.83 (0.74,4.51) | 1.07 (0.88,1.29) | 1.98 (0.85,4.60) | 2.34 (0.98,5.58) |
| --- | --- | --- | --- | --- | --- | --- |
| 0.63 (0.20,2.04) | Clopidogrel+Aspirin | 1.43 (0.63,3.22) | 1.16 (0.51,2.63) | 0.68 (0.21,2.14) | 1.26 (0.56,2.82) | 1.49 (0.68,3.24) |
| 0.44 (0.18,1.09) | 0.70 (0.31,1.58) | Rivaroxaban | 0.81 (0.64,1.04) | 0.47 (0.20,1.14) | 0.88 (0.65,1.20) | 1.04 (0.83,1.31) |
| 0.55 (0.22,1.34) | 0.86 (0.38,1.94) | 1.23 (0.97,1.56) | Rivaroxaban+Aspirin | 0.58 (0.24,1.40) | 1.08 (0.79,1.49) | 1.28 (1.01,1.62) |
| 0.94 (0.78,1.14) | 1.48 (0.47,4.67) | 2.11 (0.88,5.08) | 1.72 (0.71,4.14) | Ticagrelor | 1.86 (0.82,4.22) | 2.20 (0.94,5.12) |
| 0.50 (0.22,1.17) | 0.79 (0.35,1.78) | 1.14 (0.83,1.55) | 0.92 (0.67,1.27) | 0.54 (0.24,1.22) | Ticagrelor+Aspirin | 1.18 (0.96,1.46) |
| 0.43 (0.18,1.02) | 0.67 (0.31,1.47) | 0.96 (0.76,1.21) | 0.78 (0.62,0.99) | 0.45 (0.20,1.06) | 0.85 (0.69,1.04) | Aspirin |

1. Miocardial infarction

| Clopidogrel | 0.64 (0.34,1.22) | 1.35 (0.78,2.34) | 1.31 (0.76,2.28) | 1.05 (0.90,1.23) | 1.25 (0.76,2.05) | 1.52 (0.91,2.54) |
| --- | --- | --- | --- | --- | --- | --- |
| 1.56 (0.82,2.95) | Clopidogrel+Aspirin | 2.10 (1.36,3.24) | 2.04 (1.32,3.15) | 1.64 (0.88,3.05) | 1.95 (1.30,2.93) | 2.37 (1.61,3.47) |
| 0.74 (0.43,1.29) | 0.48 (0.31,0.74) | Rivaroxaban | 0.97 (0.79,1.20) | 0.78 (0.46,1.32) | 0.93 (0.73,1.19) | 1.13 (0.92,1.38) |
| 0.76 (0.44,1.32) | 0.49 (0.32,0.76) | 1.03 (0.83,1.27) | Rivaroxaban+Aspirin | 0.80 (0.47,1.36) | 0.95 (0.75,1.22) | 1.16 (0.94,1.42) |
| 0.95 (0.81,1.11) | 0.61 (0.33,1.14) | 1.28 (0.76,2.18) | 1.25 (0.74,2.12) | Ticagrelor | 1.19 (0.75,1.90) | 1.45 (0.89,2.36) |
| 0.80 (0.49,1.31) | 0.51 (0.34,0.77) | 1.08 (0.84,1.38) | 1.05 (0.82,1.34) | 0.84 (0.53,1.34) | Ticagrelor+Aspirin | 1.21 (1.06,1.40) |
| 0.66 (0.39,1.10) | 0.42 (0.29,0.62) | 0.89 (0.72,1.09) | 0.86 (0.70,1.06) | 0.69 (0.42,1.13) | 0.82 (0.72,0.95) | Aspirin |

1. Ischemic stroke

| Clopidogrel | 1.29 (0.11,14.82) | 0.96 (0.08,11.04) | 0.77 (0.61,0.97) | 1.51 (0.14,16.91) | 1.88 (0.17,21.26) |
| --- | --- | --- | --- | --- | --- |
| 0.77 (0.07,8.89) | Rivaroxaban | 0.74 (0.54,1.02) | 0.60 (0.05,6.80) | 1.17 (0.81,1.69) | 1.46 (1.11,1.90) |
| 1.04 (0.09,12.01) | 1.35 (0.98,1.85) | Rivaroxaban+Aspirin | 0.81 (0.07,9.19) | 1.58 (1.08,2.32) | 1.96 (1.46,2.63) |
| 1.29 (1.03,1.63) | 1.67 (0.15,18.95) | 1.24 (0.11,14.11) | Ticagrelor | 1.96 (0.18,21.62) | 2.43 (0.22,27.18) |
| 0.66 (0.06,7.37) | 0.85 (0.59,1.23) | 0.63 (0.43,0.93) | 0.51 (0.05,5.64) | Ticagrelor+Aspirin | 1.24 (0.97,1.59) |
| 0.53 (0.05,6.02) | 0.69 (0.53,0.90) | 0.51 (0.38,0.68) | 0.41 (0.04,4.60) | 0.81 (0.63,1.03) | Aspirin |

1. Minor bleeding

| Clopidogrel+Aspirin | 0.36 (0.02,5.83) | 0.42 (0.03,5.68) | 0.82 (0.07,9.50) | 0.24 (0.02,2.61) |
| --- | --- | --- | --- | --- |
| 2.74 (0.17,43.81) | Rivaroxaban | 1.14 (0.32,4.10) | 2.24 (0.48,10.37) | 0.66 (0.16,2.72) |
| 2.41 (0.18,32.89) | 0.88 (0.24,3.16) | Rivaroxaban+Aspirin | 1.96 (0.57,6.72) | 0.58 (0.20,1.70) |
| 1.23 (0.11,14.27) | 0.45 (0.10,2.07) | 0.51 (0.15,1.74) | Ticagrelor+Aspirin | 0.29 (0.16,0.53) |
| 4.16 (0.38,45.04) | 1.52 (0.37,6.25) | 1.73 (0.59,5.08) | 3.39 (1.88,6.13) | Aspirin |

1. Intracranial hemorrhage

| Rivaroxaban | 0.65 (0.09,4.92) | 0.69 (0.01,62.41) | 0.56 (0.10,3.01) |
| --- | --- | --- | --- |
| 1.54 (0.20,11.73) | Rivaroxaban+Aspirin | 1.07 (0.01,148.24) | 0.86 (0.06,11.90) |
| 1.44 (0.02,130.22) | 0.94 (0.01,129.71) | Ticagrelor+Aspirin | 0.80 (0.01,52.19) |
| 1.80 (0.33,9.71) | 1.16 (0.08,16.12) | 1.24 (0.02,80.79) | Aspirin |

## eTable 7. Pairwise comparison of efficacy and safety outcomes in patients with coronary artery disease

Odds ratio (95% credible intervals) between column and row treatment regimens are reported. Odds ratio smaller than 1 means that the odds of having an event for the column treatment regimen is lower than the row treatment regimen. Statistically significant results, where the 95% credible interval does not include 1.

1. Major adverse cardiovascular and cerebrovascular events for main analysis

| Clopidogrel | 0.83 (0.47,1.47) | 1.31 (0.80,2.17) | 1.09 (0.66,1.81) | 1.01 (0.83,1.23) | 1.30 (0.82,2.06) | 1.48 (0.92,2.37) |
| --- | --- | --- | --- | --- | --- | --- |
| 1.20 (0.68,2.13) | Clopidogrel+Aspirin | 1.58 (1.10,2.27) | 1.31 (0.91,1.89) | 1.22 (0.71,2.08) | 1.57 (1.12,2.20) | 1.78 (1.29,2.45) |
| 0.76 (0.46,1.26) | 0.63 (0.44,0.91) | Rivaroxaban | 0.83 (0.70,0.99) | 0.77 (0.49,1.22) | 0.99 (0.81,1.21) | 1.12 (0.95,1.33) |
| 0.92 (0.55,1.51) | 0.76 (0.53,1.09) | 1.20 (1.01,1.43) | Rivaroxaban+Aspirin | 0.93 (0.58,1.47) | 1.19 (0.97,1.46) | 1.35 (1.14,1.61) |
| 0.99 (0.81,1.20) | 0.82 (0.48,1.40) | 1.30 (0.82,2.06) | 1.08 (0.68,1.72) | Ticagrelor | 1.29 (0.85,1.95) | 1.46 (0.95,2.25) |
| 0.77 (0.48,1.22) | 0.64 (0.46,0.89) | 1.01 (0.83,1.23) | 0.84 (0.68,1.03) | 0.78 (0.51,1.18) | Ticagrelor+Aspirin | 1.13 (1.02,1.26) |
| 0.68 (0.42,1.09) | 0.56 (0.41,0.77) | 0.89 (0.75,1.05) | 0.74 (0.62,0.88) | 0.68 (0.45,1.05) | 0.88 (0.79,0.98) | Aspirin |

1. Major adverse cardiovascular and cerebrovascular events for main analysis plus COMMANDER HF trial

| Clopidogrel | 1.07 (0.70,1.64) | 1.32 (0.75,2.33) | 0.90 (0.57,1.42) | 1.44 (0.89,2.31) | 1.27 (0.84,1.93) | 1.02 (0.76,1.38) | 1.34 (0.90,2.00) | 1.52 (1.03,2.25) |
| --- | --- | --- | --- | --- | --- | --- | --- | --- |
| 0.94 (0.61,1.44) | Clopidogrel+Aspirin | 1.23 (0.76,2.00) | 0.84 (0.60,1.19) | 1.35 (0.93,1.95) | 1.19 (0.89,1.59) | 0.96 (0.61,1.50) | 1.26 (0.91,1.74) | 1.42 (1.10,1.84) |
| 0.76 (0.43,1.34) | 0.81 (0.50,1.31) | Clopidogrel+Rivaroxaban | 0.69 (0.42,1.13) | 1.09 (0.64,1.86) | 0.97 (0.60,1.55) | 0.78 (0.43,1.39) | 1.02 (0.62,1.69) | 1.16 (0.73,1.84) |
| 1.11 (0.70,1.75) | 1.18 (0.84,1.66) | 1.46 (0.89,2.40) | Clopidogrel+Rivaroxaban+Aspirin | 1.59 (1.06,2.39) | 1.41 (1.02,1.95) | 1.13 (0.70,1.82) | 1.49 (1.03,2.15) | 1.69 (1.23,2.31) |
| 0.70 (0.43,1.12) | 0.74 (0.51,1.08) | 0.92 (0.54,1.56) | 0.63 (0.42,0.94) | Rivaroxaban | 0.89 (0.66,1.19) | 0.71 (0.44,1.16) | 0.93 (0.65,1.34) | 1.06 (0.79,1.42) |
| 0.79 (0.52,1.19) | 0.84 (0.63,1.12) | 1.03 (0.65,1.66) | 0.71 (0.51,0.98) | 1.13 (0.84,1.52) | Rivaroxaban+Aspirin | 0.80 (0.52,1.24) | 1.05 (0.78,1.42) | 1.20 (0.96,1.49) |
| 0.98 (0.72,1.32) | 1.05 (0.67,1.64) | 1.29 (0.72,2.32) | 0.88 (0.55,1.42) | 1.41 (0.87,2.29) | 1.25 (0.81,1.92) | Ticagrelor | 1.31 (0.89,1.94) | 1.49 (1.00,2.22) |
| 0.75 (0.50,1.11) | 0.80 (0.58,1.10) | 0.98 (0.59,1.62) | 0.67 (0.46,0.97) | 1.07 (0.75,1.53) | 0.95 (0.70,1.28) | 0.76 (0.52,1.12) | Ticagrelor+Aspirin | 1.13 (0.92,1.40) |
| 0.66 (0.44,0.97) | 0.70 (0.54,0.91) | 0.86 (0.54,1.38) | 0.59 (0.43,0.81) | 0.94 (0.70,1.27) | 0.84 (0.67,1.04) | 0.67 (0.45,1.00) | 0.88 (0.72,1.09) | Aspirin |

1. Major bleeding for main analysis

| Clopidogrel | 1.66 (0.50,5.54) | 1.77 (0.74,4.23) | 1.96 (0.82,4.69) | 0.97 (0.60,1.55) | 2.40 (1.06,5.46) | 1.17 (0.50,2.74) |
| --- | --- | --- | --- | --- | --- | --- |
| 0.60 (0.18,2.00) | Clopidogrel+Aspirin | 1.06 (0.44,2.55) | 1.18 (0.49,2.83) | 0.58 (0.19,1.76) | 1.44 (0.60,3.47) | 0.70 (0.30,1.65) |
| 0.56 (0.24,1.35) | 0.94 (0.39,2.26) | Rivaroxaban | 1.11 (0.93,1.33) | 0.55 (0.26,1.14) | 1.36 (1.01,1.82) | 0.66 (0.54,0.81) |
| 0.51 (0.21,1.22) | 0.85 (0.35,2.03) | 0.90 (0.75,1.08) | Rivaroxaban+Aspirin | 0.49 (0.24,1.03) | 1.22 (0.92,1.63) | 0.60 (0.49,0.73) |
| 1.03 (0.65,1.65) | 1.72 (0.57,5.21) | 1.83 (0.88,3.82) | 2.03 (0.97,4.23) | Ticagrelor | 2.48 (1.26,4.87) | 1.21 (0.60,2.46) |
| 0.42 (0.18,0.95) | 0.69 (0.29,1.67) | 0.74 (0.55,0.99) | 0.82 (0.61,1.09) | 0.40 (0.21,0.79) | Ticagrelor+Aspirin | 0.49 (0.40,0.60) |
| 0.85 (0.37,1.99) | 1.42 (0.61,3.33) | 1.51 (1.23,1.85) | 1.68 (1.37,2.05) | 0.83 (0.41,1.67) | 2.05 (1.66,2.52) | Aspirin |

1. Death from any cause for main analysis

| Clopidogrel | 1.72 (0.64,4.65) | 1.84 (0.79,4.26) | 1.50 (0.65,3.49) | 1.12 (0.91,1.37) | 1.87 (0.83,4.20) | 1.98 (0.87,4.51) |
| --- | --- | --- | --- | --- | --- | --- |
| 0.58 (0.22,1.57) | Clopidogrel+Aspirin | 1.07 (0.60,1.91) | 0.87 (0.49,1.56) | 0.65 (0.24,1.71) | 1.08 (0.61,1.92) | 1.15 (0.66,2.00) |
| 0.54 (0.23,1.26) | 0.94 (0.52,1.67) | Rivaroxaban | 0.82 (0.69,0.97) | 0.61 (0.27,1.37) | 1.01 (0.82,1.26) | 1.07 (0.91,1.26) |
| 0.66 (0.29,1.54) | 1.14 (0.64,2.04) | 1.22 (1.03,1.45) | Rivaroxaban+Aspirin | 0.74 (0.33,1.68) | 1.24 (0.99,1.55) | 1.31 (1.11,1.56) |
| 0.90 (0.73,1.10) | 1.54 (0.58,4.08) | 1.65 (0.73,3.73) | 1.35 (0.60,3.05) | Ticagrelor | 1.67 (0.76,3.67) | 1.77 (0.80,3.94) |
| 0.54 (0.24,1.21) | 0.92 (0.52,1.64) | 0.99 (0.80,1.22) | 0.81 (0.65,1.01) | 0.60 (0.27,1.31) | Ticagrelor+Aspirin | 1.06 (0.92,1.22) |
| 0.51 (0.22,1.15) | 0.87 (0.50,1.52) | 0.93 (0.79,1.09) | 0.76 (0.64,0.90) | 0.56 (0.25,1.25) | 0.94 (0.82,1.09) | Aspirin |

1. Cardiovascular death for main analysis

| Clopidogrel | 1.76 (0.54,5.76) | 2.49 (0.99,6.28) | 1.96 (0.77,4.94) | 1.19 (0.90,1.57) | 2.22 (0.93,5.27) | 2.62 (1.07,6.39) |
| --- | --- | --- | --- | --- | --- | --- |
| 0.57 (0.17,1.86) | Clopidogrel+Aspirin | 1.41 (0.63,3.20) | 1.11 (0.49,2.52) | 0.68 (0.21,2.14) | 1.26 (0.56,2.82) | 1.49 (0.68,3.24) |
| 0.40 (0.16,1.01) | 0.71 (0.31,1.60) | Rivaroxaban | 0.78 (0.61,1.01) | 0.48 (0.20,1.15) | 0.89 (0.65,1.22) | 1.05 (0.83,1.34) |
| 0.51 (0.20,1.29) | 0.90 (0.40,2.04) | 1.27 (0.99,1.64) | Rivaroxaban+Aspirin | 0.61 (0.25,1.48) | 1.13 (0.82,1.57) | 1.34 (1.04,1.72) |
| 0.84 (0.64,1.11) | 1.48 (0.47,4.68) | 2.09 (0.87,5.05) | 1.64 (0.68,3.97) | Ticagrelor | 1.86 (0.82,4.23) | 2.20 (0.94,5.13) |
| 0.45 (0.19,1.07) | 0.79 (0.35,1.78) | 1.12 (0.82,1.55) | 0.88 (0.64,1.23) | 0.54 (0.24,1.22) | Ticagrelor+Aspirin | 1.18 (0.96,1.46) |
| 0.38 (0.16,0.93) | 0.67 (0.31,1.47) | 0.95 (0.75,1.21) | 0.75 (0.58,0.96) | 0.45 (0.19,1.06) | 0.85 (0.69,1.04) | Aspirin |

## eTable 8. Pairwise comparison of efficacy and safety outcomes in patients with peripheral artery disease

Odds ratio (95% credible intervals) between column and row treatment regimens are reported. Odds ratio smaller than 1 means that the odds of having an event for the column treatment regimen is lower than the row treatment regimen. Statistically significant results, where the 95% credible interval does not include 1.

1. Major adverse cardiovascular and cerebrovascular events for main analysis

| Rivaroxaban | 0.88 (0.69,1.13) | 1.11 (0.88,1.41) |
| --- | --- | --- |
| 1.14 (0.89,1.45) | Rivaroxaban+Aspirin | 1.26 (1.06,1.50) |
| 0.90 (0.71,1.14) | 0.79 (0.67,0.94) | Aspirin |

1. Major adverse cardiovascular and cerebrovascular events for main analysis plus CAPRIE trial

| Clopidogrel | 1.18 (0.85,1.63) | 1.04 (0.79,1.37) | 1.02 (0.87,1.19) | 1.31 (1.06,1.63) |
| --- | --- | --- | --- | --- |
| 0.85 (0.61,1.17) | Rivaroxaban | 0.88 (0.69,1.13) | 0.87 (0.60,1.24) | 1.11 (0.88,1.41) |
| 0.96 (0.73,1.27) | 1.14 (0.89,1.45) | Rivaroxaban+Aspirin | 0.98 (0.71,1.35) | 1.26 (1.06,1.50) |
| 0.98 (0.84,1.15) | 1.16 (0.81,1.65) | 1.02 (0.74,1.40) | Ticagrelor | 1.29 (0.98,1.68) |
| 0.76 (0.61,0.95) | 0.90 (0.71,1.14) | 0.79 (0.67,0.94) | 0.78 (0.59,1.02) | Aspirin |

1. Major adverse cardiovascular and cerebrovascular events for main analysis plus DAVID trial

| Rivaroxaban | 0.88 (0.69,1.13) | 0.89 (0.54,1.46) | 1.11 (0.88,1.41) |
| --- | --- | --- | --- |
| 1.14 (0.89,1.45) | Rivaroxaban+Aspirin | 1.01 (0.63,1.62) | 1.26 (1.06,1.50) |
| 1.12 (0.68,1.84) | 0.99 (0.62,1.58) | Picotamide | 1.25 (0.81,1.93) |
| 0.90 (0.71,1.14) | 0.79 (0.67,0.94) | 0.80 (0.52,1.24) | Aspirin |

1. Major adverse cardiovascular and cerebrovascular events for main analysis plus CAPRIE and DAVID trials

| Clopidogrel | 1.18 (0.85,1.63) | 1.04 (0.79,1.37) | 1.02 (0.87,1.19) | 1.05 (0.65,1.71) | 1.31 (1.06,1.63) |
| --- | --- | --- | --- | --- | --- |
| 0.85 (0.61,1.17) | Rivaroxaban | 0.88 (0.69,1.13) | 0.87 (0.60,1.24) | 0.89 (0.54,1.46) | 1.11 (0.88,1.41) |
| 0.96 (0.73,1.27) | 1.14 (0.89,1.45) | Rivaroxaban+Aspirin | 0.98 (0.71,1.35) | 1.01 (0.63,1.62) | 1.26 (1.06,1.50) |
| 0.98 (0.84,1.15) | 1.16 (0.81,1.65) | 1.02 (0.74,1.40) | Ticagrelor | 1.03 (0.62,1.72) | 1.29 (0.98,1.68) |
| 0.95 (0.58,1.55) | 1.12 (0.68,1.84) | 0.99 (0.62,1.58) | 0.97 (0.58,1.62) | Picotamide | 1.25 (0.81,1.93) |
| 0.76 (0.61,0.95) | 0.90 (0.71,1.14) | 0.79 (0.67,0.94) | 0.78 (0.59,1.02) | 0.80 (0.52,1.24) | Aspirin |

1. Major bleeding for main analysis

| Rivaroxaban | 0.96 (0.70,1.31) | 0.60 (0.43,0.85) |
| --- | --- | --- |
| 1.04 (0.77,1.42) | Rivaroxaban+Aspirin | 0.63 (0.46,0.85) |
| 1.66 (1.18,2.34) | 1.59 (1.17,2.17) | Aspirin |

## eAppendix 1. Cumulative rank probability plot for efficacy and safety outcomes

The smaller the area under the curve, the lower the incidence of adverse events, which means the better treatment regimen performance. And a smaller surface under the cumulative ranking (SUCRA) value or a larger mean rank indicates a lower incidence of adverse outcomes, indicating better efficacy of the treatment regimen.

(1) Major adverse cardiovascular and cerebrovascular events for main analysis in the total cohort


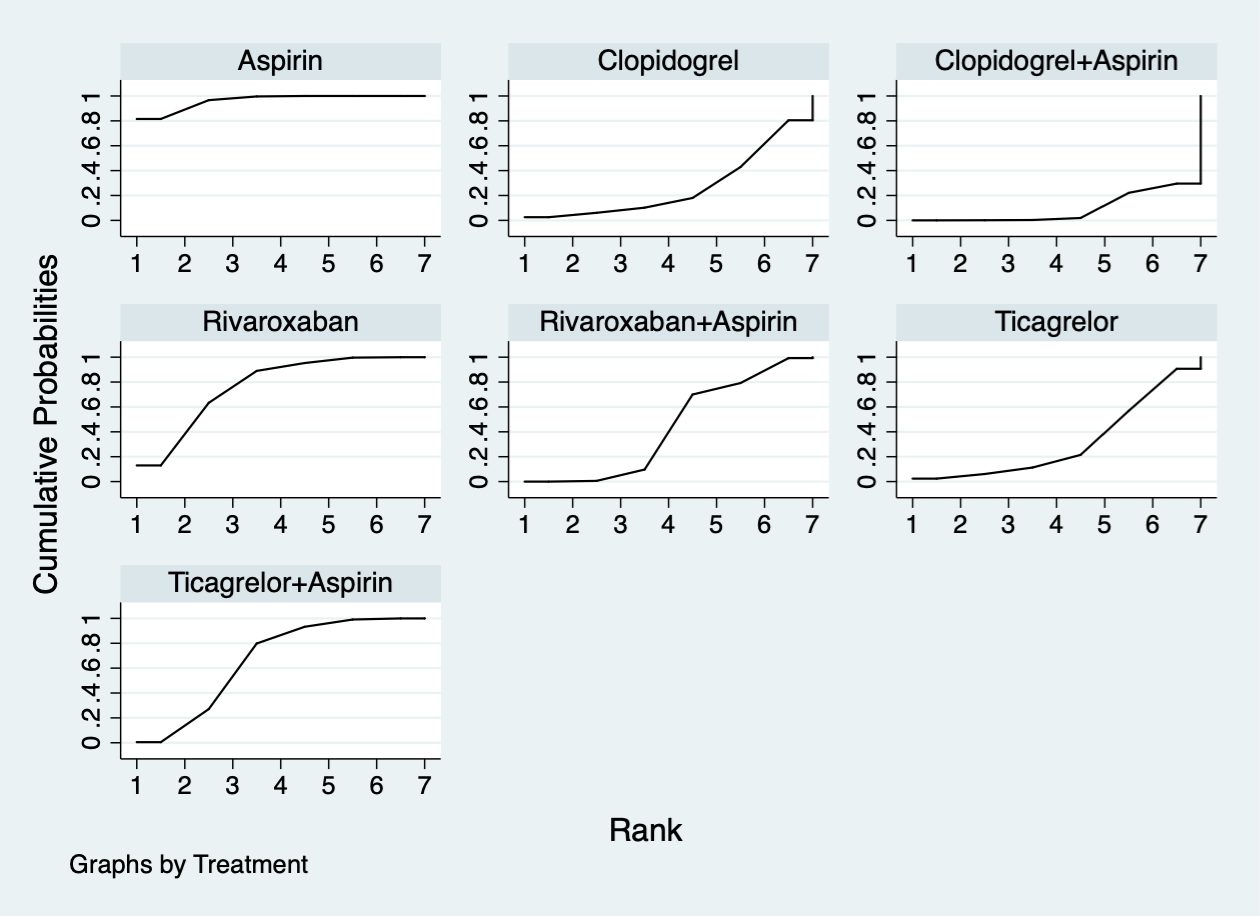


| Drug | Aspirin | Rivaroxaban | Rivaroxaban+Aspirin | Ticagrelor+Aspirin | Clopidogrel+Aspirin | Ticagrelor | Clopidogrel |
| --- | --- | --- | --- | --- | --- | --- | --- |
| Rank 1 | 0.815 | 0.13 | 0 | 0.005 | 0 | 0.024 | 0.025 |
| Rank 2 | 0.151 | 0.503 | 0.006 | 0.266 | 0.001 | 0.037 | 0.036 |
| Rank 3 | 0.03 | 0.257 | 0.091 | 0.527 | 0.002 | 0.052 | 0.041 |
| Rank 4 | 0.004 | 0.063 | 0.603 | 0.134 | 0.016 | 0.101 | 0.078 |
| Rank 5 | 0 | 0.043 | 0.092 | 0.058 | 0.202 | 0.355 | 0.249 |
| Rank 6 | 0 | 0.004 | 0.2 | 0.009 | 0.074 | 0.338 | 0.375 |
| Rank 7 | 0 | 0 | 0.007 | 0 | 0.704 | 0.093 | 0.195 |
| MeanRank | 1.223 | 2.398 | 4.406 | 2.998 | 6.454 | 5.112 | 5.392 |
| SUCRA | 96.3 | 76.7 | 43.1 | 66.6 | 9 | 31.5 | 26.7 |

(2) Major adverse cardiovascular and cerebrovascular events for main analysis plus CHARISMA trial in the total cohort


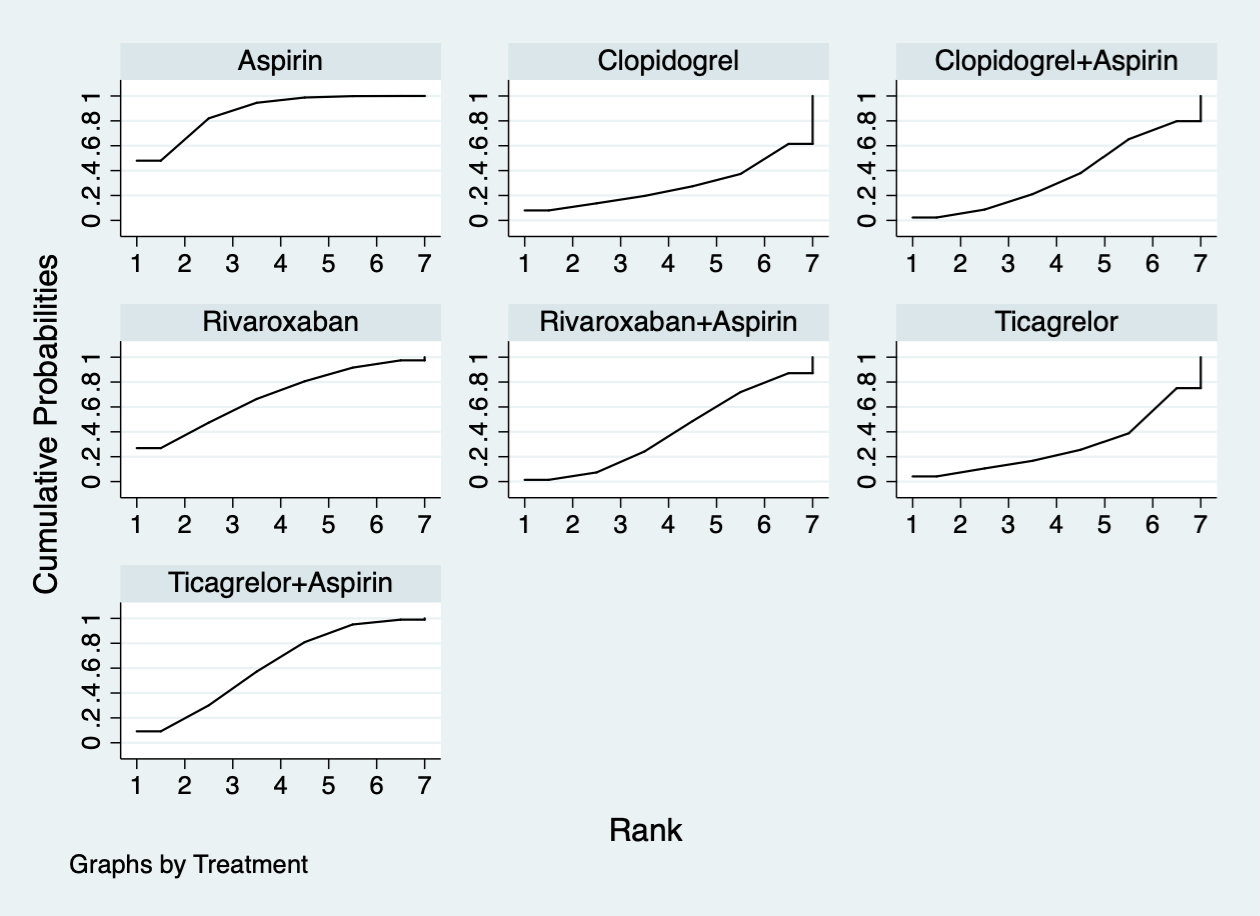


| Drug | Aspirin | Rivaroxaban | Rivaroxaban+Aspirin | Ticagrelor+Aspirin | Clopidogrel+Aspirin | Ticagrelor | Clopidogrel |
| --- | --- | --- | --- | --- | --- | --- | --- |
| Rank 1 | 0.48 | 0.269 | 0.014 | 0.092 | 0.023 | 0.042 | 0.08 |
| Rank 2 | 0.341 | 0.205 | 0.06 | 0.209 | 0.064 | 0.064 | 0.057 |
| Rank 3 | 0.125 | 0.19 | 0.169 | 0.271 | 0.124 | 0.061 | 0.059 |
| Rank 4 | 0.042 | 0.142 | 0.243 | 0.237 | 0.169 | 0.089 | 0.078 |
| Rank 5 | 0.011 | 0.11 | 0.233 | 0.141 | 0.273 | 0.133 | 0.099 |
| Rank 6 | 0.001 | 0.059 | 0.152 | 0.039 | 0.145 | 0.363 | 0.242 |
| Rank 7 | 0 | 0.025 | 0.128 | 0.01 | 0.203 | 0.249 | 0.385 |
| MeanRank | 1.766 | 2.896 | 4.586 | 3.28 | 4.855 | 5.295 | 5.325 |
| SUCRA | 87.2 | 68.4 | 40.1 | 61.9 | 35.9 | 28.5 | 28 |

(3) Major adverse cardiovascular and cerebrovascular events for main analysis plus CAPRIE trial in the total cohort


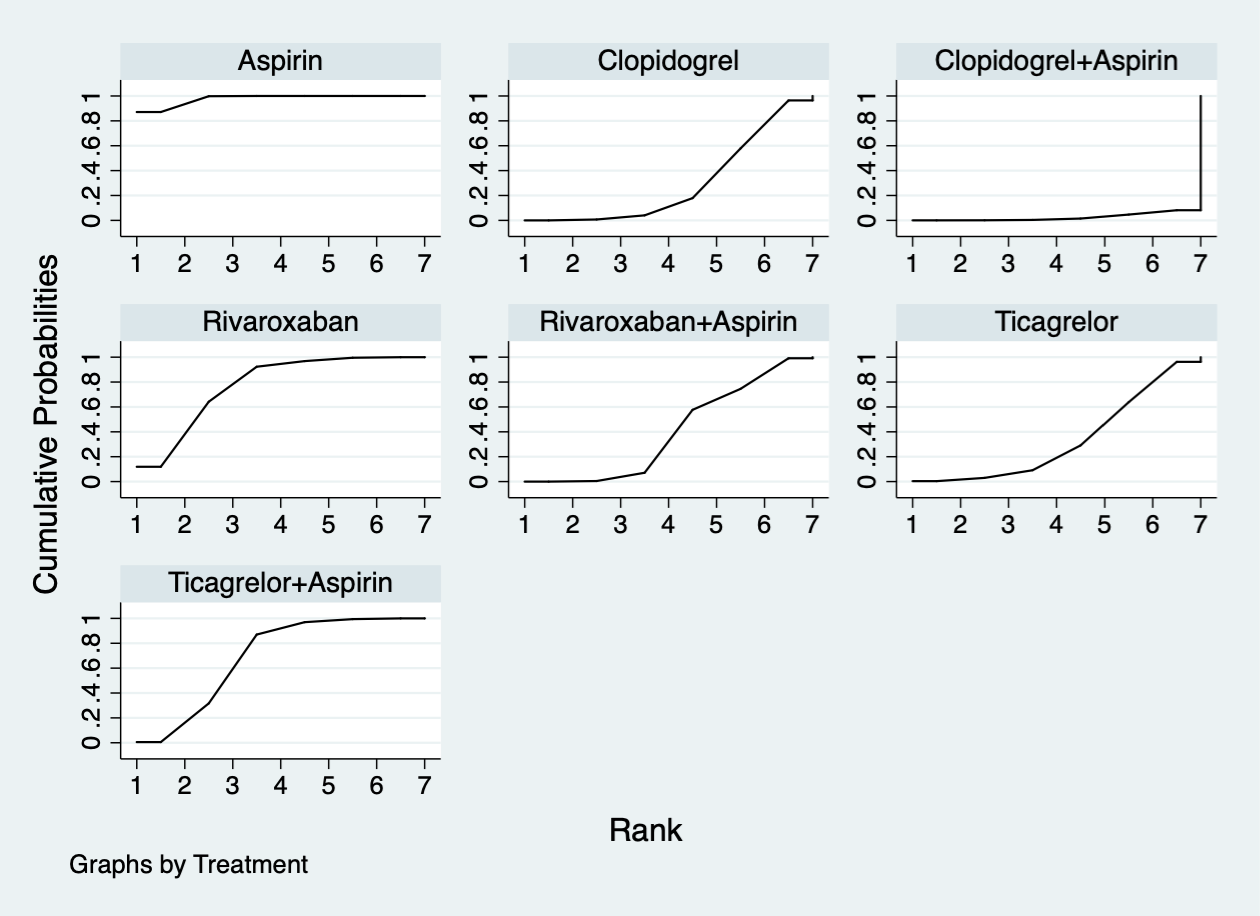


| Drug | Aspirin | Rivaroxaban | Rivaroxaban+Aspirin | Ticagrelor+Aspirin | Clopidogrel+Aspirin | Ticagrelor | Clopidogrel |
| --- | --- | --- | --- | --- | --- | --- | --- |
| Rank 1 | 0.871 | 0.119 | 0 | 0.006 | 0 | 0.004 | 0 |
| Rank 2 | 0.127 | 0.523 | 0.005 | 0.311 | 0.001 | 0.026 | 0.007 |
| Rank 3 | 0.002 | 0.281 | 0.066 | 0.553 | 0.003 | 0.061 | 0.033 |
| Rank 4 | 0 | 0.045 | 0.506 | 0.1 | 0.011 | 0.2 | 0.139 |
| Rank 5 | 0 | 0.027 | 0.169 | 0.024 | 0.032 | 0.348 | 0.4 |
| Rank 6 | 0 | 0.004 | 0.246 | 0.006 | 0.035 | 0.324 | 0.386 |
| Rank 7 | 0 | 0 | 0.009 | 0 | 0.919 | 0.037 | 0.036 |
| MeanRank | 1.131 | 2.347 | 4.616 | 2.843 | 6.858 | 4.982 | 5.237 |
| SUCRA | 97.8 | 77.5 | 39.8 | 69.3 | 2.5 | 33.6 | 29.5 |

(3) Major adverse cardiovascular and cerebrovascular events for main analysis plus COMMANDER HF trial in the total cohort


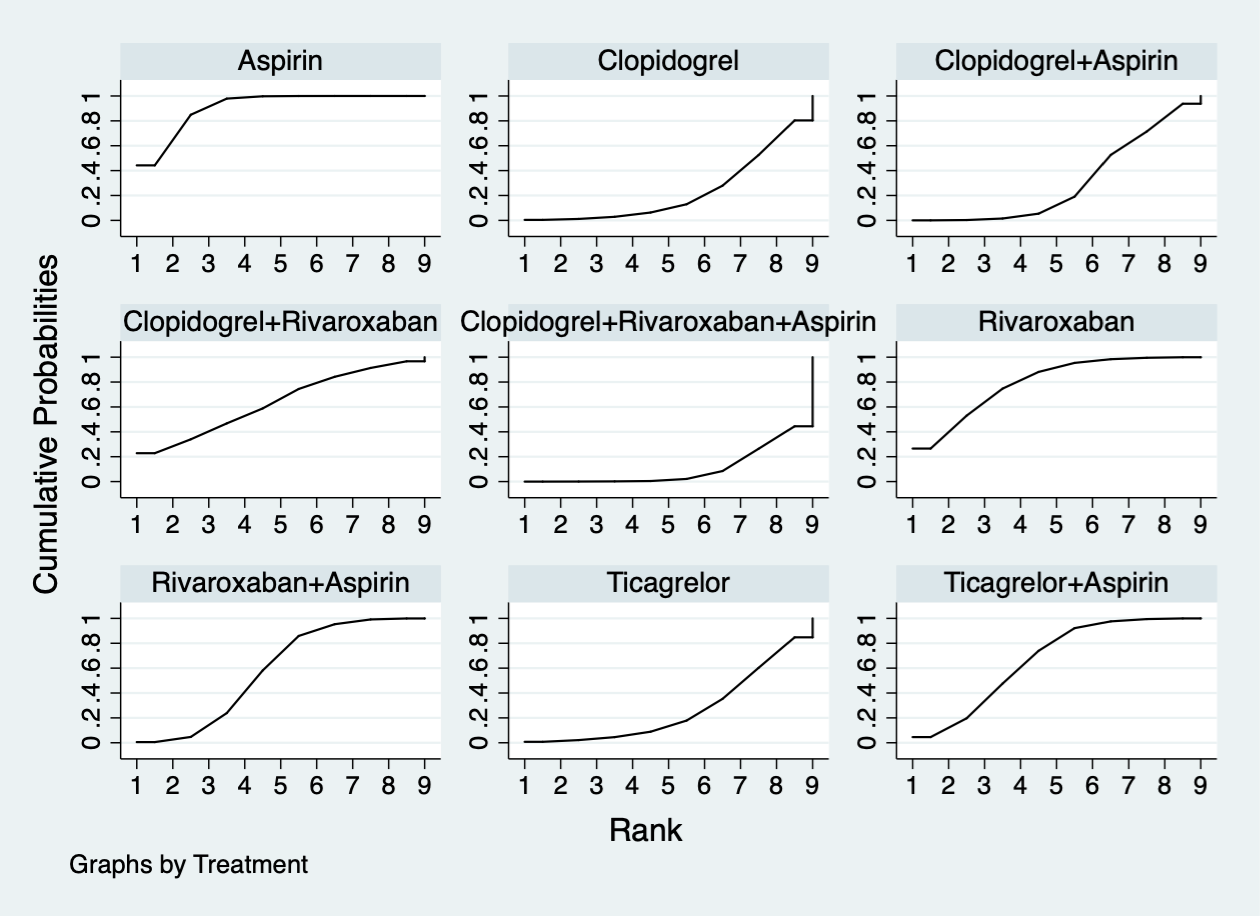


| Drug | Aspirin | Rivaroxaban | Rivaroxaban+Aspirin | Ticagrelor+Aspirin | Clopidogrel+Aspirin | Ticagrelor | Clopidogrel | Clopidogrel+Rivaroxaban | Clopidogrel+Rivaroxaban+Aspirin |
| --- | --- | --- | --- | --- | --- | --- | --- | --- | --- |
| Rank 1 | 0.442 | 0.266 | 0.006 | 0.046 | 0 | 0.008 | 0.004 | 0.228 | 0 |
| Rank 2 | 0.407 | 0.265 | 0.041 | 0.151 | 0.003 | 0.014 | 0.008 | 0.112 | 0 |
| Rank 3 | 0.13 | 0.217 | 0.192 | 0.279 | 0.012 | 0.024 | 0.017 | 0.128 | 0.001 |
| Rank 4 | 0.019 | 0.134 | 0.343 | 0.263 | 0.039 | 0.044 | 0.035 | 0.12 | 0.003 |
| Rank 5 | 0.002 | 0.072 | 0.277 | 0.182 | 0.136 | 0.089 | 0.066 | 0.156 | 0.017 |
| Rank 6 | 0 | 0.03 | 0.094 | 0.054 | 0.336 | 0.175 | 0.15 | 0.098 | 0.063 |
| Rank 7 | 0 | 0.011 | 0.038 | 0.018 | 0.188 | 0.249 | 0.246 | 0.071 | 0.179 |
| Rank 8 | 0 | 0.005 | 0.008 | 0.005 | 0.223 | 0.246 | 0.279 | 0.053 | 0.181 |
| Rank 9 | 0 | 0 | 0.001 | 0 | 0.062 | 0.152 | 0.196 | 0.033 | 0.555 |
| MeanRank | 1.732 | 2.64 | 4.324 | 3.637 | 6.552 | 6.858 | 7.159 | 3.902 | 8.174 |
| SUCRA | 90.9 | 79.5 | 58.5 | 66.9 | 30.5 | 26.8 | 23.1 | 63.6 | 10.3 |

1. Major adverse cardiovascular and cerebrovascular events for main analysis plus DAVID trial in the total cohort


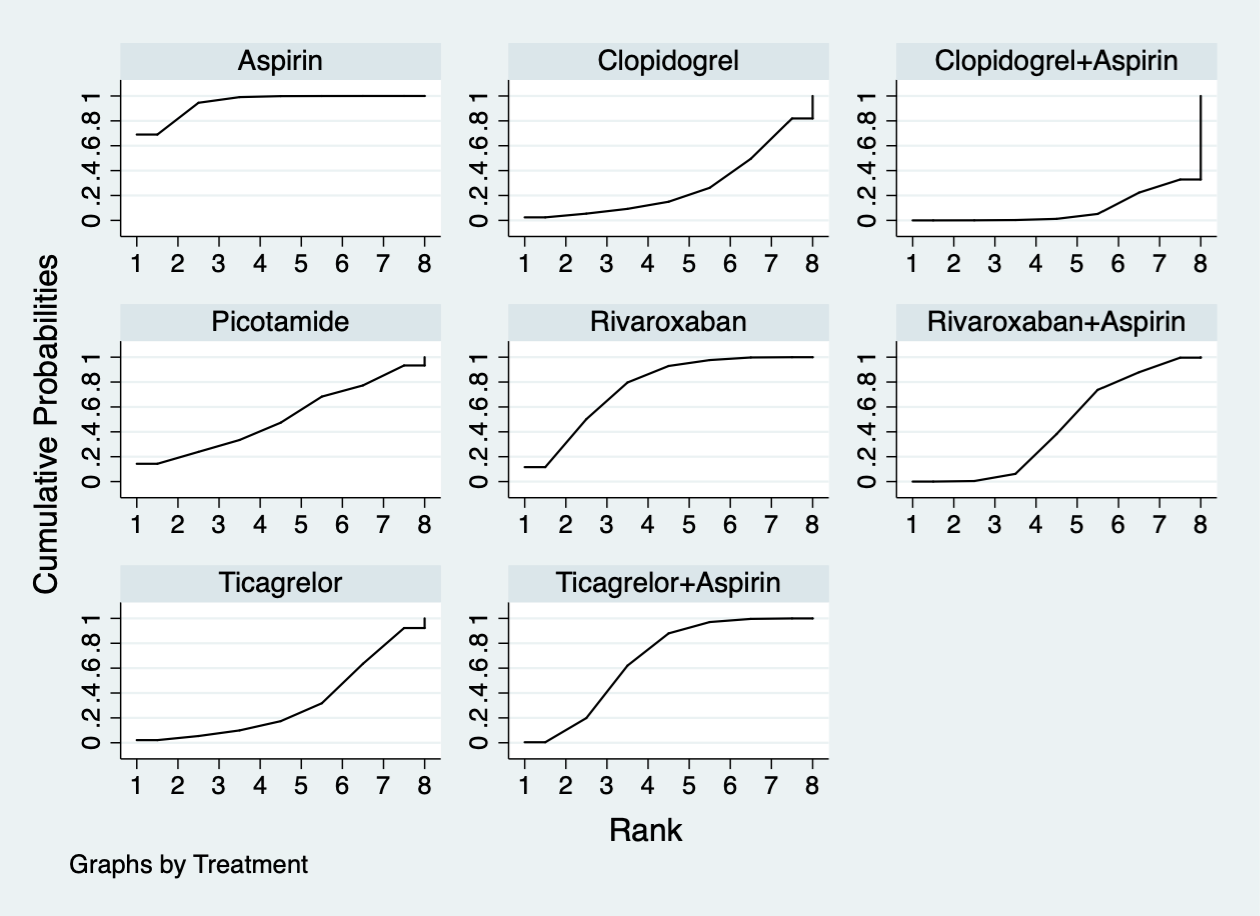


| Drug | Aspirin | Rivaroxaban | Rivaroxaban+Aspirin | Ticagrelor+Aspirin | Clopidogrel+Aspirin | Ticagrelor | Clopidogrel | Picotamide |
| --- | --- | --- | --- | --- | --- | --- | --- | --- |
| Rank 1 | 0.69 | 0.116 | 0 | 0.005 | 0 | 0.021 | 0.024 | 0.143 |
| Rank 2 | 0.256 | 0.386 | 0.005 | 0.195 | 0.001 | 0.033 | 0.03 | 0.096 |
| Rank 3 | 0.045 | 0.295 | 0.057 | 0.421 | 0.002 | 0.045 | 0.038 | 0.096 |
| Rank 4 | 0.008 | 0.133 | 0.32 | 0.259 | 0.009 | 0.074 | 0.057 | 0.139 |
| Rank 5 | 0.001 | 0.048 | 0.355 | 0.091 | 0.04 | 0.144 | 0.112 | 0.21 |
| Rank 6 | 0 | 0.021 | 0.142 | 0.025 | 0.171 | 0.317 | 0.234 | 0.09 |
| Rank 7 | 0 | 0.002 | 0.117 | 0.004 | 0.105 | 0.287 | 0.324 | 0.16 |
| Rank 8 | 0 | 0 | 0.004 | 0 | 0.671 | 0.077 | 0.181 | 0.067 |
| MeanRank | 1.374 | 2.685 | 4.939 | 3.327 | 7.373 | 5.765 | 6.106 | 4.425 |
| SUCRA | 94.6 | 76 | 43.7 | 66.7 | 8.9 | 31.8 | 27.1 | 51.1 |

1. Major adverse cardiovascular and cerebrovascular events for main analysis plus CHARISMA, CAPRIE, COMMANDER HF, and DAVID trials in the total cohort


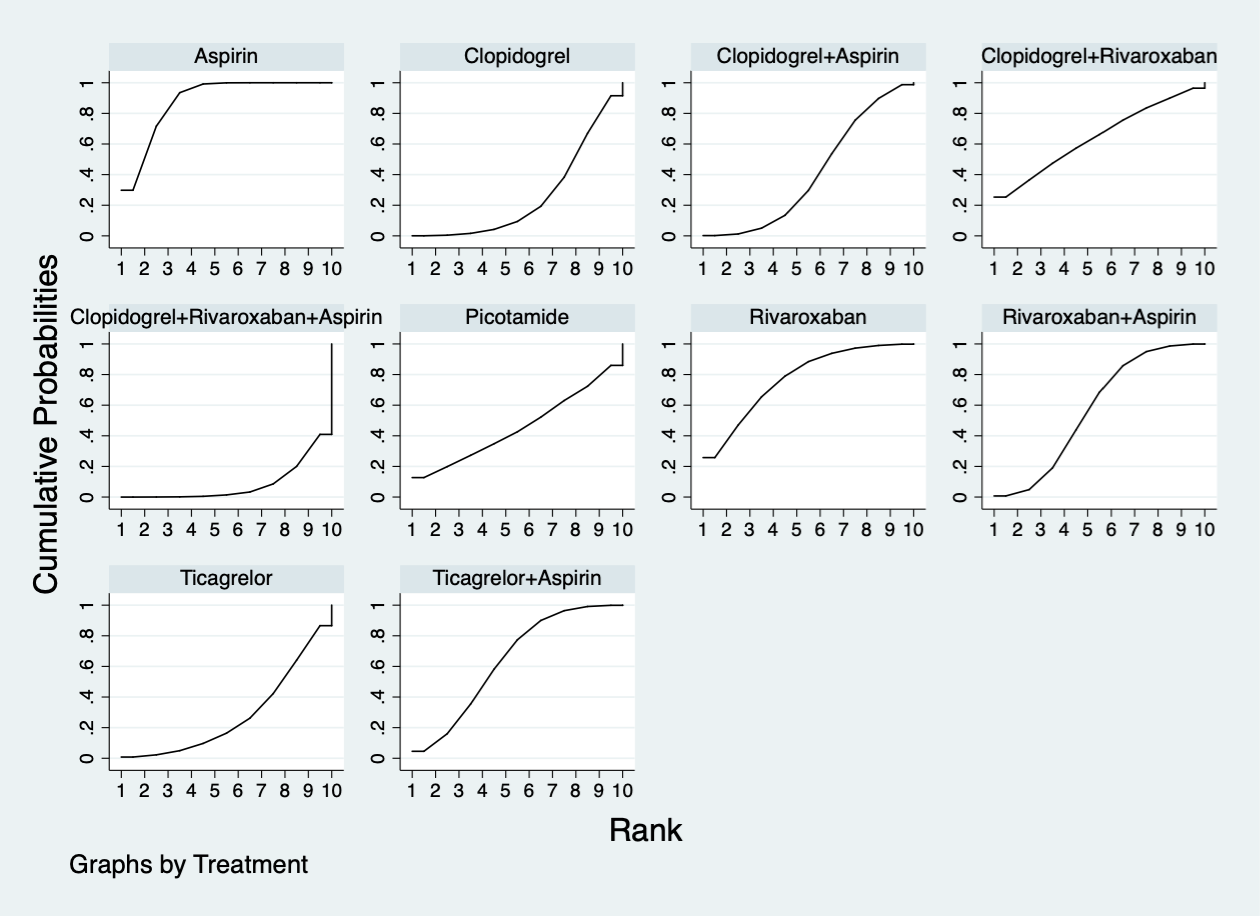


| Drug | Aspirin | Rivaroxaban | Rivaroxaban+Aspirin | Ticagrelor+Aspirin | Clopidogrel+Aspirin | Ticagrelor | Clopidogrel | Clopidogrel+Rivaroxaban | Clopidogrel+Rivaroxaban+Aspirin | Picotamide |
| --- | --- | --- | --- | --- | --- | --- | --- | --- | --- | --- |
| Rank 1 | 0.298 | 0.258 | 0.008 | 0.046 | 0.002 | 0.008 | 0.001 | 0.253 | 0 | 0.127 |
| Rank 2 | 0.419 | 0.213 | 0.041 | 0.115 | 0.011 | 0.014 | 0.004 | 0.112 | 0 | 0.072 |
| Rank 3 | 0.219 | 0.184 | 0.142 | 0.194 | 0.038 | 0.027 | 0.012 | 0.109 | 0.001 | 0.074 |
| Rank 4 | 0.057 | 0.135 | 0.247 | 0.227 | 0.084 | 0.047 | 0.026 | 0.098 | 0.003 | 0.075 |
| Rank 5 | 0.008 | 0.095 | 0.246 | 0.192 | 0.163 | 0.067 | 0.052 | 0.089 | 0.009 | 0.078 |
| Rank 6 | 0 | 0.054 | 0.174 | 0.126 | 0.24 | 0.098 | 0.099 | 0.094 | 0.019 | 0.096 |
| Rank 7 | 0 | 0.034 | 0.092 | 0.064 | 0.219 | 0.161 | 0.19 | 0.079 | 0.053 | 0.108 |
| Rank 8 | 0 | 0.017 | 0.036 | 0.027 | 0.142 | 0.218 | 0.287 | 0.064 | 0.114 | 0.093 |
| Rank 9 | 0 | 0.009 | 0.013 | 0.008 | 0.09 | 0.225 | 0.245 | 0.065 | 0.209 | 0.136 |
| Rank 10 | 0 | 0.001 | 0.001 | 0.001 | 0.013 | 0.134 | 0.085 | 0.035 | 0.59 | 0.14 |
| MeanRank | 2.061 | 2.986 | 4.663 | 4.102 | 6.098 | 7.366 | 7.585 | 4.111 | 9.219 | 5.787 |
| SUCRA | 88.2 | 77.3 | 57.4 | 64.1 | 40.8 | 28.1 | 25.8 | 64.2 | 8.3 | 45.7 |

1. Major adverse cardiovascular and cerebrovascular events for network meta-analysis structure combining ticagrelor and clopidogrel into P2Y12 inhibitor in the total cohort


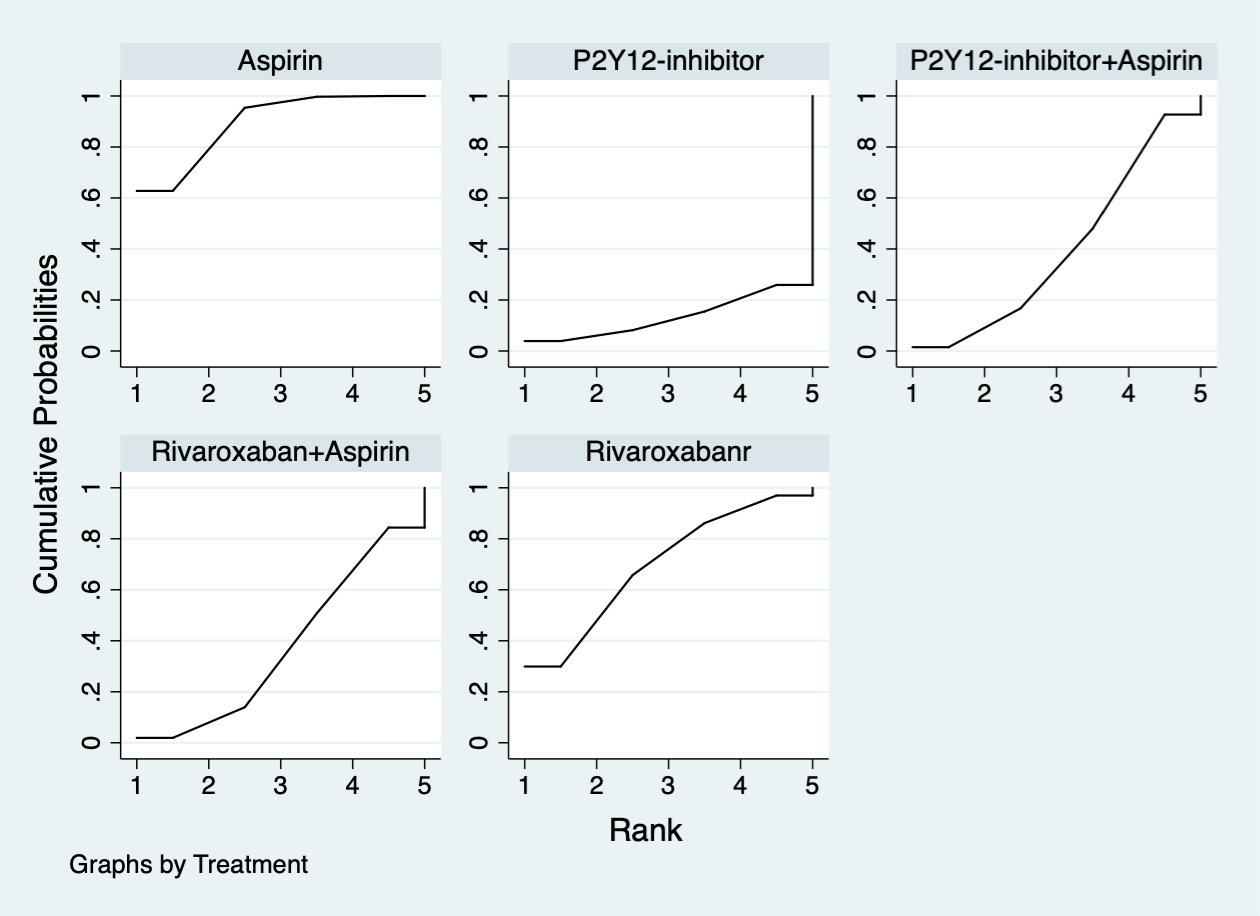


| Drug | Aspirin | Rivaroxaban | Rivaroxaban+Aspirin | P2Y12-inhibitor+Aspirin | P2Y12-inhibitor |
| --- | --- | --- | --- | --- | --- |
| Rank 1 | 0.628 | 0.299 | 0.019 | 0.015 | 0.039 |
| Rank 2 | 0.326 | 0.359 | 0.12 | 0.153 | 0.043 |
| Rank 3 | 0.043 | 0.204 | 0.368 | 0.312 | 0.073 |
| Rank 4 | 0.003 | 0.108 | 0.337 | 0.448 | 0.104 |
| Rank 5 | 0 | 0.03 | 0.156 | 0.073 | 0.741 |
| MeanRank | 1.421 | 2.211 | 3.491 | 3.414 | 4.465 |
| SUCRA | 89.5 | 69.7 | 37.7 | 39.7 | 13.4 |

(7) Major bleeding for main analysis in the total cohort


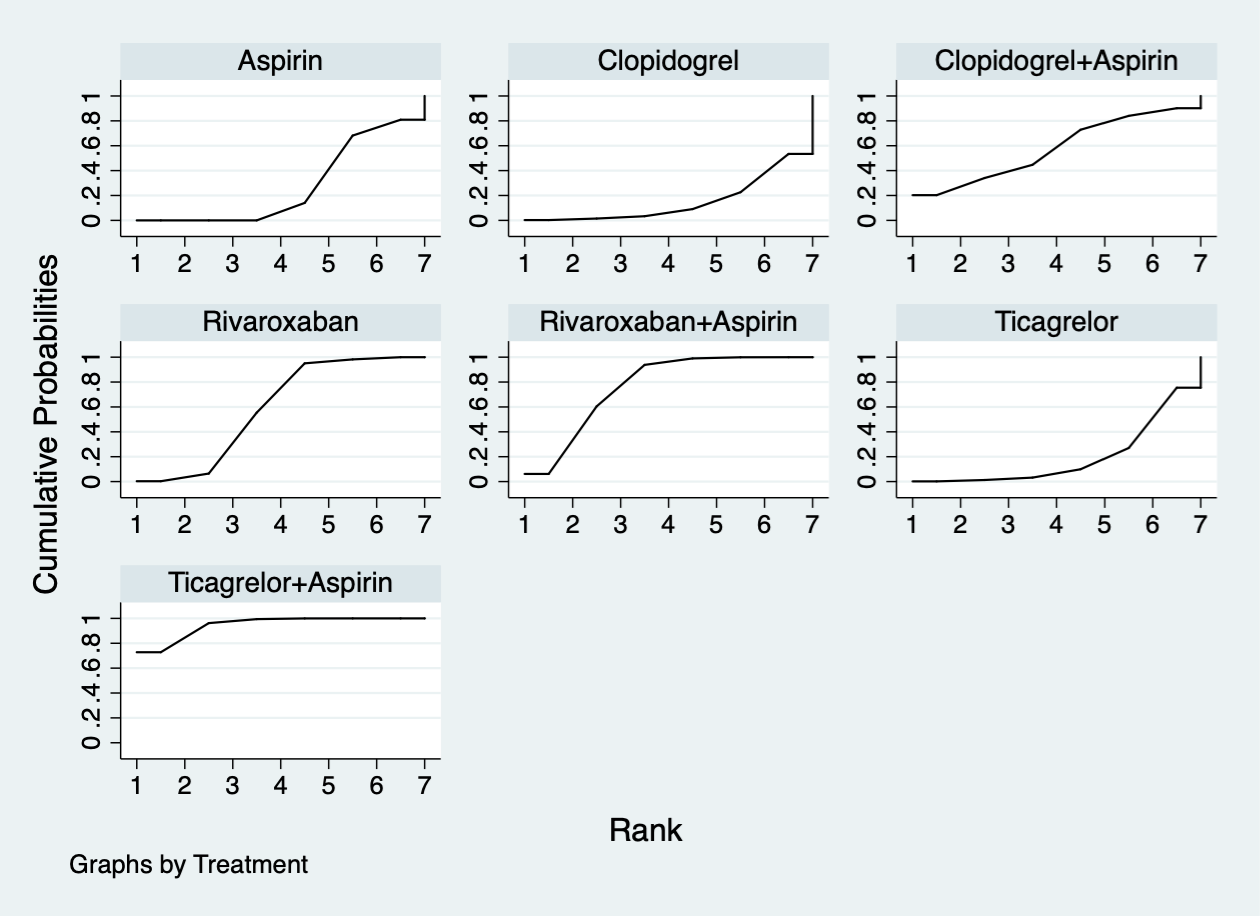


| Drug | Aspirin | Rivaroxaban | Rivaroxaban+Aspirin | Ticagrelor+Aspirin | Clopidogrel+Aspirin | Ticagrelor | Clopidogrel |
| --- | --- | --- | --- | --- | --- | --- | --- |
| Rank 1 | 0 | 0.003 | 0.062 | 0.728 | 0.203 | 0.002 | 0.003 |
| Rank 2 | 0 | 0.062 | 0.543 | 0.234 | 0.138 | 0.011 | 0.012 |
| Rank 3 | 0 | 0.49 | 0.334 | 0.032 | 0.106 | 0.02 | 0.019 |
| Rank 4 | 0.14 | 0.396 | 0.052 | 0.006 | 0.282 | 0.067 | 0.057 |
| Rank 5 | 0.541 | 0.031 | 0.009 | 0 | 0.111 | 0.171 | 0.135 |
| Rank 6 | 0.128 | 0.018 | 0.001 | 0 | 0.061 | 0.484 | 0.308 |
| Rank 7 | 0.191 | 0 | 0 | 0 | 0.098 | 0.245 | 0.466 |
| MeanRank | 5.37 | 3.444 | 2.409 | 1.316 | 3.532 | 5.826 | 6.097 |
| SUCRA | 27.2 | 59.3 | 76.6 | 94.7 | 57.7 | 19.5 | 15 |

1. Major bleeding for main analysis plus CHARISMA trial in the total cohort


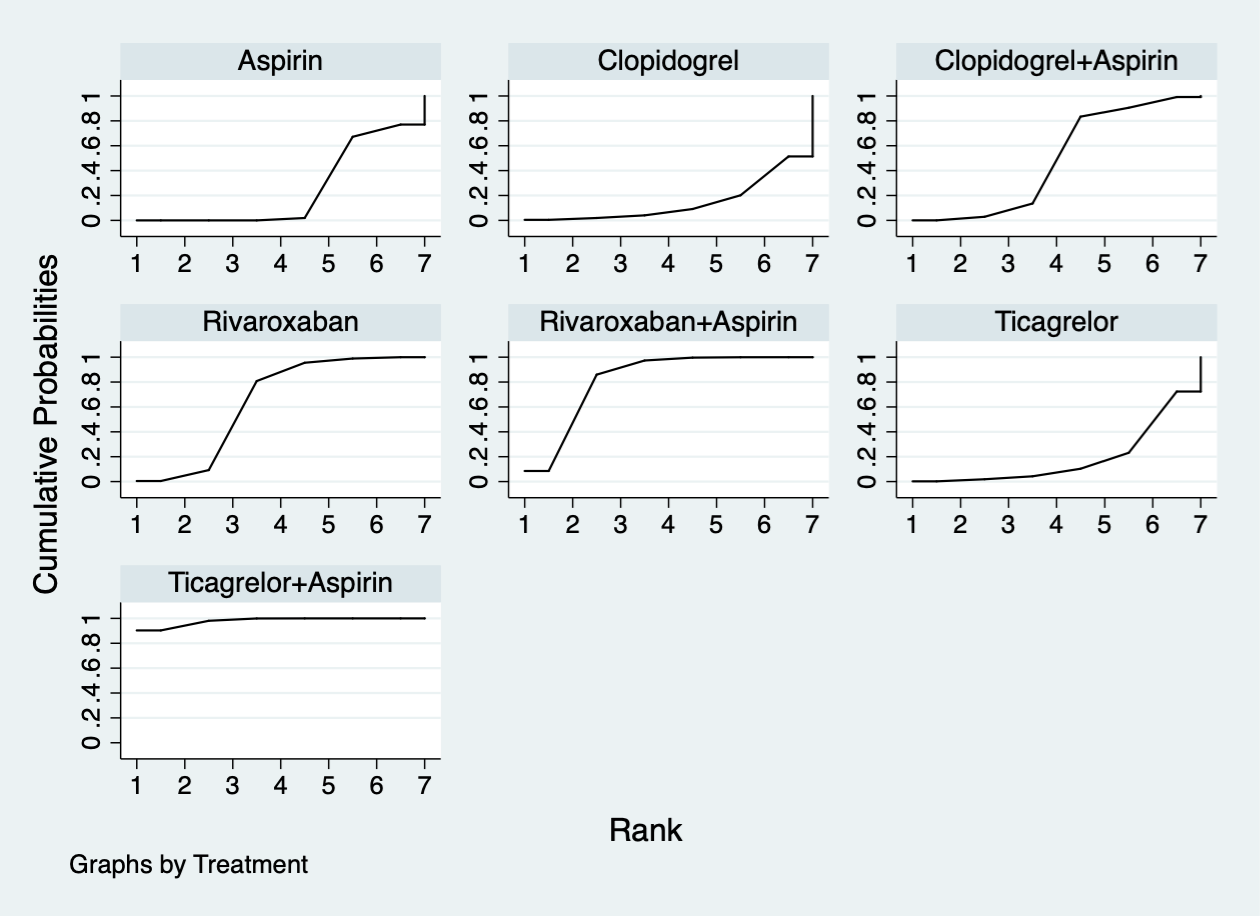


| Drug | Aspirin | Rivaroxaban | Rivaroxaban+Aspirin | Ticagrelor+Aspirin | Clopidogrel+Aspirin | Ticagrelor | Clopidogrel |
| --- | --- | --- | --- | --- | --- | --- | --- |
| Rank 1 | 0 | 0.005 | 0.085 | 0.903 | 0 | 0.002 | 0.004 |
| Rank 2 | 0 | 0.088 | 0.775 | 0.077 | 0.029 | 0.017 | 0.015 |
| Rank 3 | 0 | 0.715 | 0.114 | 0.019 | 0.107 | 0.024 | 0.021 |
| Rank 4 | 0.019 | 0.147 | 0.023 | 0.001 | 0.698 | 0.06 | 0.051 |
| Rank 5 | 0.653 | 0.034 | 0.003 | 0 | 0.071 | 0.129 | 0.11 |
| Rank 6 | 0.098 | 0.011 | 0 | 0 | 0.086 | 0.492 | 0.312 |
| Rank 7 | 0.23 | 0 | 0 | 0 | 0.009 | 0.276 | 0.486 |
| MeanRank | 5.539 | 3.15 | 2.084 | 1.118 | 4.105 | 5.877 | 6.125 |
| SUCRA | 24.4 | 64.2 | 81.9 | 98.1 | 48.3 | 18.7 | 14.5 |

1. Major bleeding for network meta-analysis structure combining ticagrelor and clopidogrel into P2Y12 inhibitor in the total cohort


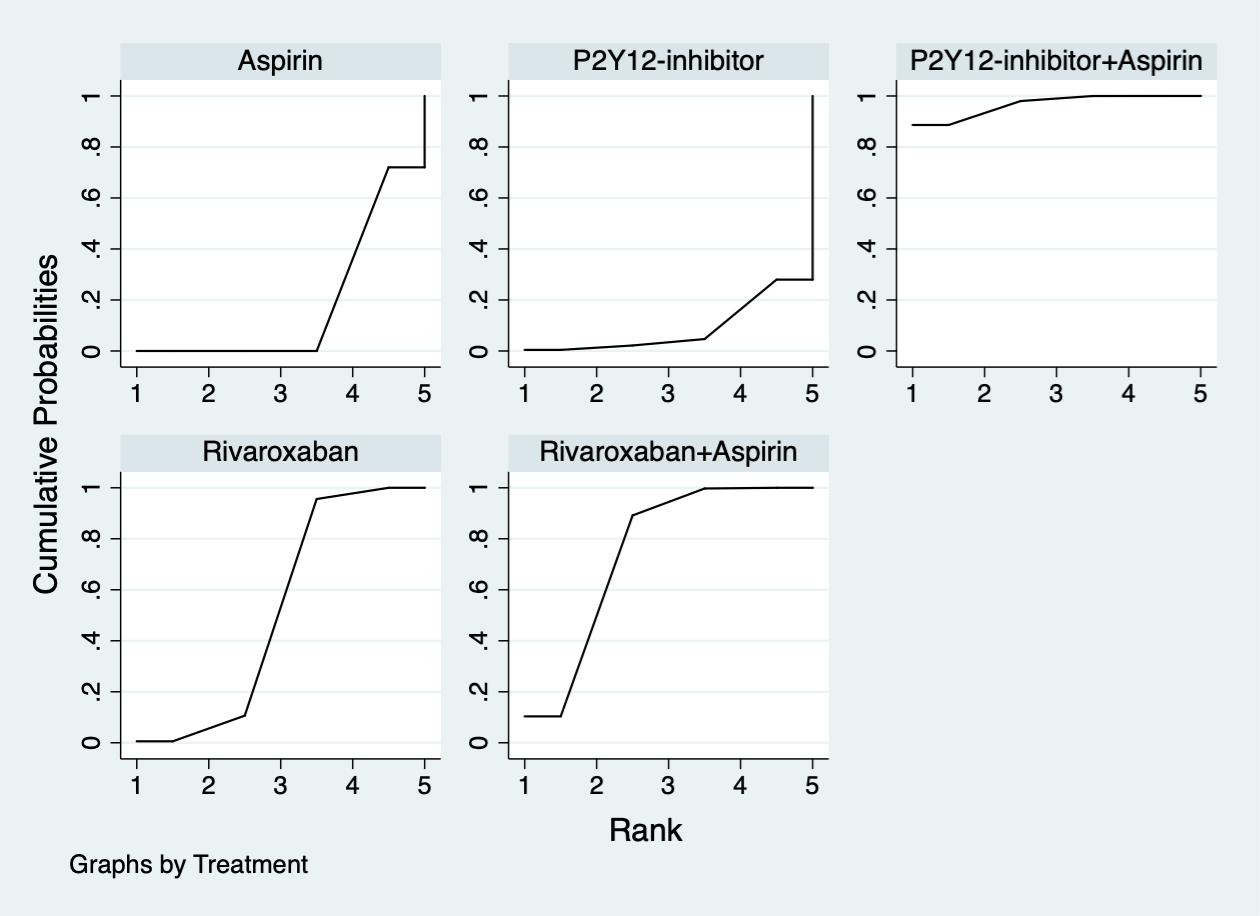


| Drug | Aspirin | Rivaroxaban | Rivaroxaban+Aspirin | P2Y12-inhibitor+Aspirin | P2Y12-inhibitor |
| --- | --- | --- | --- | --- | --- |
| Rank 1 | 0 | 0.006 | 0.103 | 0.886 | 0.004 |
| Rank 2 | 0 | 0.101 | 0.788 | 0.094 | 0.017 |
| Rank 3 | 0 | 0.85 | 0.106 | 0.02 | 0.025 |
| Rank 4 | 0.72 | 0.044 | 0.003 | 0 | 0.233 |
| Rank 5 | 0.28 | 0 | 0 | 0 | 0.72 |
| MeanRank | 4.28 | 2.934 | 2.009 | 1.134 | 4.645 |
| SUCRA | 18 | 51.7 | 74.8 | 96.7 | 8.8 |

1. TIMI major bleeding for network meta-analysis structure in the total cohort


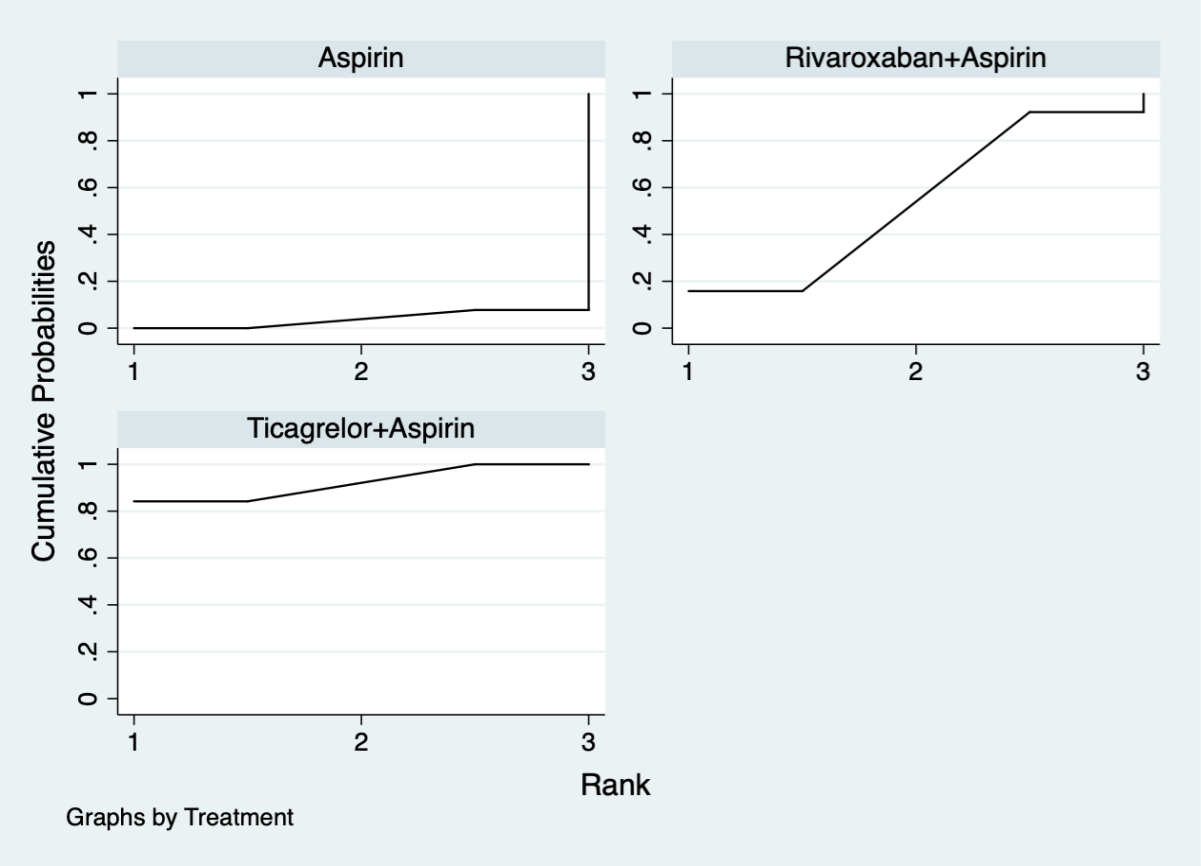


| Drug | Aspirin | Ticagrelor+Aspirin | Rivaroxaban+Aspirin |
| --- | --- | --- | --- |
| Rank 1 | 0 | 0.842 | 0.158 |
| Rank 2 | 0.078 | 0.158 | 0.764 |
| Rank 3 | 0.922 | 0 | 0.078 |
| MeanRank | 2.922 | 1.158 | 1.92 |
| SUCRA | 3.9 | 92.1 | 54 |

1. BARC (2, 3, or 5) bleeding for network meta-analysis structure in the total cohort


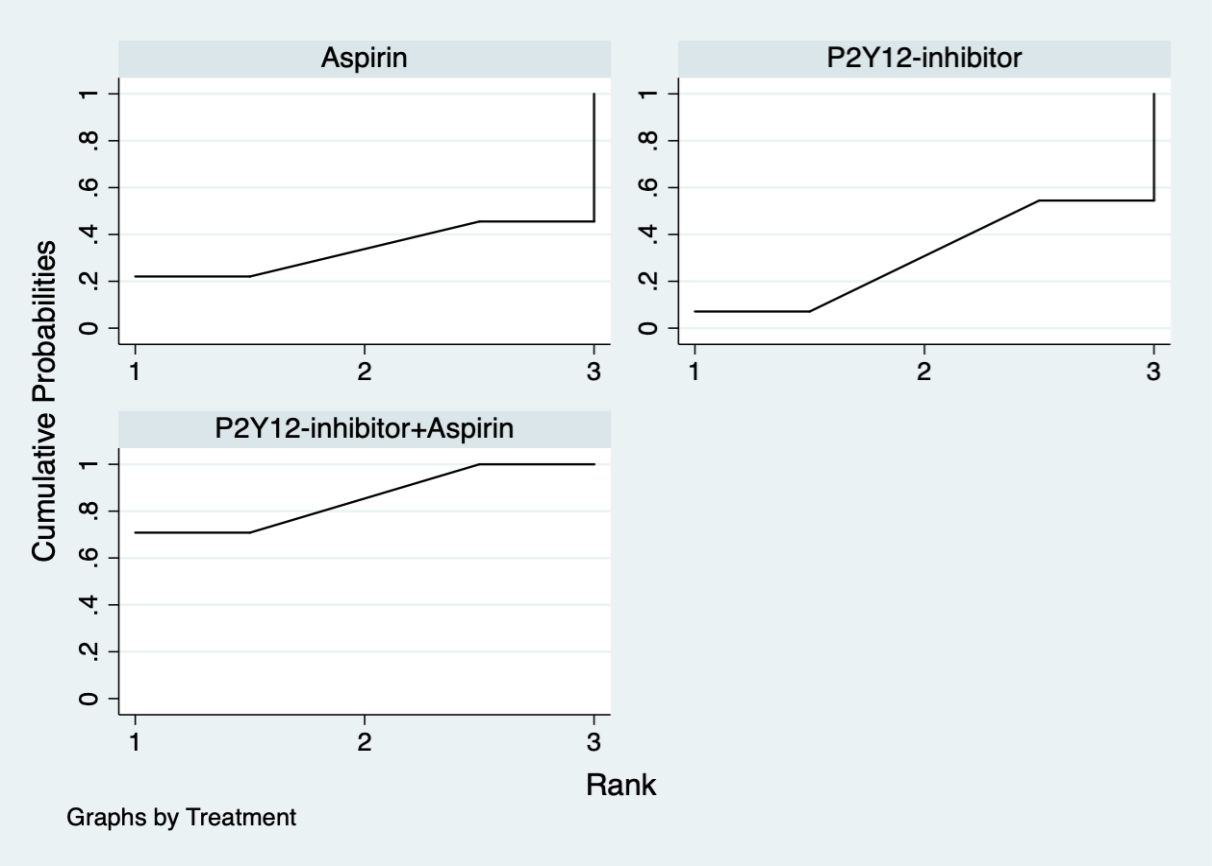


| Drug | Aspirin | P2Y12-inhibitor+Aspirin | P2Y12-inhibitor |
| --- | --- | --- | --- |
| Rank 1 | 0.221 | 0.708 | 0.071 |
| Rank 2 | 0.235 | 0.292 | 0.474 |
| Rank 3 | 0.545 | 0 | 0.455 |
| MeanRank | 2.326 | 1.292 | 2.384 |
| SUCRA | 33.8 | 85.4 | 30.8 |

1. Death from any cause for main analysis in the total cohort


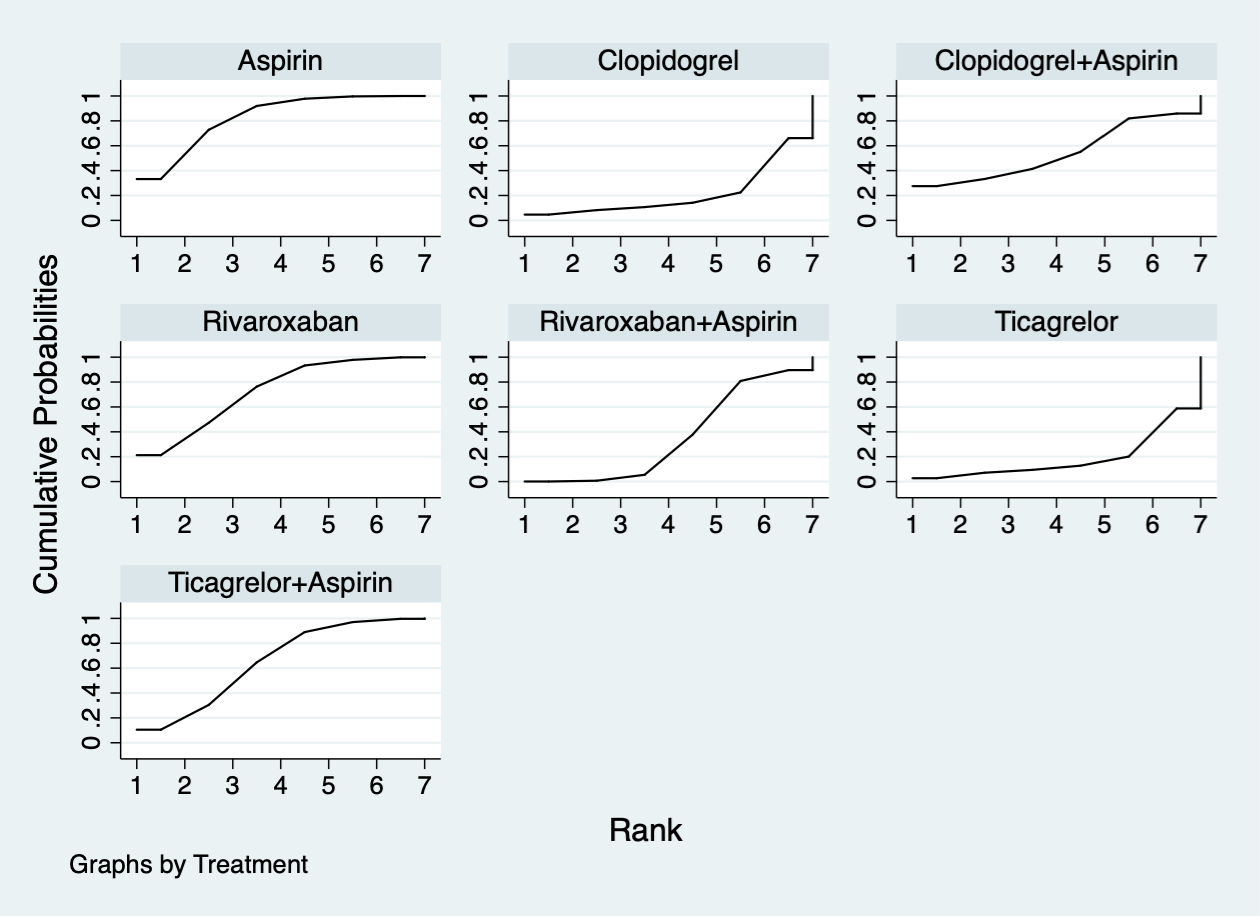


| Drug | Aspirin | Rivaroxaban | Rivaroxaban+Aspirin | Ticagrelor+Aspirin | Clopidogrel+Aspirin | Ticagrelor | Clopidogrel |
| --- | --- | --- | --- | --- | --- | --- | --- |
| Rank 1 | 0.332 | 0.213 | 0.001 | 0.105 | 0.275 | 0.027 | 0.046 |
| Rank 2 | 0.396 | 0.261 | 0.006 | 0.199 | 0.058 | 0.044 | 0.036 |
| Rank 3 | 0.192 | 0.29 | 0.048 | 0.342 | 0.081 | 0.023 | 0.024 |
| Rank 4 | 0.058 | 0.17 | 0.323 | 0.244 | 0.137 | 0.033 | 0.035 |
| Rank 5 | 0.019 | 0.045 | 0.432 | 0.08 | 0.268 | 0.073 | 0.083 |
| Rank 6 | 0.003 | 0.021 | 0.087 | 0.026 | 0.04 | 0.387 | 0.436 |
| Rank 7 | 0 | 0.001 | 0.104 | 0.003 | 0.141 | 0.412 | 0.339 |
| MeanRank | 2.045 | 2.643 | 4.859 | 3.082 | 3.749 | 5.887 | 5.734 |
| SUCRA | 82.6 | 72.7 | 35.7 | 65.2 | 54.2 | 18.5 | 21.1 |

1. Cardiovascular death for main analysis in the total cohort


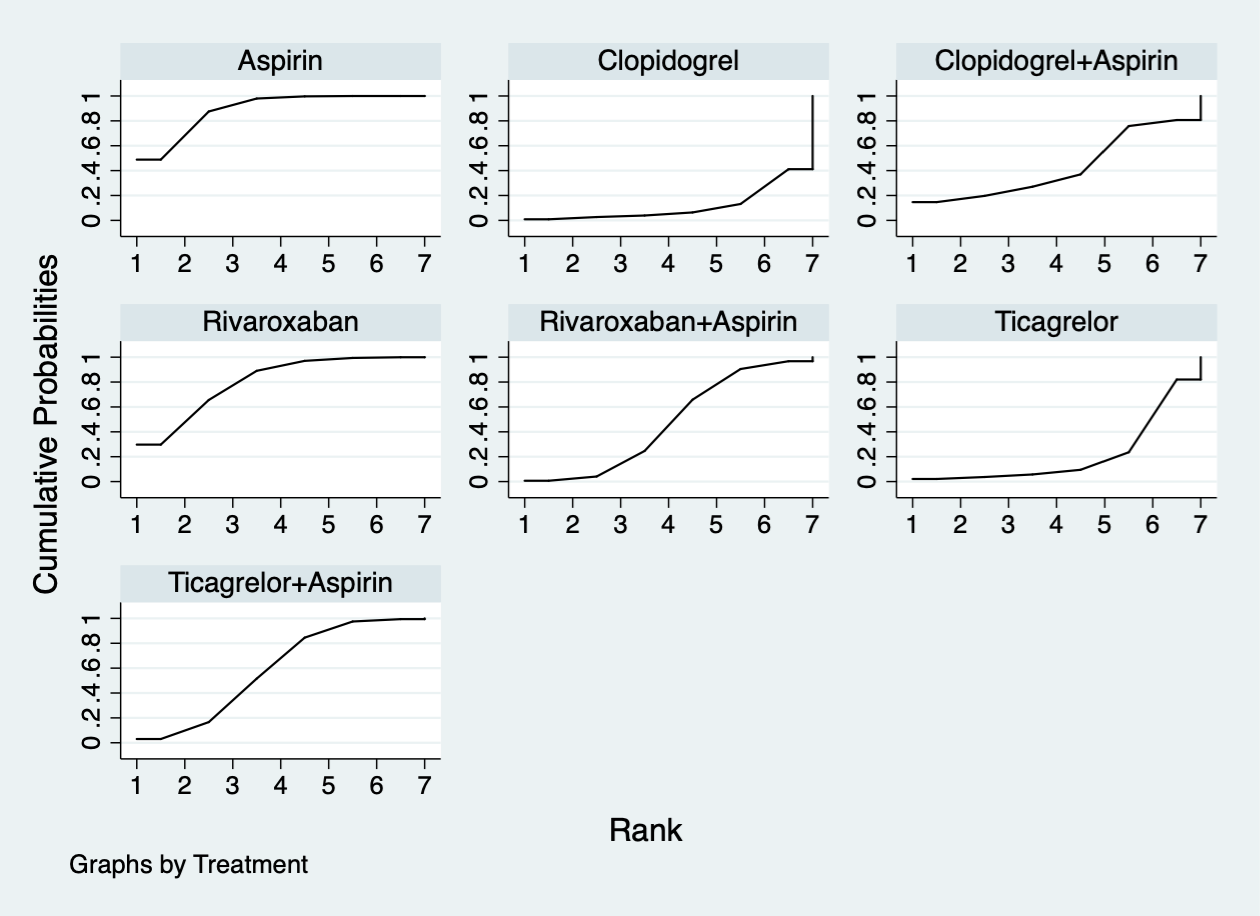


| Drug | Aspirin | Rivaroxaban | Rivaroxaban+Aspirin | Ticagrelor+Aspirin | Clopidogrel+Aspirin | Ticagrelor | Clopidogrel |
| --- | --- | --- | --- | --- | --- | --- | --- |
| Rank 1 | 0.488 | 0.297 | 0.007 | 0.03 | 0.147 | 0.021 | 0.009 |
| Rank 2 | 0.387 | 0.359 | 0.034 | 0.136 | 0.05 | 0.016 | 0.018 |
| Rank 3 | 0.104 | 0.235 | 0.205 | 0.35 | 0.074 | 0.021 | 0.012 |
| Rank 4 | 0.017 | 0.08 | 0.412 | 0.329 | 0.099 | 0.038 | 0.025 |
| Rank 5 | 0.003 | 0.023 | 0.246 | 0.13 | 0.389 | 0.141 | 0.068 |
| Rank 6 | 0 | 0.006 | 0.063 | 0.019 | 0.048 | 0.584 | 0.279 |
| Rank 7 | 0 | 0 | 0.032 | 0.006 | 0.193 | 0.18 | 0.588 |
| MeanRank | 1.657 | 2.191 | 4.17 | 3.474 | 4.449 | 5.737 | 6.311 |
| SUCRA | 89 | 80.1 | 47.1 | 58.8 | 42.5 | 21.1 | 11.4 |

1. Myocardial infarction for main analysis in the total cohort


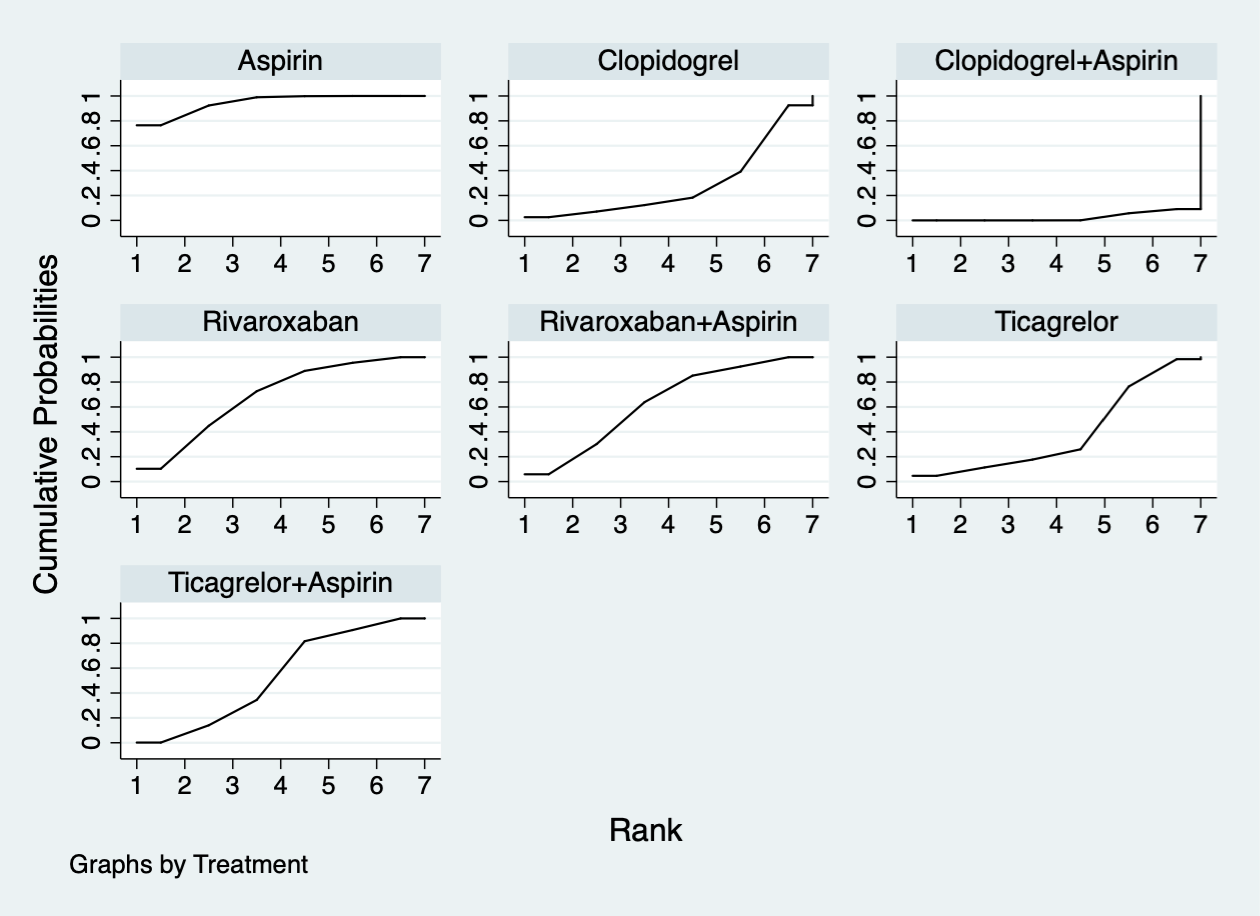


| Drug | Aspirin | Rivaroxaban | Rivaroxaban+Aspirin | Ticagrelor+Aspirin | Clopidogrel+Aspirin | Ticagrelor | Clopidogrel |
| --- | --- | --- | --- | --- | --- | --- | --- |
| Rank 1 | 0.764 | 0.103 | 0.059 | 0.002 | 0 | 0.046 | 0.026 |
| Rank 2 | 0.159 | 0.346 | 0.243 | 0.139 | 0 | 0.067 | 0.046 |
| Rank 3 | 0.066 | 0.278 | 0.337 | 0.204 | 0 | 0.063 | 0.052 |
| Rank 4 | 0.009 | 0.163 | 0.213 | 0.472 | 0.001 | 0.082 | 0.06 |
| Rank 5 | 0.001 | 0.065 | 0.073 | 0.09 | 0.057 | 0.505 | 0.209 |
| Rank 6 | 0 | 0.045 | 0.075 | 0.094 | 0.033 | 0.22 | 0.533 |
| Rank 7 | 0 | 0 | 0 | 0 | 0.909 | 0.016 | 0.075 |
| MeanRank | 1.321 | 2.876 | 3.223 | 3.794 | 6.85 | 4.654 | 5.282 |
| SUCRA | 94.6 | 68.7 | 62.9 | 53.5 | 2.5 | 39.1 | 28.7 |

1. Ischemic stroke for main analysis in the total cohort


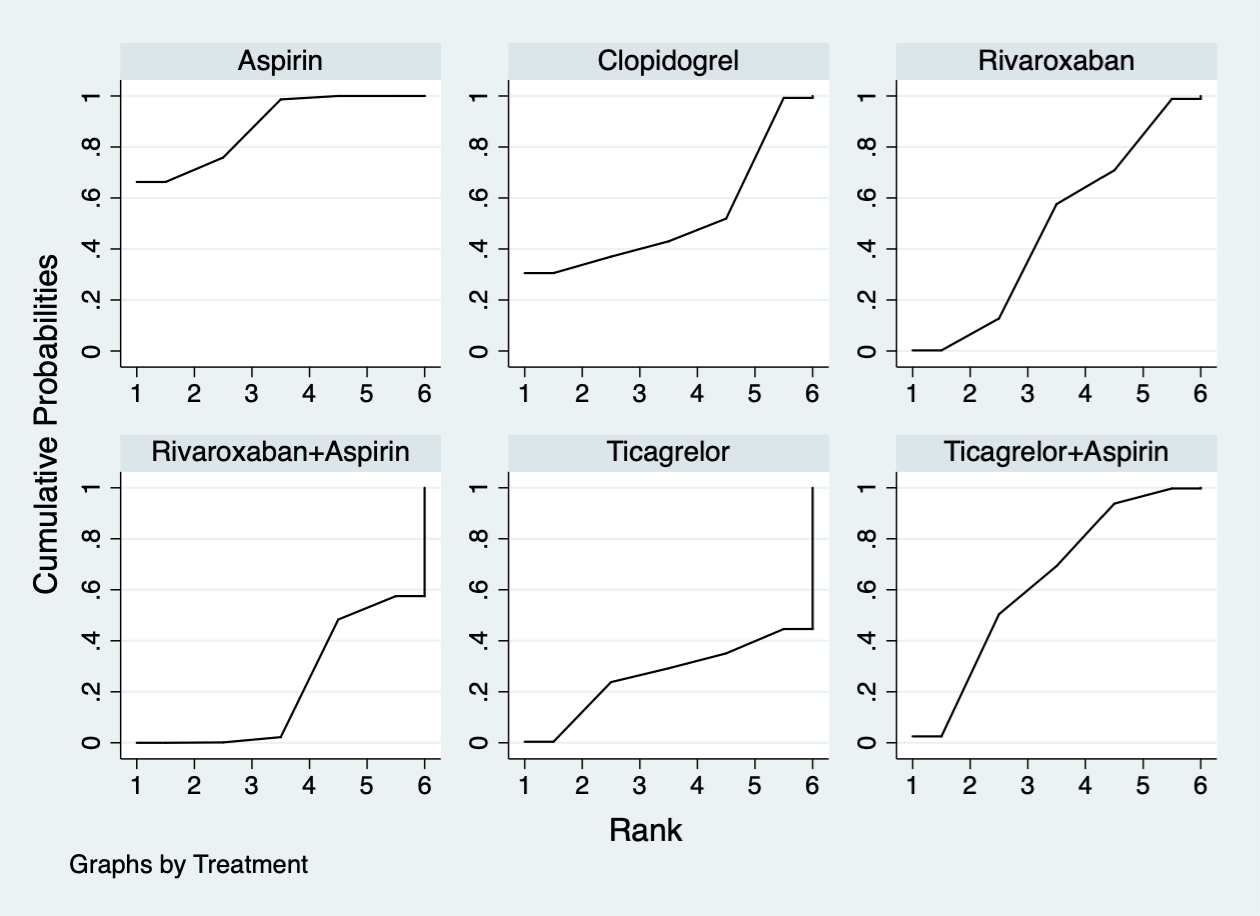


| Drug | Aspirin | Rivaroxaban | Rivaroxaban+Aspirin | Ticagrelor+Aspirin | Ticagrelor | Clopidogrel |
| --- | --- | --- | --- | --- | --- | --- |
| Rank 1 | 0.663 | 0.002 | 0 | 0.025 | 0.004 | 0.306 |
| Rank 2 | 0.096 | 0.125 | 0.001 | 0.479 | 0.234 | 0.064 |
| Rank 3 | 0.228 | 0.448 | 0.021 | 0.189 | 0.054 | 0.06 |
| Rank 4 | 0.014 | 0.133 | 0.461 | 0.245 | 0.058 | 0.089 |
| Rank 5 | 0 | 0.28 | 0.092 | 0.059 | 0.095 | 0.473 |
| Rank 6 | 0 | 0.011 | 0.425 | 0.003 | 0.554 | 0.008 |
| MeanRank | 1.595 | 3.594 | 4.919 | 2.843 | 4.665 | 3.383 |
| SUCRA | 88.2 | 48.1 | 21.6 | 63.2 | 26.6 | 52.3 |

1. Minor bleeding for main analysis in the total cohort


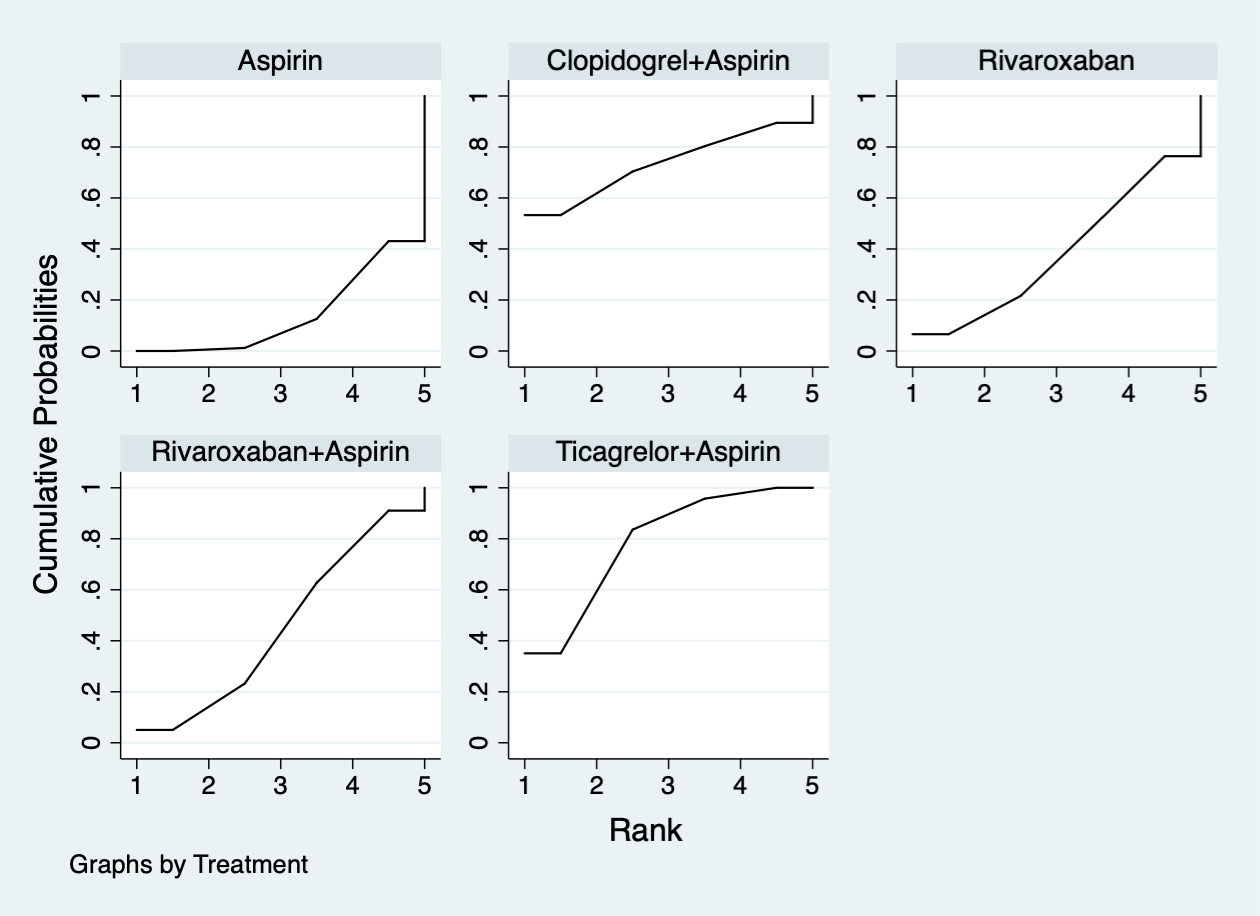


| Drug | Aspirin | Rivaroxaban | Rivaroxaban+Aspirin | Ticagrelor+Aspirin | Clopidogrel+Aspirin |
| --- | --- | --- | --- | --- | --- |
| Rank 1 | 0 | 0.066 | 0.05 | 0.351 | 0.533 |
| Rank 2 | 0.012 | 0.15 | 0.182 | 0.485 | 0.171 |
| Rank 3 | 0.114 | 0.271 | 0.395 | 0.121 | 0.099 |
| Rank 4 | 0.305 | 0.278 | 0.283 | 0.043 | 0.092 |
| Rank 5 | 0.569 | 0.236 | 0.089 | 0 | 0.105 |
| MeanRank | 4.431 | 3.471 | 3.176 | 1.856 | 2.065 |
| SUCRA | 14.2 | 38.3 | 45.5 | 78.6 | 73.4 |

1. Intracranial hemorrhage for main analysis in the total cohort


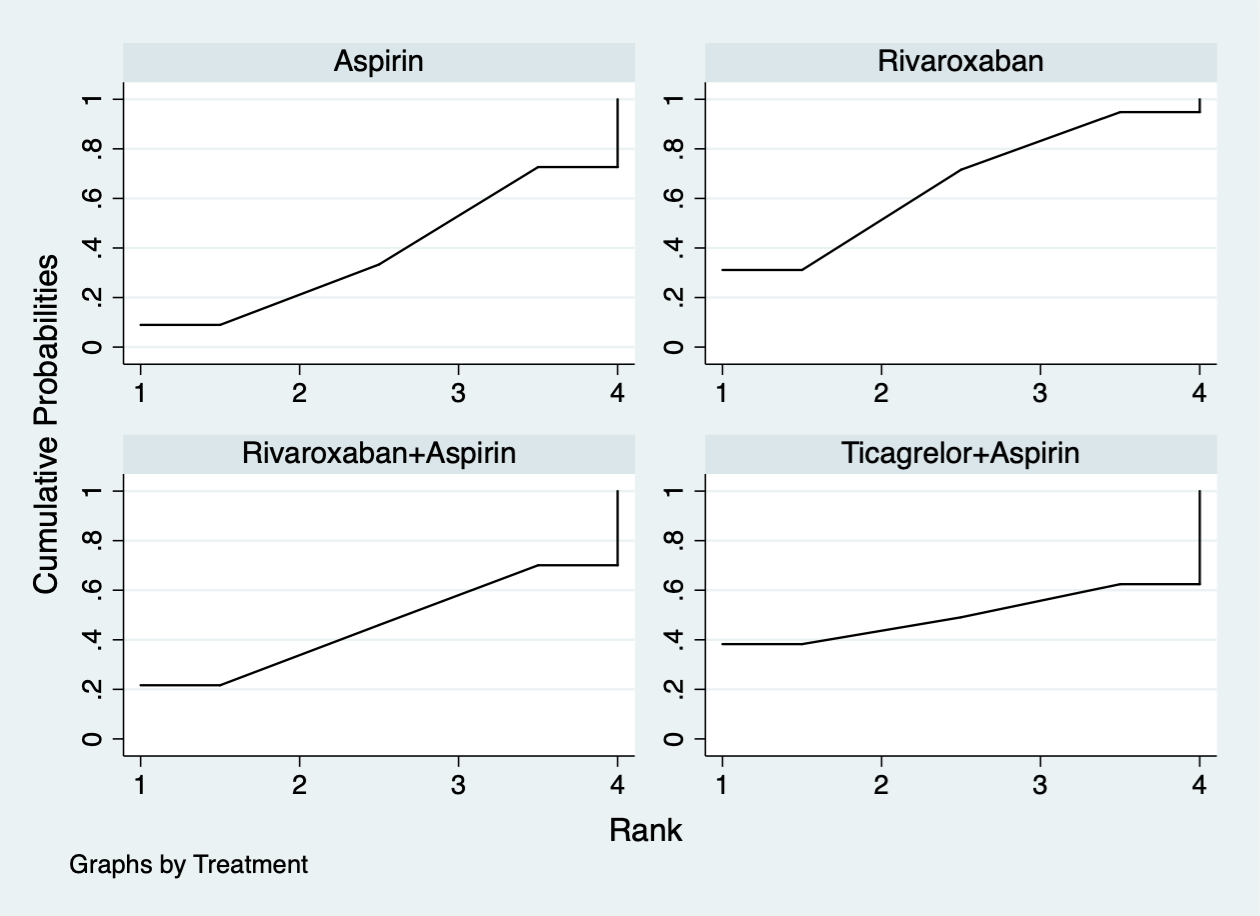


| Drug | Aspirin | Rivaroxaban | Rivaroxaban+Aspirin | Ticagrelor+Aspirin |
| --- | --- | --- | --- | --- |
| Rank 1 | 0.09 | 0.311 | 0.217 | 0.383 |
| Rank 2 | 0.244 | 0.405 | 0.243 | 0.108 |
| Rank 3 | 0.393 | 0.233 | 0.241 | 0.134 |
| Rank 4 | 0.274 | 0.052 | 0.299 | 0.376 |
| MeanRank | 2.853 | 2.028 | 2.622 | 2.505 |
| SUCRA | 38.3 | 65.8 | 45.9 | 49.9 |

1. Major adverse cardiovascular and cerebrovascular events for main analysis in patients with coronary artery disease


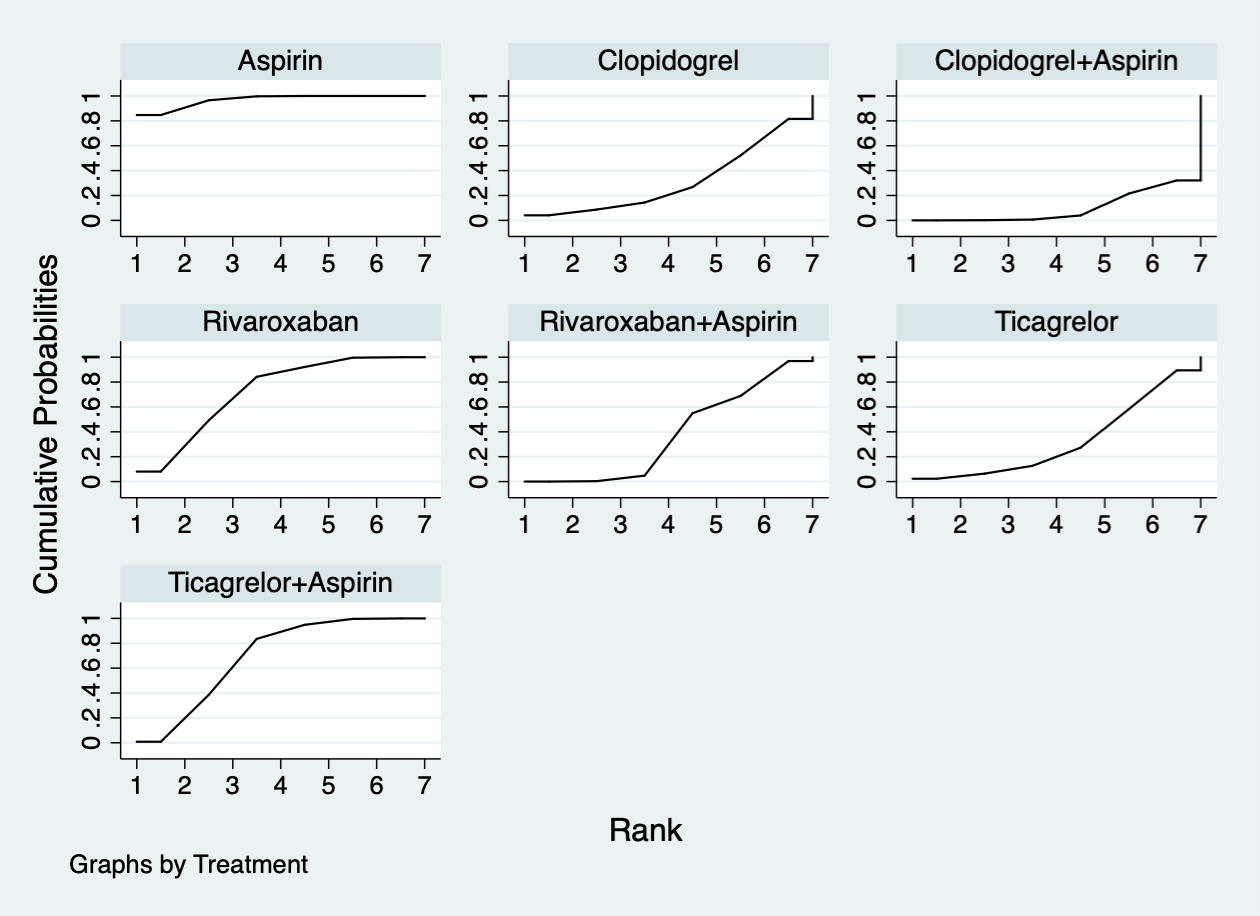


| Drug | Aspirin | Rivaroxaban | Rivaroxaban+Aspirin | Ticagrelor+Aspirin | Clopidogrel+Aspirin | Ticagrelor | Clopidogrel |
| --- | --- | --- | --- | --- | --- | --- | --- |
| Rank 1 | 0.847 | 0.08 | 0 | 0.009 | 0 | 0.023 | 0.041 |
| Rank 2 | 0.119 | 0.412 | 0.004 | 0.377 | 0.001 | 0.041 | 0.046 |
| Rank 3 | 0.032 | 0.35 | 0.044 | 0.449 | 0.005 | 0.063 | 0.057 |
| Rank 4 | 0.003 | 0.079 | 0.502 | 0.113 | 0.033 | 0.145 | 0.124 |
| Rank 5 | 0 | 0.074 | 0.139 | 0.047 | 0.176 | 0.31 | 0.254 |
| Rank 6 | 0 | 0.004 | 0.28 | 0.004 | 0.106 | 0.312 | 0.294 |
| Rank 7 | 0 | 0 | 0.031 | 0 | 0.679 | 0.106 | 0.184 |
| MeanRank | 1.193 | 2.664 | 4.74 | 2.821 | 6.418 | 5.038 | 5.122 |
| SUCRA | 96.8 | 72.2 | 37.7 | 69.6 | 9.7 | 32.7 | 31.3 |

1. Major adverse cardiovascular and cerebrovascular events for main analysis plus COMMANDER HF trial in patients with coronary artery disease


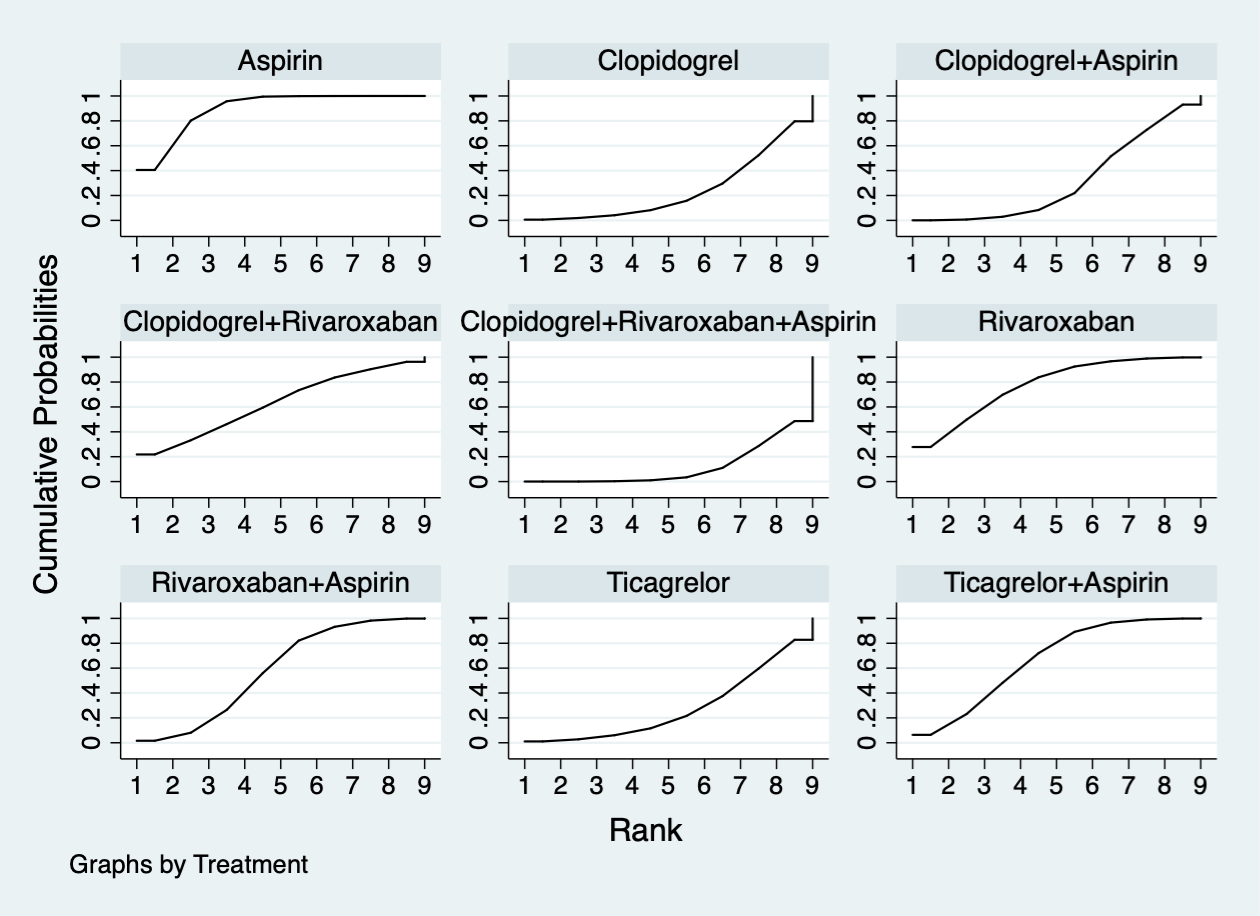


| Drug | Aspirin | Rivaroxaban | Rivaroxaban+Aspirin | Ticagrelor+Aspirin | Clopidogrel+Aspirin | Ticagrelor | Clopidogrel | Clopidogrel+Rivaroxaban | Clopidogrel+Rivaroxaban+Aspirin |
| --- | --- | --- | --- | --- | --- | --- | --- | --- | --- |
| Rank 1 | 0.405 | 0.278 | 0.017 | 0.064 | 0.001 | 0.011 | 0.006 | 0.218 | 0 |
| Rank 2 | 0.397 | 0.22 | 0.064 | 0.168 | 0.006 | 0.017 | 0.013 | 0.114 | 0 |
| Rank 3 | 0.156 | 0.2 | 0.184 | 0.251 | 0.022 | 0.033 | 0.022 | 0.13 | 0.003 |
| Rank 4 | 0.037 | 0.14 | 0.295 | 0.238 | 0.054 | 0.055 | 0.041 | 0.132 | 0.007 |
| Rank 5 | 0.004 | 0.087 | 0.262 | 0.171 | 0.136 | 0.1 | 0.076 | 0.14 | 0.024 |
| Rank 6 | 0.001 | 0.042 | 0.111 | 0.074 | 0.296 | 0.159 | 0.139 | 0.102 | 0.076 |
| Rank 7 | 0 | 0.022 | 0.05 | 0.025 | 0.213 | 0.221 | 0.227 | 0.067 | 0.176 |
| Rank 8 | 0 | 0.009 | 0.016 | 0.008 | 0.203 | 0.232 | 0.273 | 0.059 | 0.2 |
| Rank 9 | 0 | 0.002 | 0.001 | 0.001 | 0.069 | 0.172 | 0.203 | 0.037 | 0.514 |
| MeanRank | 1.841 | 2.809 | 4.34 | 3.652 | 6.487 | 6.769 | 7.076 | 3.95 | 8.071 |
| SUCRA | 89.5 | 77.4 | 58.2 | 66.8 | 31.4 | 27.9 | 24 | 63.1 | 11.6 |

1. Major bleeding for main analysis in patients with coronary artery disease


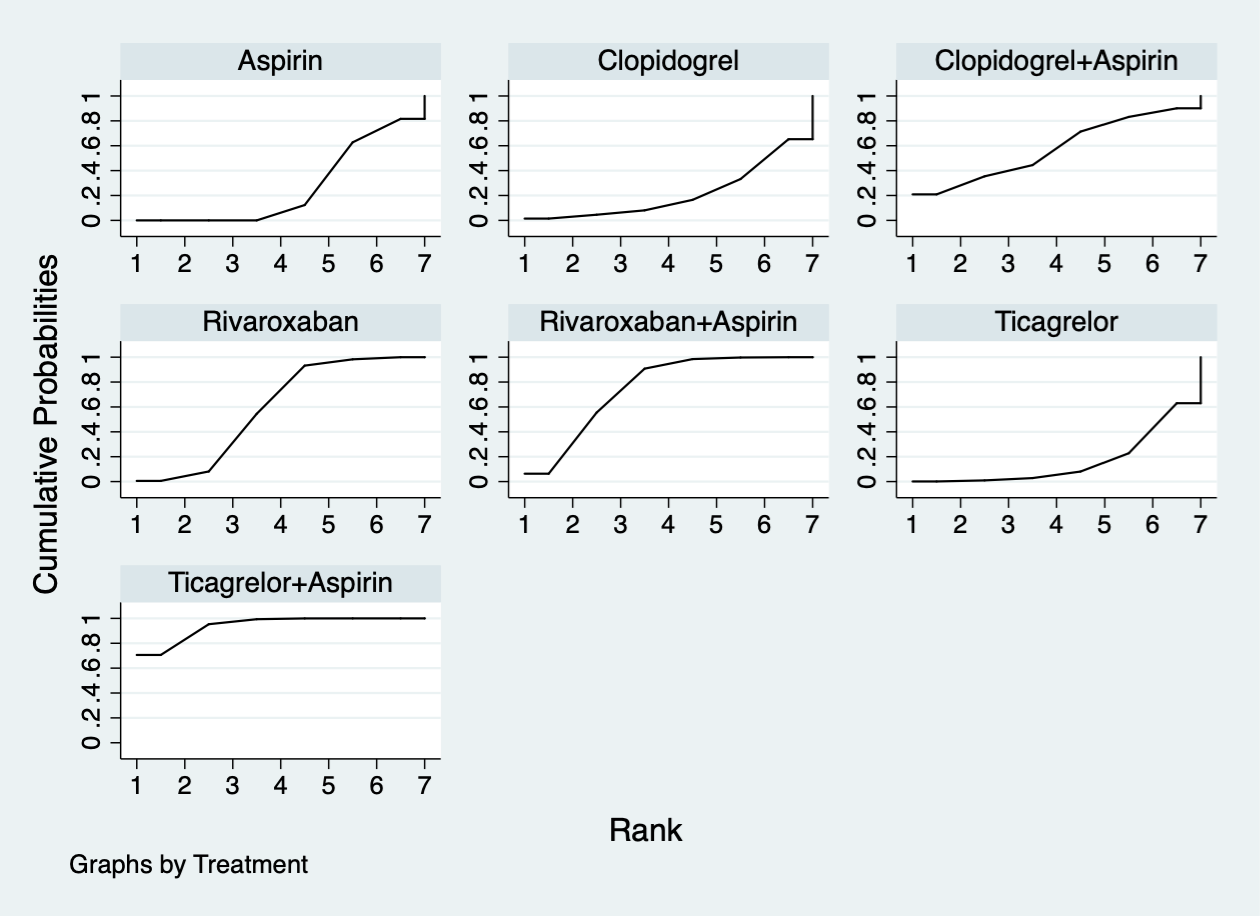


| Drug | Aspirin | Rivaroxaban | Rivaroxaban+Aspirin | Ticagrelor+Aspirin | Clopidogrel+Aspirin | Ticagrelor | Clopidogrel |
| --- | --- | --- | --- | --- | --- | --- | --- |
| Rank 1 | 0 | 0.006 | 0.063 | 0.706 | 0.209 | 0.001 | 0.015 |
| Rank 2 | 0 | 0.076 | 0.492 | 0.247 | 0.145 | 0.009 | 0.031 |
| Rank 3 | 0 | 0.464 | 0.353 | 0.04 | 0.09 | 0.019 | 0.035 |
| Rank 4 | 0.123 | 0.387 | 0.077 | 0.006 | 0.269 | 0.052 | 0.085 |
| Rank 5 | 0.504 | 0.05 | 0.013 | 0 | 0.118 | 0.147 | 0.167 |
| Rank 6 | 0.189 | 0.017 | 0.003 | 0 | 0.069 | 0.402 | 0.32 |
| Rank 7 | 0.184 | 0 | 0 | 0 | 0.099 | 0.37 | 0.347 |
| MeanRank | 5.434 | 3.45 | 2.497 | 1.344 | 3.542 | 6.021 | 5.706 |
| SUCRA | 26.1 | 59.1 | 75.1 | 94.2 | 57.6 | 16.3 | 21.5 |

1. Death from any cause for main analysis in patients with coronary artery disease


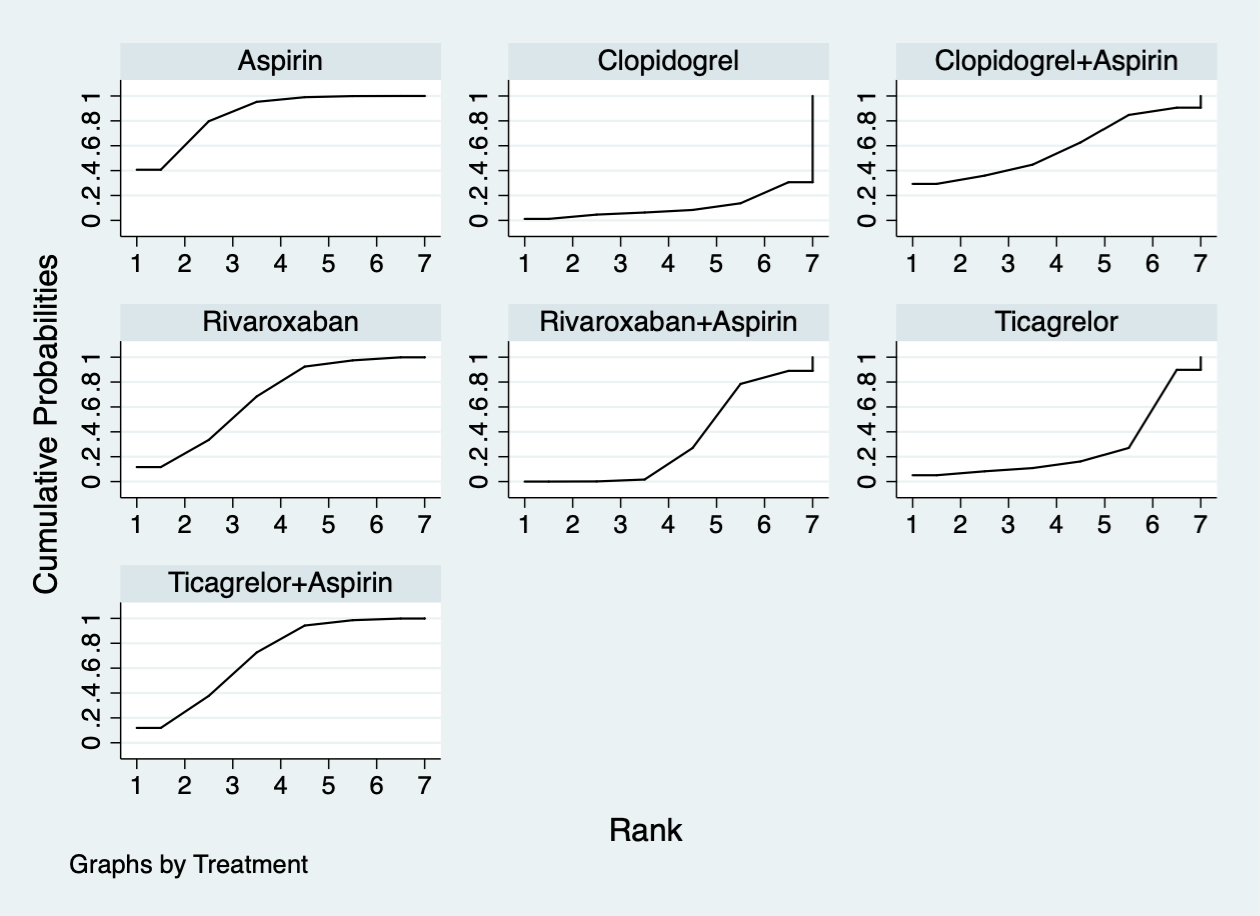


| Drug | Aspirin | Rivaroxaban | Rivaroxaban+Aspirin | Ticagrelor+Aspirin | Clopidogrel+Aspirin | Ticagrelor | Clopidogrel |
| --- | --- | --- | --- | --- | --- | --- | --- |
| Rank 1 | 0.407 | 0.117 | 0 | 0.12 | 0.293 | 0.051 | 0.012 |
| Rank 2 | 0.39 | 0.219 | 0.001 | 0.258 | 0.066 | 0.032 | 0.035 |
| Rank 3 | 0.156 | 0.349 | 0.015 | 0.349 | 0.088 | 0.026 | 0.017 |
| Rank 4 | 0.037 | 0.24 | 0.254 | 0.217 | 0.179 | 0.054 | 0.021 |
| Rank 5 | 0.009 | 0.05 | 0.515 | 0.043 | 0.221 | 0.109 | 0.054 |
| Rank 6 | 0.001 | 0.025 | 0.105 | 0.013 | 0.058 | 0.628 | 0.169 |
| Rank 7 | 0 | 0.001 | 0.11 | 0.001 | 0.094 | 0.101 | 0.693 |
| MeanRank | 1.854 | 2.969 | 5.038 | 2.851 | 3.516 | 5.429 | 6.352 |
| SUCRA | 85.8 | 67.2 | 32.7 | 69.2 | 58 | 26.2 | 10.8 |

1. Cardiovascular death for main analysis in patients with coronary artery disease


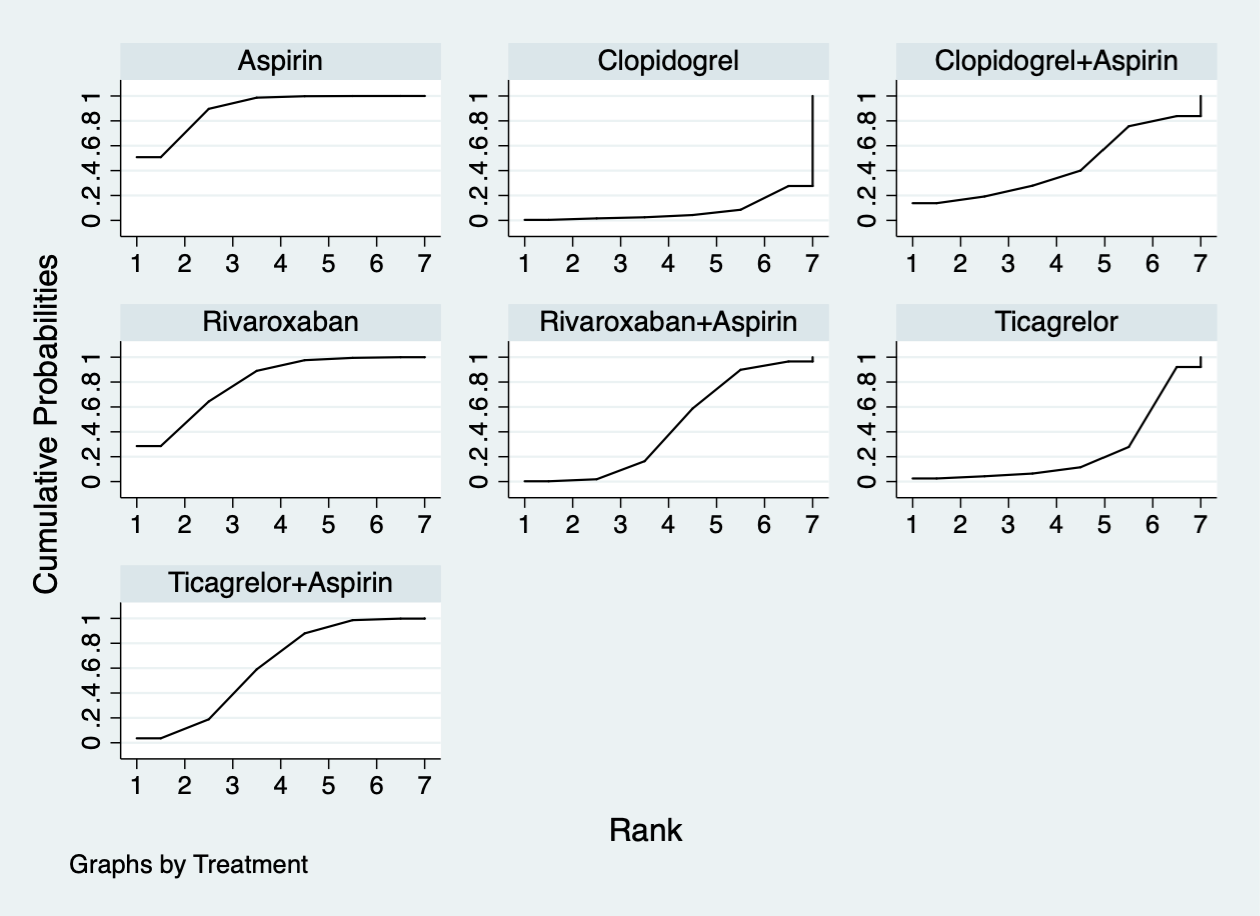


| Drug | Aspirin | Rivaroxaban | Rivaroxaban+Aspirin | Ticagrelor+Aspirin | Clopidogrel+Aspirin | Ticagrelor | Clopidogrel |
| --- | --- | --- | --- | --- | --- | --- | --- |
| Rank 1 | 0.508 | 0.286 | 0.003 | 0.036 | 0.138 | 0.025 | 0.004 |
| Rank 2 | 0.389 | 0.358 | 0.016 | 0.153 | 0.054 | 0.018 | 0.012 |
| Rank 3 | 0.09 | 0.246 | 0.145 | 0.401 | 0.087 | 0.022 | 0.009 |
| Rank 4 | 0.012 | 0.086 | 0.423 | 0.289 | 0.121 | 0.05 | 0.018 |
| Rank 5 | 0.002 | 0.019 | 0.311 | 0.106 | 0.356 | 0.163 | 0.043 |
| Rank 6 | 0 | 0.005 | 0.067 | 0.012 | 0.082 | 0.643 | 0.191 |
| Rank 7 | 0 | 0 | 0.034 | 0.002 | 0.162 | 0.079 | 0.723 |
| MeanRank | 1.614 | 2.209 | 4.357 | 3.317 | 4.397 | 5.553 | 6.549 |
| SUCRA | 89.8 | 79.8 | 44 | 61.3 | 43.4 | 24.1 | 7.5 |

1. Major adverse cardiovascular and cerebrovascular events for main analysis in patients with peripheral artery disease


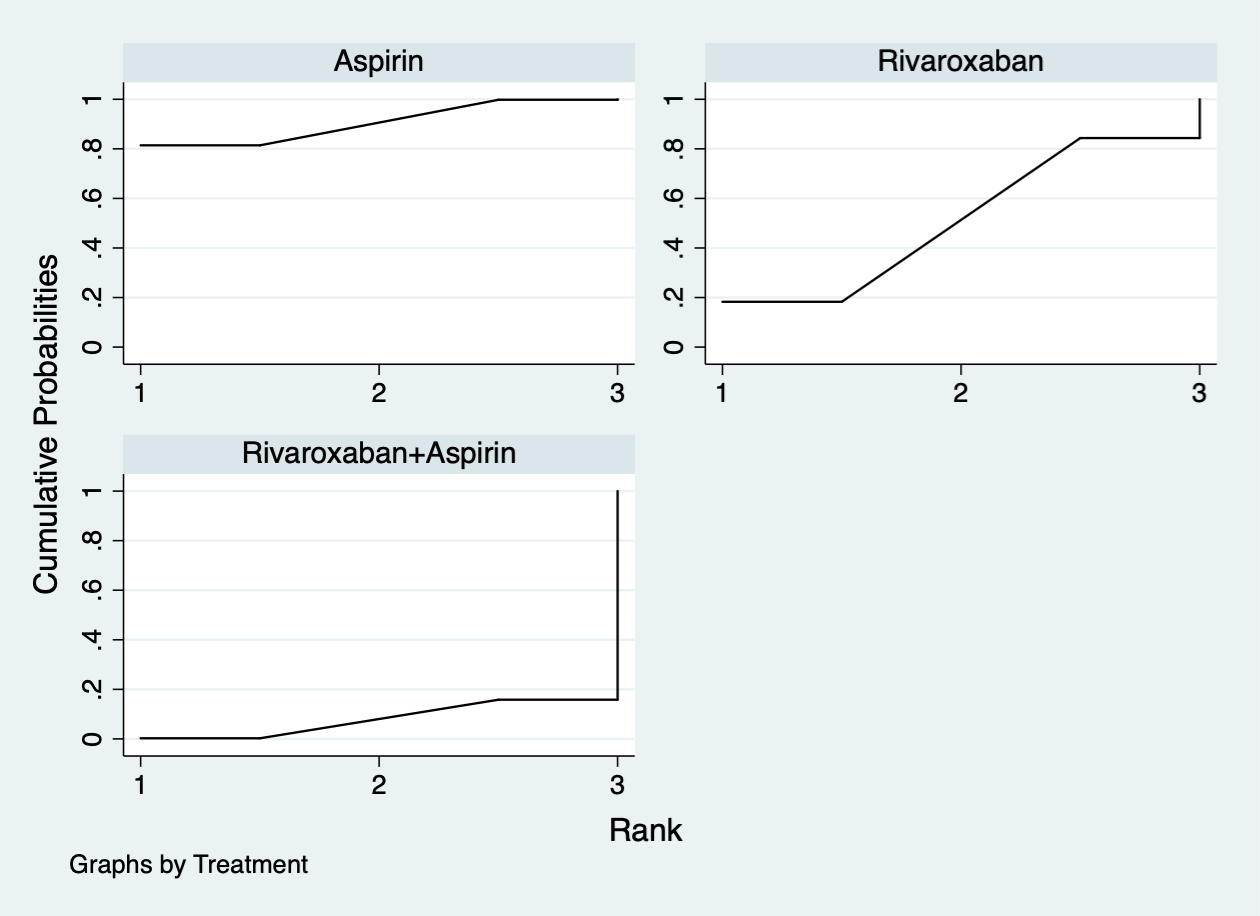


| Drug | Aspirin | Rivaroxaban | Rivaroxaban+Aspirin |
| --- | --- | --- | --- |
| Rank 1 | 0.814 | 0.183 | 0.003 |
| Rank 2 | 0.184 | 0.661 | 0.156 |
| Rank 3 | 0.002 | 0.156 | 0.842 |
| MeanRank | 1.188 | 1.973 | 2.841 |
| SUCRA | 90.6 | 51.3 | 8.1 |

1. Major adverse cardiovascular and cerebrovascular events for main analysis plus CAPRIE trial in patients with peripheral artery disease


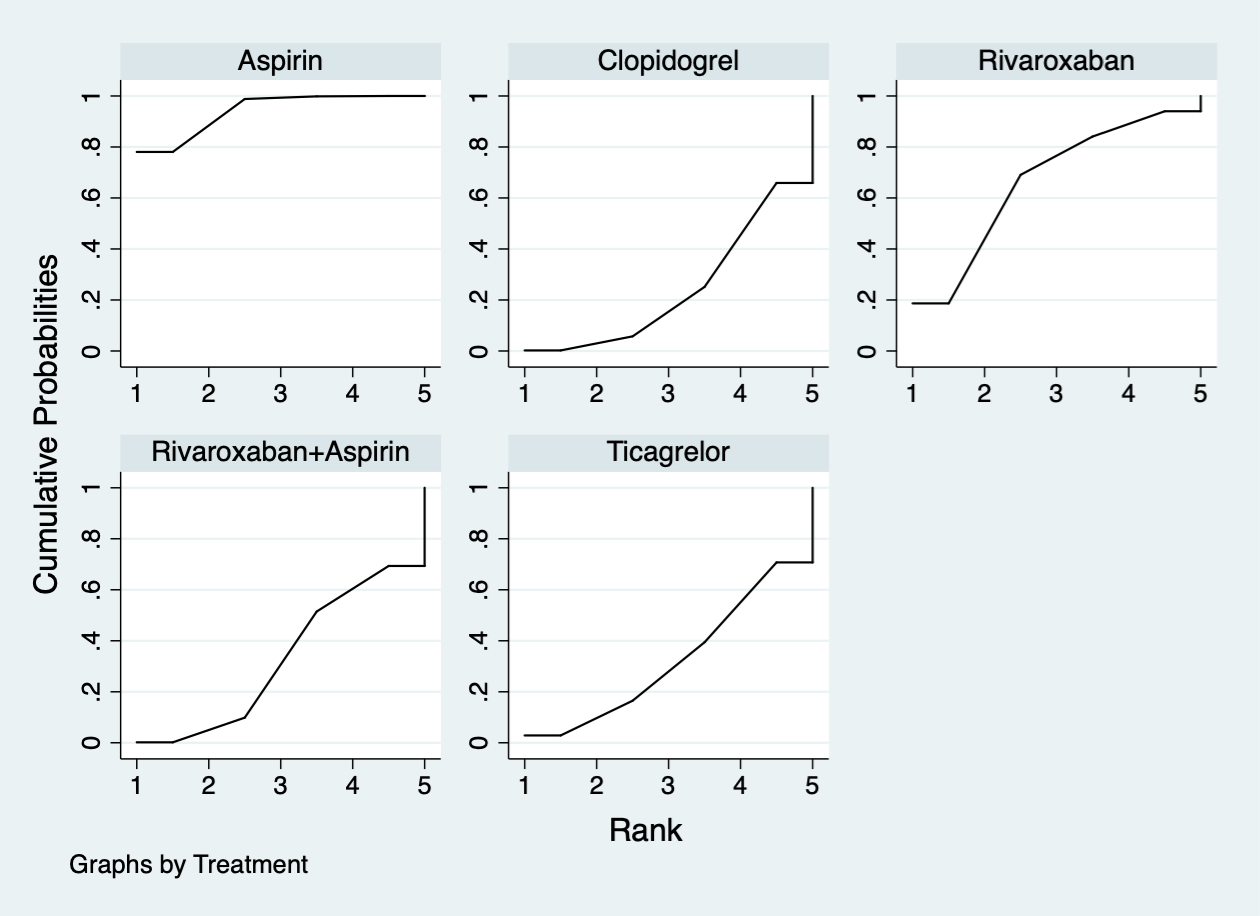


| Drug | Aspirin | Rivaroxaban | Rivaroxaban+Aspirin | Ticagrelor | Clopidogrel |
| --- | --- | --- | --- | --- | --- |
| Rank 1 | 0.781 | 0.187 | 0.002 | 0.029 | 0.002 |
| Rank 2 | 0.208 | 0.504 | 0.097 | 0.136 | 0.055 |
| Rank 3 | 0.01 | 0.15 | 0.416 | 0.229 | 0.194 |
| Rank 4 | 0.001 | 0.099 | 0.179 | 0.313 | 0.408 |
| Rank 5 | 0 | 0.06 | 0.307 | 0.293 | 0.341 |
| MeanRank | 1.231 | 2.341 | 3.695 | 3.705 | 4.031 |
| SUCRA | 94.2 | 66.5 | 32.7 | 32.4 | 24.2 |

1. Major adverse cardiovascular and cerebrovascular events for main analysis plus DAVID trial in patients with peripheral artery disease


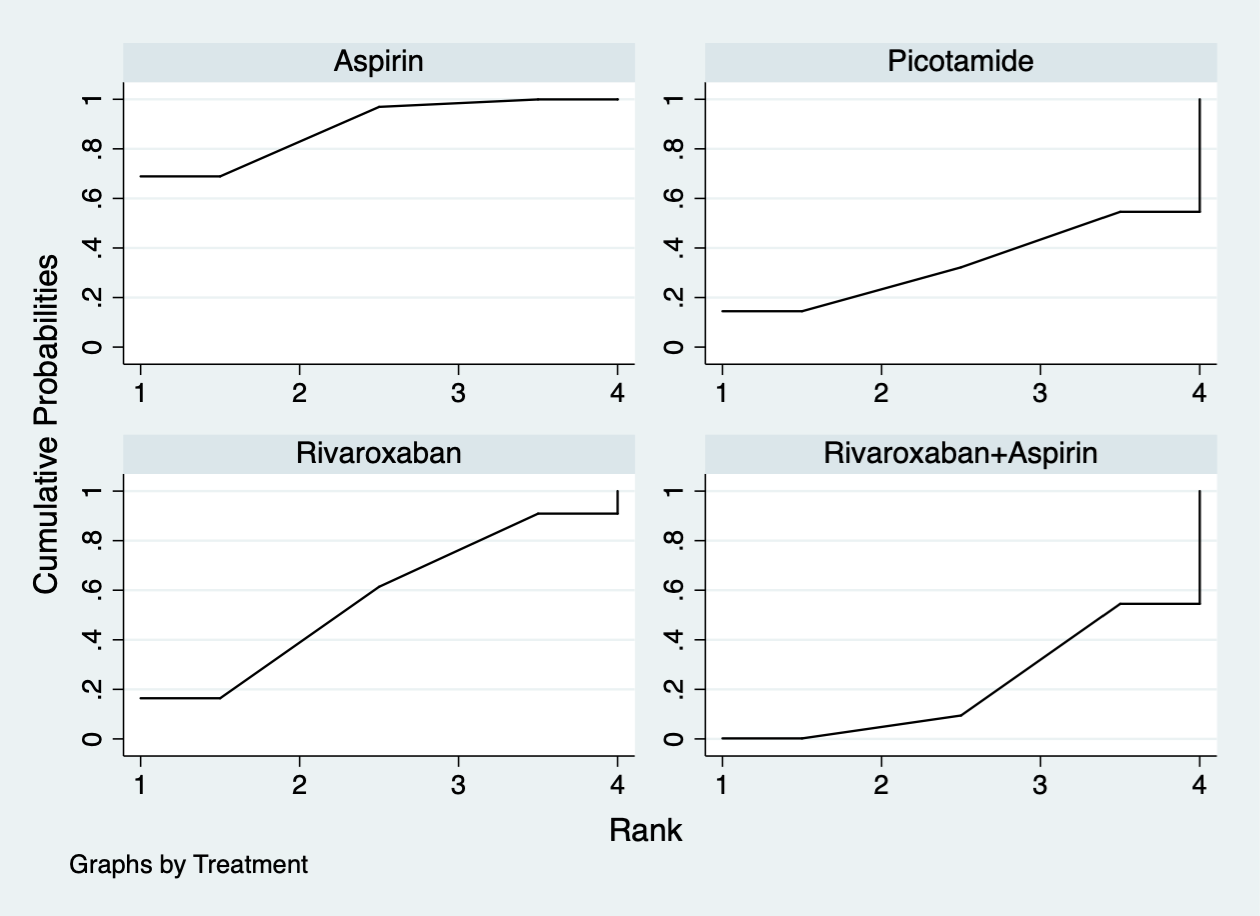


| Drug | Aspirin | Rivaroxaban | Rivaroxaban+Aspirin | Picotamide |
| --- | --- | --- | --- | --- |
| Rank 1 | 0.689 | 0.164 | 0.002 | 0.145 |
| Rank 2 | 0.281 | 0.45 | 0.092 | 0.177 |
| Rank 3 | 0.03 | 0.295 | 0.451 | 0.224 |
| Rank 4 | 0 | 0.091 | 0.455 | 0.454 |
| MeanRank | 1.341 | 2.313 | 3.359 | 2.987 |
| SUCRA | 88.6 | 56.2 | 21.4 | 33.8 |

1. Major adverse cardiovascular and cerebrovascular events for main analysis plus CAPRIE and DAVID trials in patients with peripheral artery disease


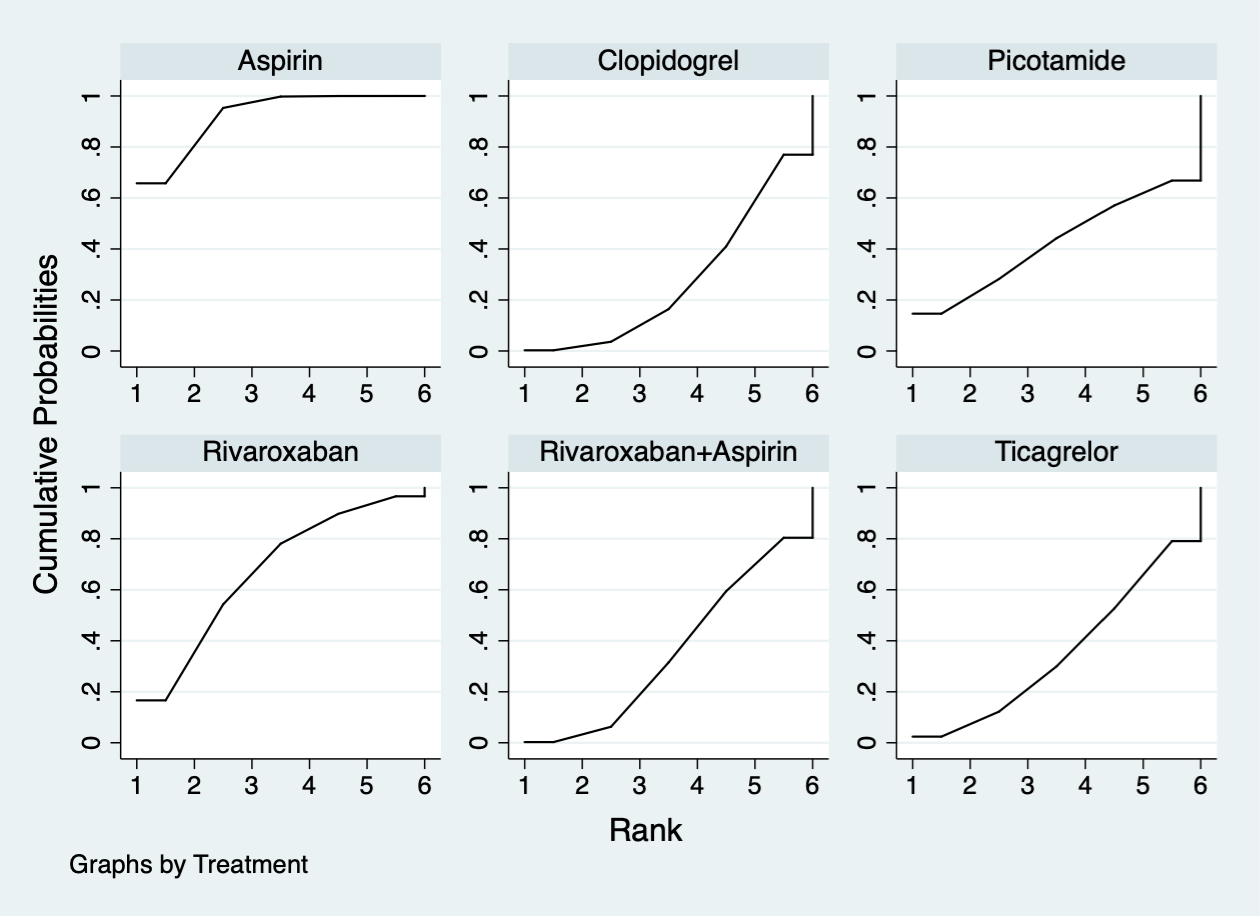


| Drug | Aspirin | Rivaroxaban | Rivaroxaban+Aspirin | Ticagrelor | Clopidogrel | Picotamide |
| --- | --- | --- | --- | --- | --- | --- |
| Rank 1 | 0.657 | 0.166 | 0.003 | 0.024 | 0.003 | 0.146 |
| Rank 2 | 0.296 | 0.377 | 0.06 | 0.098 | 0.034 | 0.136 |
| Rank 3 | 0.045 | 0.238 | 0.252 | 0.177 | 0.128 | 0.16 |
| Rank 4 | 0.002 | 0.117 | 0.279 | 0.227 | 0.246 | 0.128 |
| Rank 5 | 0 | 0.069 | 0.21 | 0.264 | 0.36 | 0.098 |
| Rank 6 | 0 | 0.033 | 0.196 | 0.209 | 0.23 | 0.332 |
| MeanRank | 1.392 | 2.645 | 4.221 | 4.233 | 4.619 | 3.892 |
| SUCRA | 92.2 | 67.1 | 35.6 | 35.3 | 27.7 | 42.2 |

1. Major bleeding for main analysis in patients with peripheral artery disease


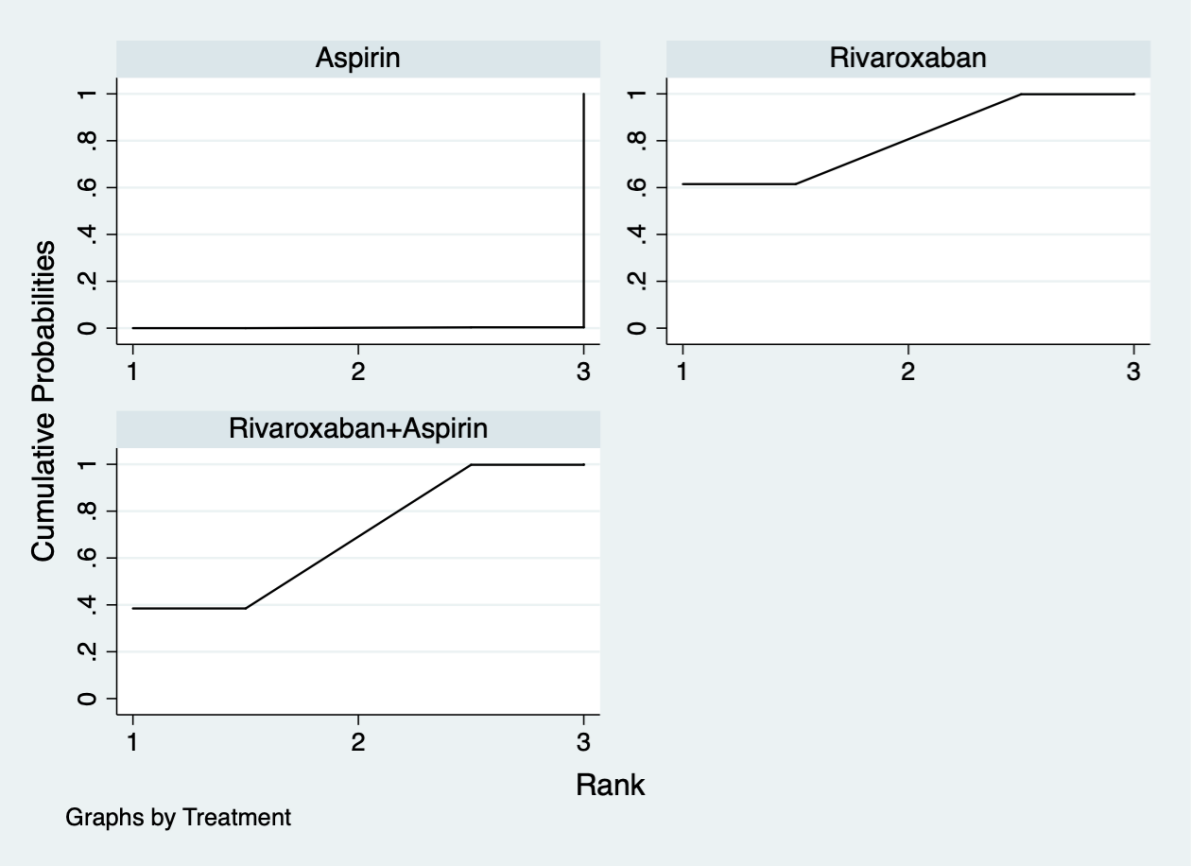


| Drug | Aspirin | Rivaroxaban | Rivaroxaban+Aspirin |
| --- | --- | --- | --- |
| Rank 1 | 0 | 0.615 | 0.385 |
| Rank 2 | 0.003 | 0.383 | 0.613 |
| Rank 3 | 0.996 | 0.002 | 0.002 |
| MeanRank | 2.994 | 1.387 | 1.617 |
| SUCRA | 0.2 | 80.7 | 69.1 |

1. Major adverse cardiovascular and cerebrovascular events for main analysis (without DAPT subgroup) plus the whole cohort of DAPT trial in total cohort


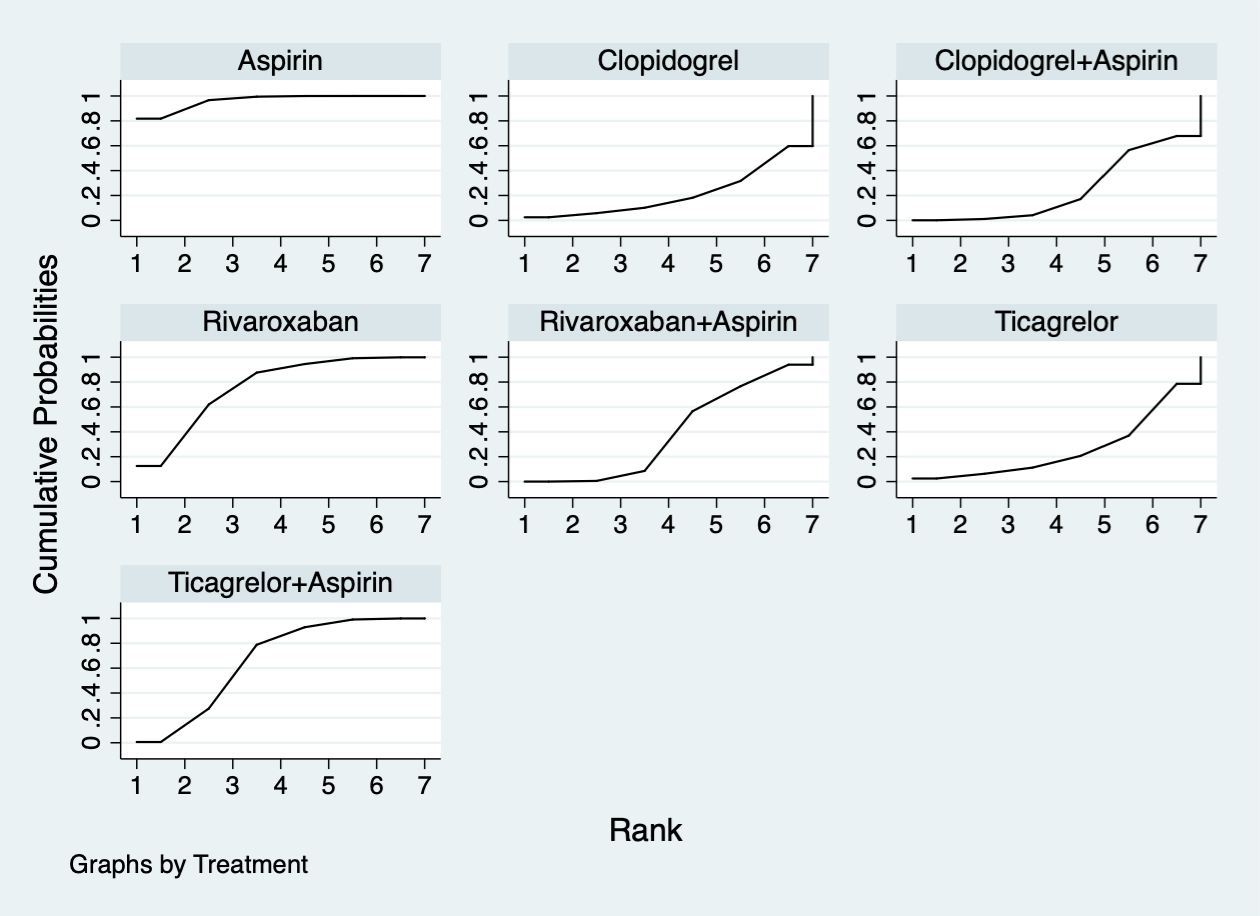


| Drug | Aspirin | Rivaroxaban | Rivaroxaban+Aspirin | Ticagrelor+Aspirin | Clopidogrel+Aspirin | Ticagrelor | Clopidogrel |
| --- | --- | --- | --- | --- | --- | --- | --- |
| Rank 1 | 0.818 | 0.125 | 0 | 0.006 | 0.001 | 0.025 | 0.025 |
| Rank 2 | 0.149 | 0.494 | 0.006 | 0.269 | 0.011 | 0.038 | 0.033 |
| Rank 3 | 0.028 | 0.256 | 0.08 | 0.513 | 0.03 | 0.049 | 0.043 |
| Rank 4 | 0.005 | 0.069 | 0.48 | 0.141 | 0.13 | 0.094 | 0.08 |
| Rank 5 | 0 | 0.046 | 0.201 | 0.062 | 0.393 | 0.163 | 0.135 |
| Rank 6 | 0 | 0.008 | 0.173 | 0.008 | 0.114 | 0.416 | 0.28 |
| Rank 7 | 0 | 0.001 | 0.06 | 0.001 | 0.322 | 0.214 | 0.403 |
| MeanRank | 1.22 | 2.442 | 4.635 | 3.012 | 5.536 | 5.433 | 5.716 |
| SUCRA | 96.3 | 75.9 | 39.4 | 66.5 | 24.5 | 26.1 | 21.3 |

1. Major bleeding for main analysis (without DAPT subgroup) plus the whole cohort of DAPT trial in total cohort


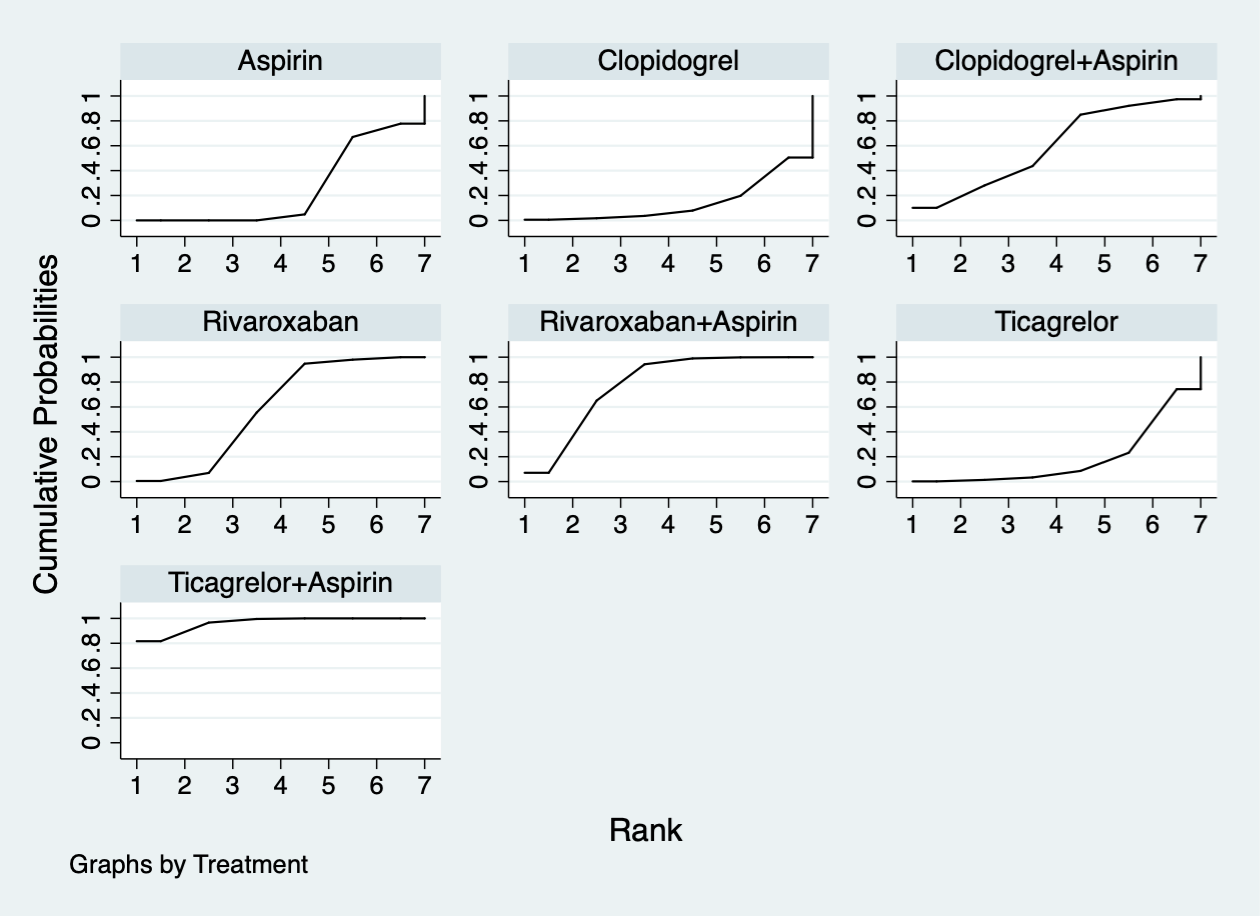


| Drug | Aspirin | Rivaroxaban | Rivaroxaban+Aspirin | Ticagrelor+Aspirin | Clopidogrel+Aspirin | Ticagrelor | Clopidogrel |
| --- | --- | --- | --- | --- | --- | --- | --- |
| Rank 1 | 0 | 0.005 | 0.071 | 0.817 | 0.101 | 0.002 | 0.005 |
| Rank 2 | 0 | 0.065 | 0.581 | 0.15 | 0.181 | 0.012 | 0.012 |
| Rank 3 | 0 | 0.485 | 0.292 | 0.029 | 0.155 | 0.02 | 0.019 |
| Rank 4 | 0.048 | 0.392 | 0.047 | 0.005 | 0.413 | 0.052 | 0.043 |
| Rank 5 | 0.622 | 0.032 | 0.009 | 0 | 0.071 | 0.146 | 0.119 |
| Rank 6 | 0.107 | 0.02 | 0.001 | 0 | 0.053 | 0.511 | 0.308 |
| Rank 7 | 0.222 | 0 | 0 | 0 | 0.026 | 0.257 | 0.495 |
| MeanRank | 5.498 | 3.438 | 2.348 | 1.224 | 3.435 | 5.889 | 6.166 |
| SUCRA | 24.9 | 59.3 | 77.6 | 96.3 | 59.4 | 18.5 | 14 |

1. Major adverse cardiovascular and cerebrovascular events for main analysis (without THEMIS subgroup) plus the whole cohort of THEMIS trial in total cohort


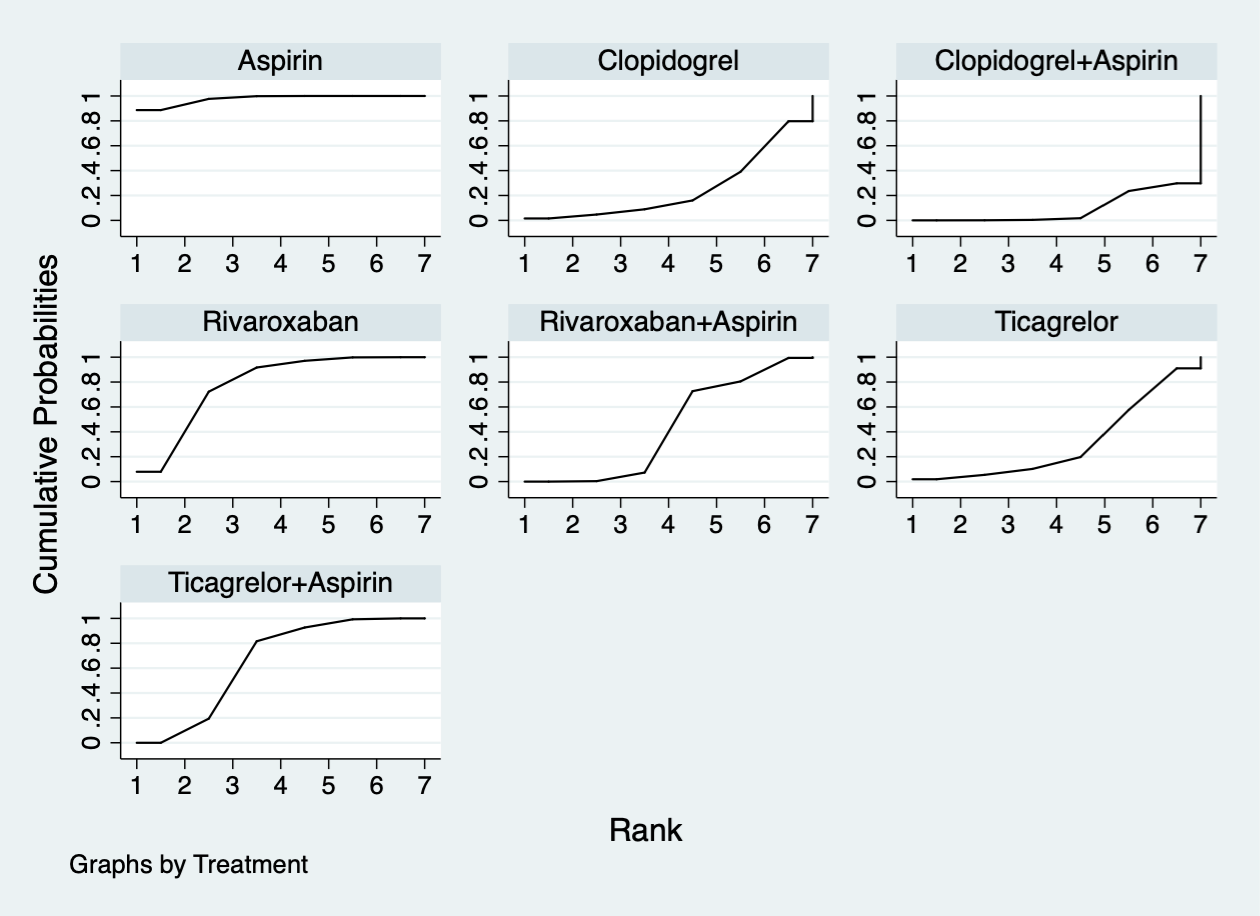


| Drug | Aspirin | Rivaroxaban | Rivaroxaban+Aspirin | Ticagrelor+Aspirin | Clopidogrel+Aspirin | Ticagrelor | Clopidogrel |
| --- | --- | --- | --- | --- | --- | --- | --- |
| Rank 1 | 0.886 | 0.079 | 0 | 0 | 0 | 0.019 | 0.015 |
| Rank 2 | 0.09 | 0.644 | 0.004 | 0.194 | 0.001 | 0.035 | 0.032 |
| Rank 3 | 0.022 | 0.195 | 0.069 | 0.622 | 0.003 | 0.048 | 0.042 |
| Rank 4 | 0.001 | 0.054 | 0.654 | 0.11 | 0.014 | 0.095 | 0.071 |
| Rank 5 | 0 | 0.028 | 0.078 | 0.066 | 0.218 | 0.379 | 0.231 |
| Rank 6 | 0 | 0.001 | 0.189 | 0.008 | 0.062 | 0.334 | 0.406 |
| Rank 7 | 0 | 0 | 0.006 | 0 | 0.702 | 0.09 | 0.203 |
| MeanRank | 1.136 | 2.314 | 4.397 | 3.072 | 6.443 | 5.142 | 5.501 |
| SUCRA | 97.7 | 78.1 | 43.4 | 65.5 | 9.3 | 31 | 25 |

1. Major bleeding for main analysis (without THEMIS subgroup) plus the whole cohort of THEMIS trial in total cohort


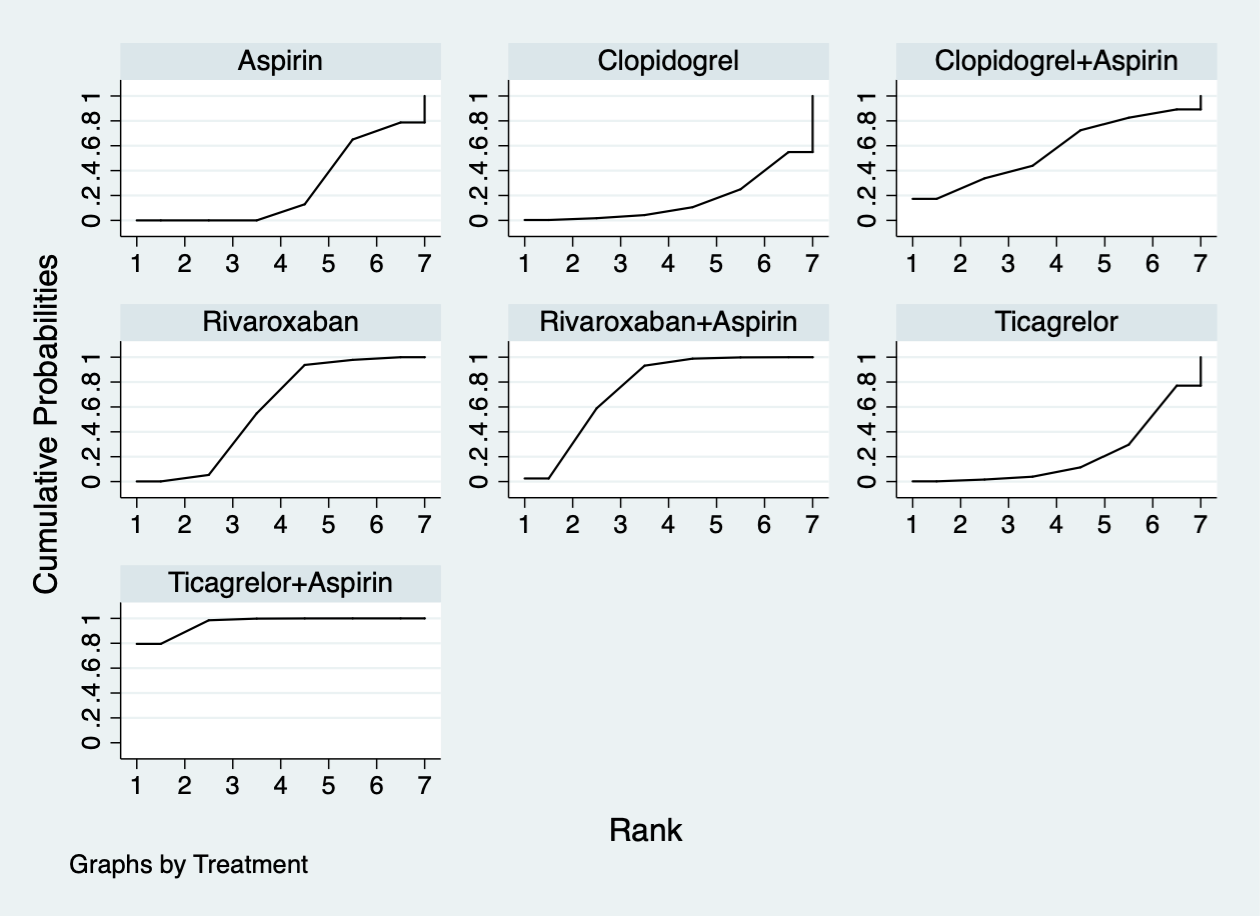


| Drug | Aspirin | Rivaroxaban | Rivaroxaban+Aspirin | Ticagrelor+Aspirin | Clopidogrel+Aspirin | Ticagrelor | Clopidogrel |
| --- | --- | --- | --- | --- | --- | --- | --- |
| Rank 1 | 0 | 0.001 | 0.025 | 0.795 | 0.173 | 0.002 | 0.003 |
| Rank 2 | 0 | 0.052 | 0.564 | 0.19 | 0.165 | 0.015 | 0.015 |
| Rank 3 | 0 | 0.495 | 0.343 | 0.013 | 0.101 | 0.023 | 0.025 |
| Rank 4 | 0.129 | 0.389 | 0.056 | 0.002 | 0.285 | 0.075 | 0.063 |
| Rank 5 | 0.521 | 0.041 | 0.01 | 0 | 0.101 | 0.183 | 0.144 |
| Rank 6 | 0.137 | 0.021 | 0.002 | 0 | 0.067 | 0.474 | 0.299 |
| Rank 7 | 0.213 | 0 | 0 | 0 | 0.108 | 0.229 | 0.451 |
| MeanRank | 5.434 | 3.477 | 2.468 | 1.222 | 3.609 | 5.763 | 6.031 |
| SUCRA | 26.1 | 58.7 | 75.5 | 96.3 | 56.5 | 20.7 | 16.2 |

1. Major adverse cardiovascular and cerebrovascular events for main analysis (without VOYAGER PAD subgroup) plus the whole cohort of VOYAGER PAD trial in total cohort


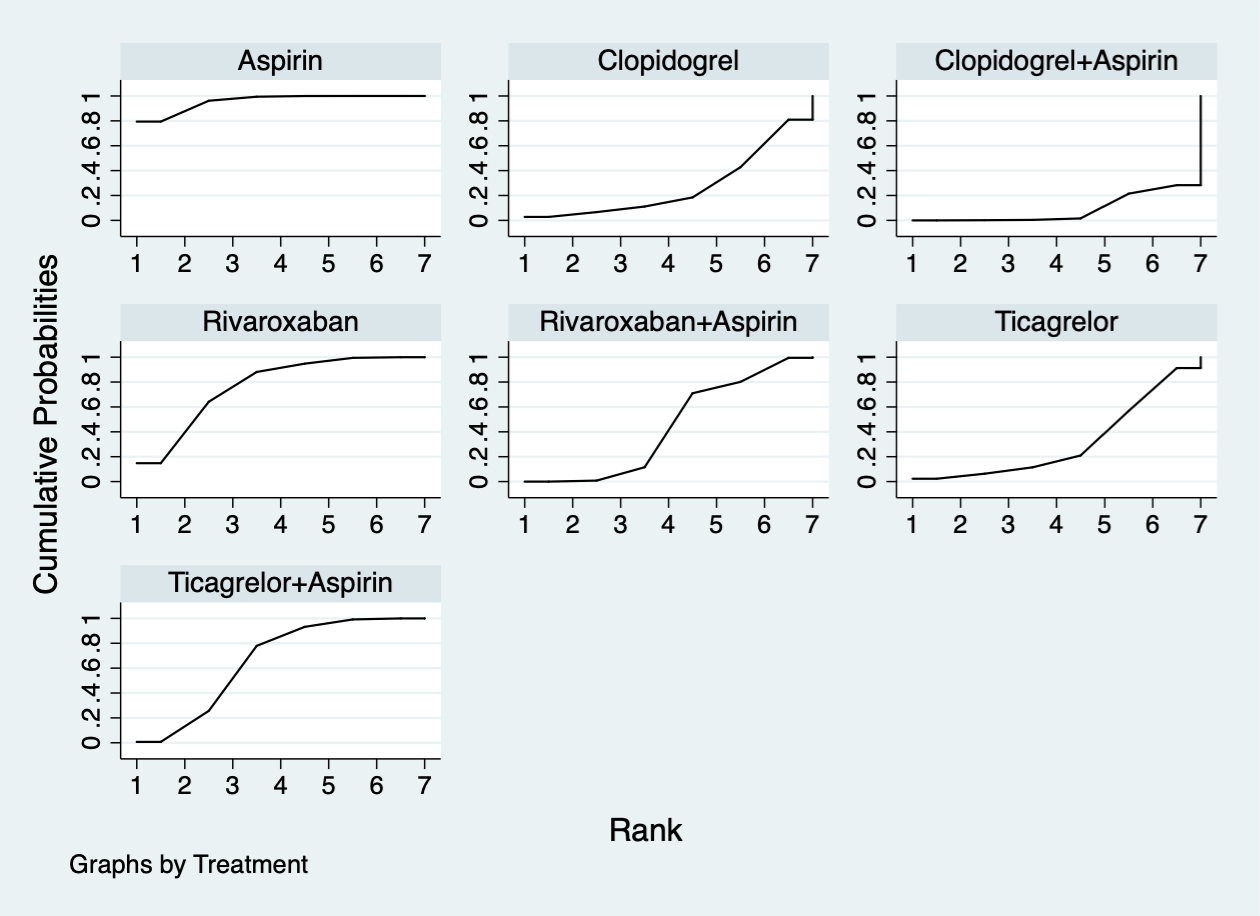


| Drug | Aspirin | Rivaroxaban | Rivaroxaban+Aspirin | Ticagrelor+Aspirin | Clopidogrel+Aspirin | Ticagrelor | Clopidogrel |
| --- | --- | --- | --- | --- | --- | --- | --- |
| Rank 1 | 0.794 | 0.148 | 0 | 0.007 | 0 | 0.023 | 0.028 |
| Rank 2 | 0.168 | 0.494 | 0.008 | 0.249 | 0.002 | 0.04 | 0.039 |
| Rank 3 | 0.032 | 0.24 | 0.107 | 0.522 | 0.003 | 0.051 | 0.045 |
| Rank 4 | 0.006 | 0.067 | 0.595 | 0.153 | 0.012 | 0.095 | 0.073 |
| Rank 5 | 0 | 0.047 | 0.092 | 0.059 | 0.199 | 0.359 | 0.243 |
| Rank 6 | 0 | 0.005 | 0.193 | 0.008 | 0.068 | 0.344 | 0.382 |
| Rank 7 | 0 | 0 | 0.005 | 0 | 0.717 | 0.087 | 0.191 |
| MeanRank | 1.25 | 2.389 | 4.37 | 3.026 | 6.483 | 5.104 | 5.377 |
| SUCRA | 95.8 | 76.9 | 43.8 | 66.1 | 8.7 | 31.5 | 27.1 |

1. Major bleeding for main analysis (without VOYAGER PAD subgroup) plus the whole cohort of VOYAGER PAD trial in total cohort


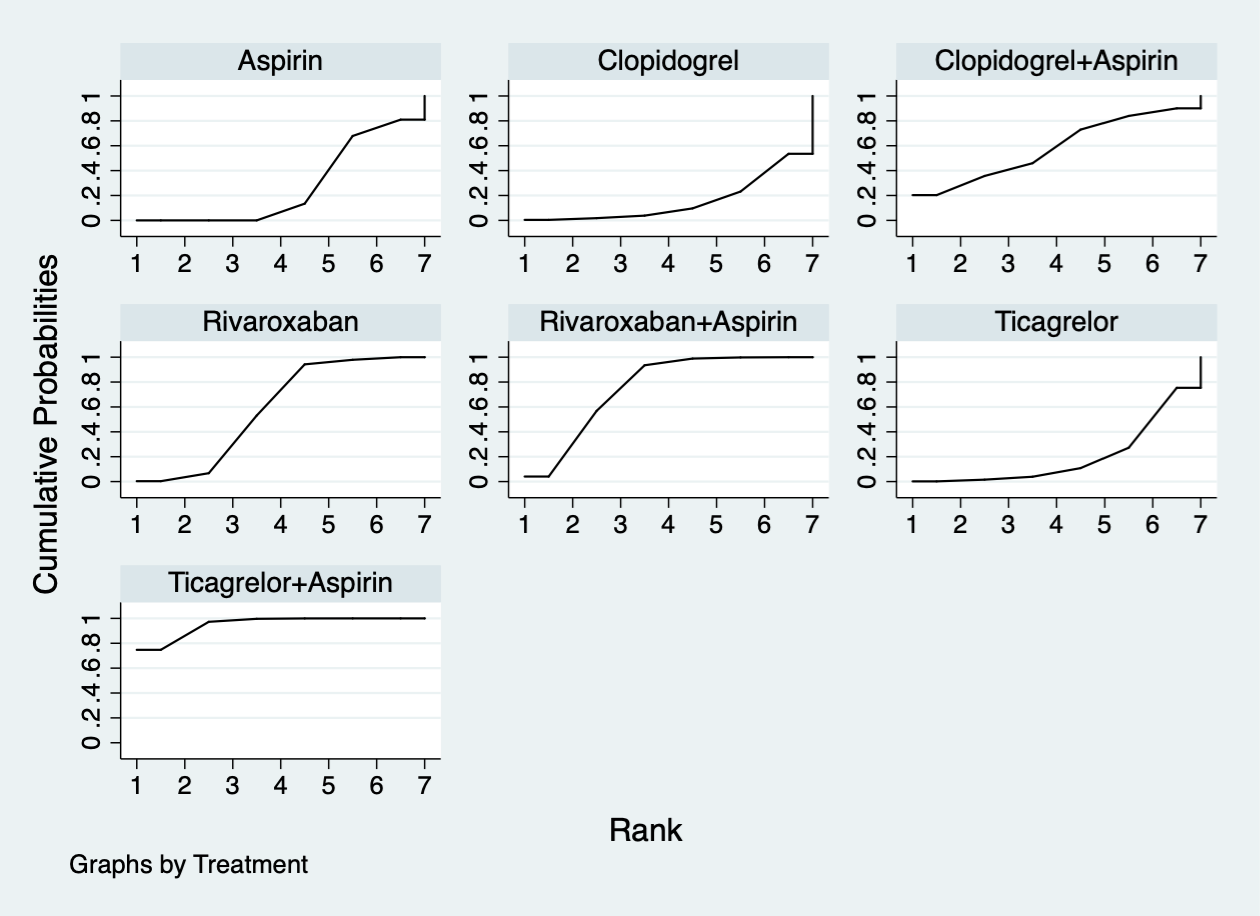


| Drug | Aspirin | Rivaroxaban | Rivaroxaban+Aspirin | Ticagrelor+Aspirin | Clopidogrel+Aspirin | Ticagrelor | Clopidogrel |
| --- | --- | --- | --- | --- | --- | --- | --- |
| Rank 1 | 0 | 0.003 | 0.041 | 0.747 | 0.203 | 0.002 | 0.004 |
| Rank 2 | 0 | 0.064 | 0.528 | 0.226 | 0.154 | 0.014 | 0.014 |
| Rank 3 | 0 | 0.464 | 0.366 | 0.024 | 0.102 | 0.023 | 0.02 |
| Rank 4 | 0.134 | 0.411 | 0.054 | 0.003 | 0.27 | 0.069 | 0.058 |
| Rank 5 | 0.544 | 0.037 | 0.009 | 0 | 0.11 | 0.164 | 0.136 |
| Rank 6 | 0.131 | 0.021 | 0.002 | 0 | 0.061 | 0.481 | 0.304 |
| Rank 7 | 0.19 | 0 | 0 | 0 | 0.099 | 0.246 | 0.464 |
| MeanRank | 5.372 | 3.478 | 2.468 | 1.283 | 3.506 | 5.803 | 6.076 |
| SUCRA | 27.1 | 58.7 | 75.5 | 95.3 | 58.2 | 19.9 | 15.4 |

1. Major adverse cardiovascular and cerebrovascular events for main analysis (without TWILIGHT subgroup) plus the whole cohort of TWILIGHT trial in total cohort


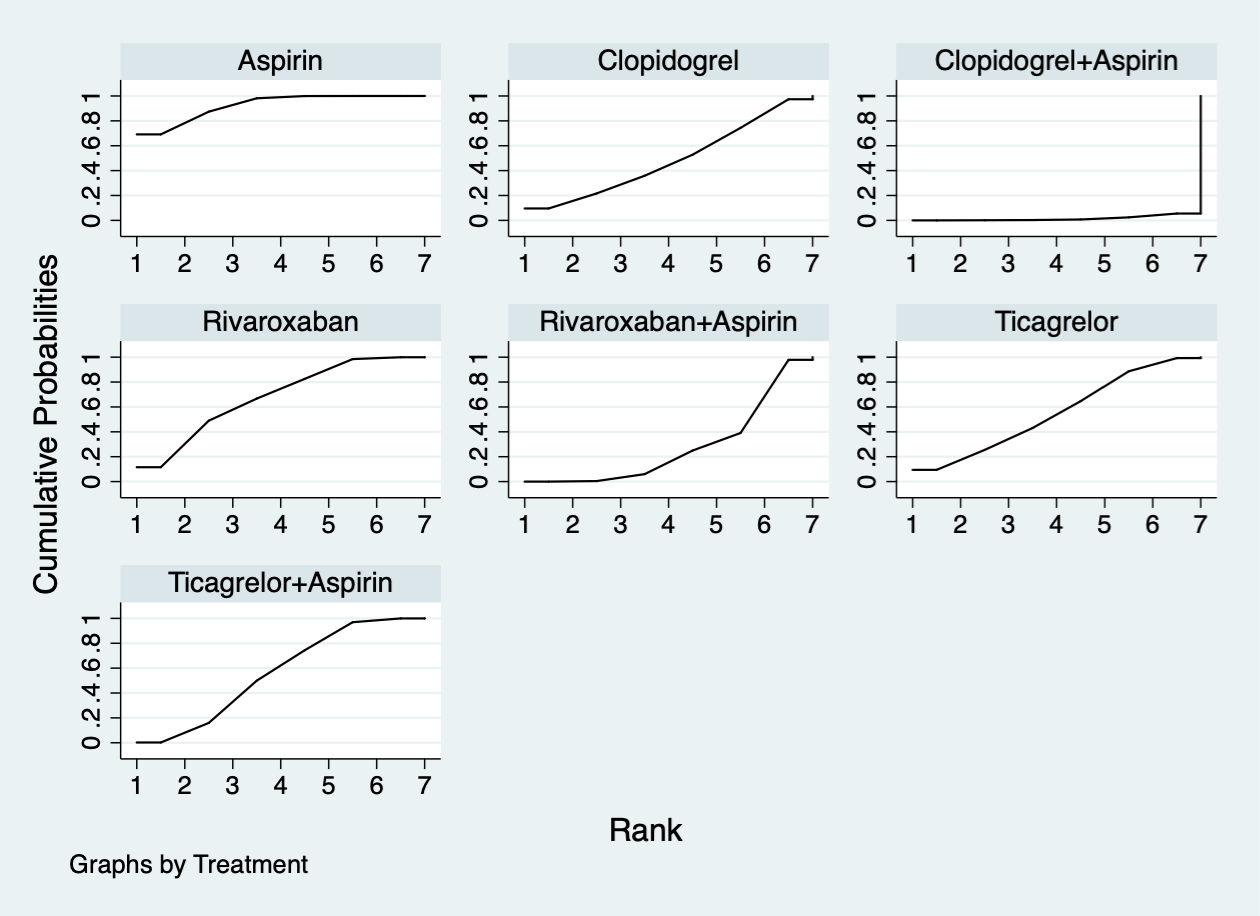


| Drug | Aspirin | Rivaroxaban | Rivaroxaban+Aspirin | Ticagrelor+Aspirin | Clopidogrel+Aspirin | Ticagrelor | Clopidogrel |
| --- | --- | --- | --- | --- | --- | --- | --- |
| Rank 1 | 0.691 | 0.116 | 0 | 0.003 | 0 | 0.095 | 0.96 |
| Rank 2 | 0.183 | 0.374 | 0.004 | 0.157 | 0.001 | 0.16 | 1.21 |
| Rank 3 | 0.107 | 0.177 | 0.056 | 0.339 | 0.002 | 0.176 | 1.42 |
| Rank 4 | 0.018 | 0.159 | 0.19 | 0.245 | 0.004 | 0.215 | 1.69 |
| Rank 5 | 0 | 0.159 | 0.141 | 0.226 | 0.017 | 0.241 | 2.16 |
| Rank 6 | 0 | 0.015 | 0.588 | 0.03 | 0.031 | 0.106 | 2.3 |
| Rank 7 | 0 | 0 | 0.021 | 0 | 0.945 | 0.007 | 0.26 |
| MeanRank | 1.45 | 2.916 | 5.316 | 3.624 | 6.91 | 3.693 | 40.82 |
| SUCRA | 92.4 | 68 | 28.1 | 56.2 | 1.5 | 55.1 | 48.6 |

1. Major bleeding for main analysis (without TWILIGHT subgroup) plus the whole cohort of TWILIGHT trial in total cohort


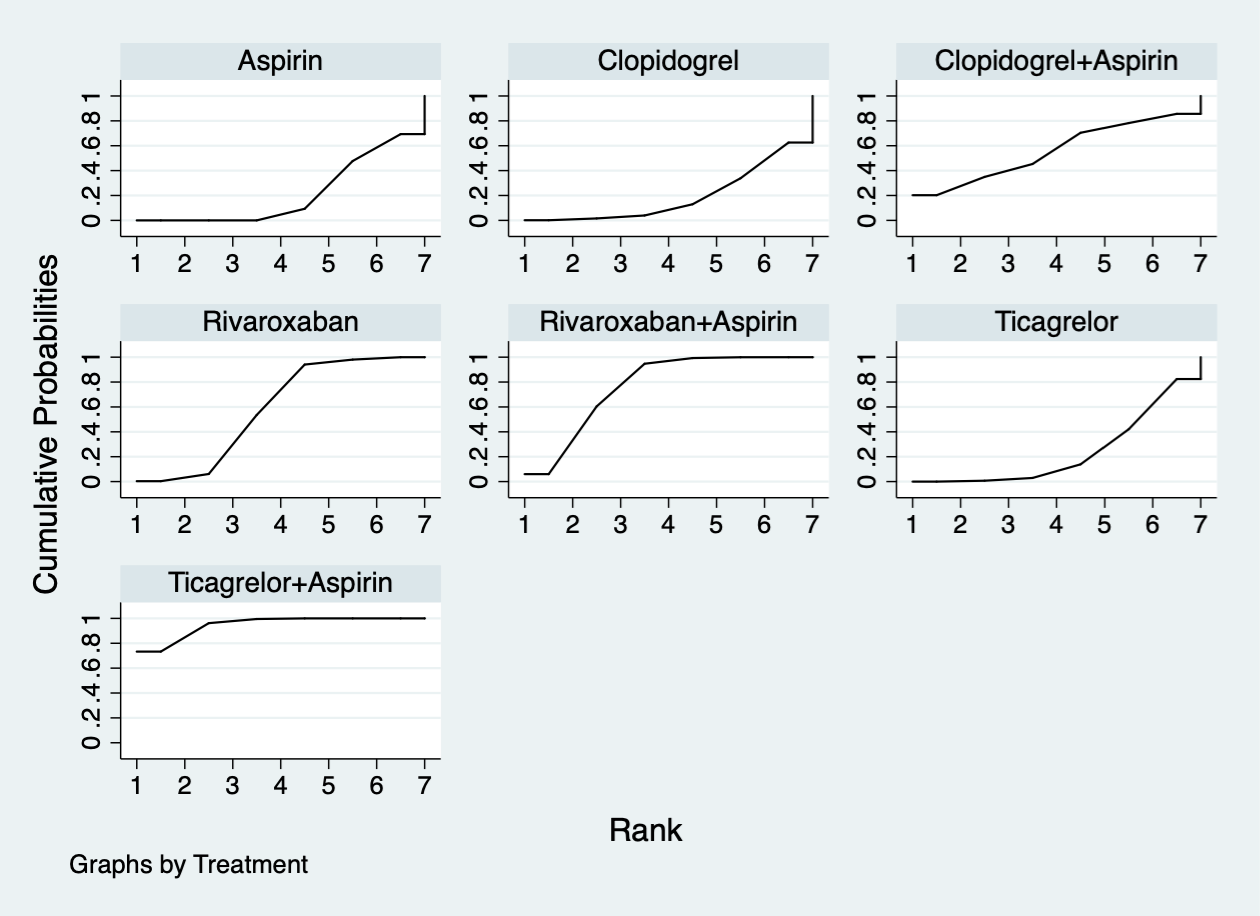


| Drug | Aspirin | Rivaroxaban | Rivaroxaban+Aspirin | Ticagrelor+Aspirin | Clopidogrel+Aspirin | Ticagrelor | Clopidogrel |
| --- | --- | --- | --- | --- | --- | --- | --- |
| Rank 1 | 0 | 0.003 | 0.06 | 0.733 | 0.202 | 0 | 0.001 |
| Rank 2 | 0 | 0.058 | 0.544 | 0.229 | 0.147 | 0.007 | 0.015 |
| Rank 3 | 0 | 0.474 | 0.343 | 0.033 | 0.104 | 0.022 | 0.024 |
| Rank 4 | 0.093 | 0.405 | 0.046 | 0.005 | 0.251 | 0.109 | 0.09 |
| Rank 5 | 0.384 | 0.04 | 0.007 | 0 | 0.078 | 0.282 | 0.209 |
| Rank 6 | 0.216 | 0.019 | 0 | 0 | 0.074 | 0.403 | 0.287 |
| Rank 7 | 0.307 | 0 | 0 | 0 | 0.144 | 0.176 | 0.374 |
| MeanRank | 5.737 | 3.475 | 2.396 | 1.31 | 3.654 | 5.576 | 5.848 |
| SUCRA | 21.1 | 58.7 | 76.7 | 94.8 | 55.8 | 23.7 | 19.2 |

1. Major adverse cardiovascular and cerebrovascular events for main analysis (without DAPT, THEMIS, VOYAGER PAD and TWILIGHT subgroups) plus the whole cohort of DAPT, THEMIS, VOYAGER PAD and TWILIGHT trials in total cohort


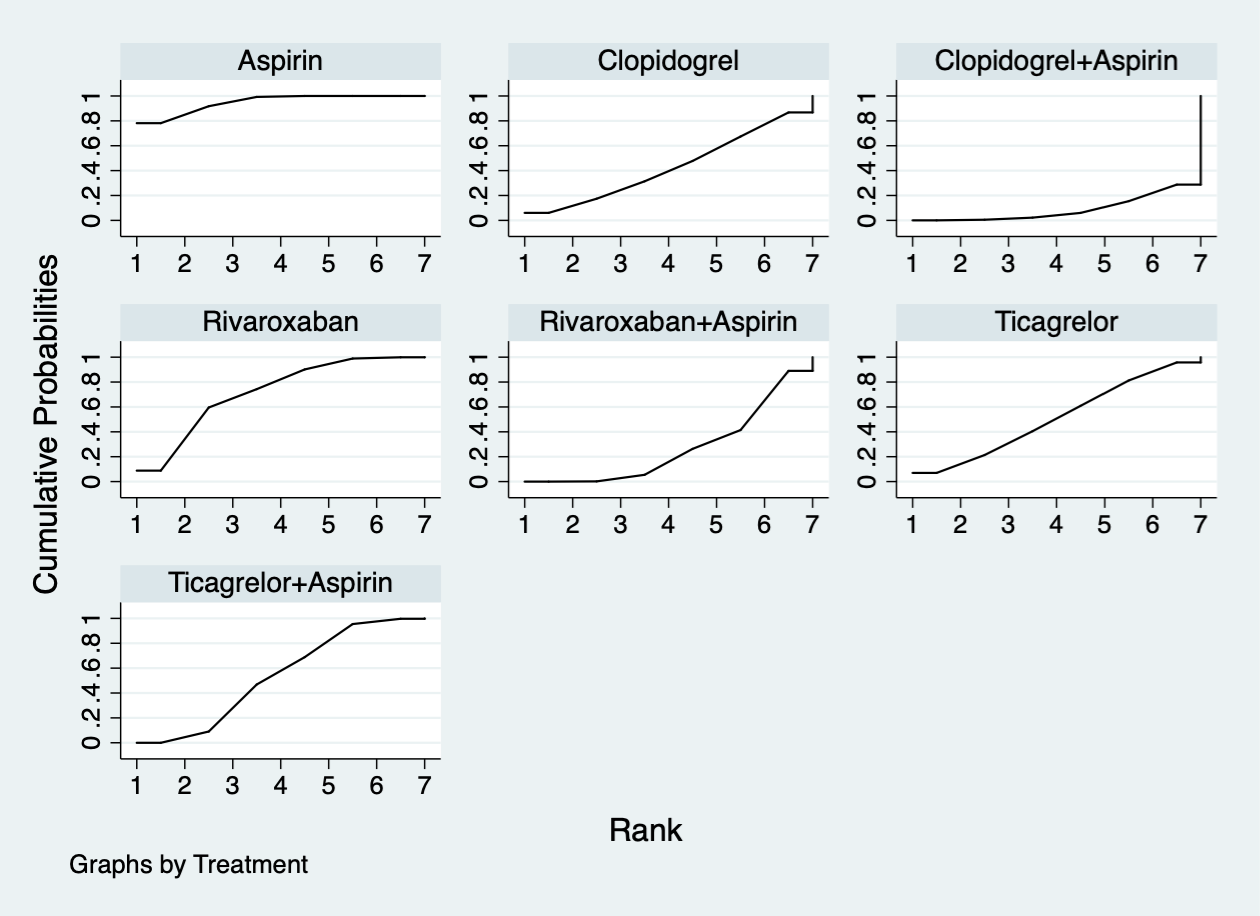


| Drug | Aspirin | Rivaroxaban | Rivaroxaban+Aspirin | Ticagrelor+Aspirin | Clopidogrel+Aspirin | Ticagrelor | Clopidogrel |
| --- | --- | --- | --- | --- | --- | --- | --- |
| Rank 1 | 0.781 | 0.088 | 0 | 0 | 0 | 0.07 | 0.06 |
| Rank 2 | 0.136 | 0.507 | 0.002 | 0.091 | 0.005 | 0.144 | 0.114 |
| Rank 3 | 0.075 | 0.147 | 0.053 | 0.378 | 0.017 | 0.191 | 0.14 |
| Rank 4 | 0.008 | 0.158 | 0.209 | 0.22 | 0.038 | 0.204 | 0.164 |
| Rank 5 | 0 | 0.088 | 0.151 | 0.266 | 0.095 | 0.204 | 0.196 |
| Rank 6 | 0 | 0.01 | 0.476 | 0.042 | 0.133 | 0.145 | 0.194 |
| Rank 7 | 0 | 0.001 | 0.11 | 0.003 | 0.712 | 0.042 | 0.132 |
| MeanRank | 1.31 | 2.682 | 5.38 | 3.799 | 6.47 | 3.931 | 4.432 |
| SUCRA | 94.9 | 71.9 | 27.1 | 53.3 | 8.8 | 51.1 | 42.8 |

1. Major bleeding for main analysis (without DAPT, THEMIS, VOYAGER PAD and TWILIGHT subgroups) plus the whole cohort of DAPT, THEMIS, VOYAGER PAD and TWILIGHT trials in total cohort


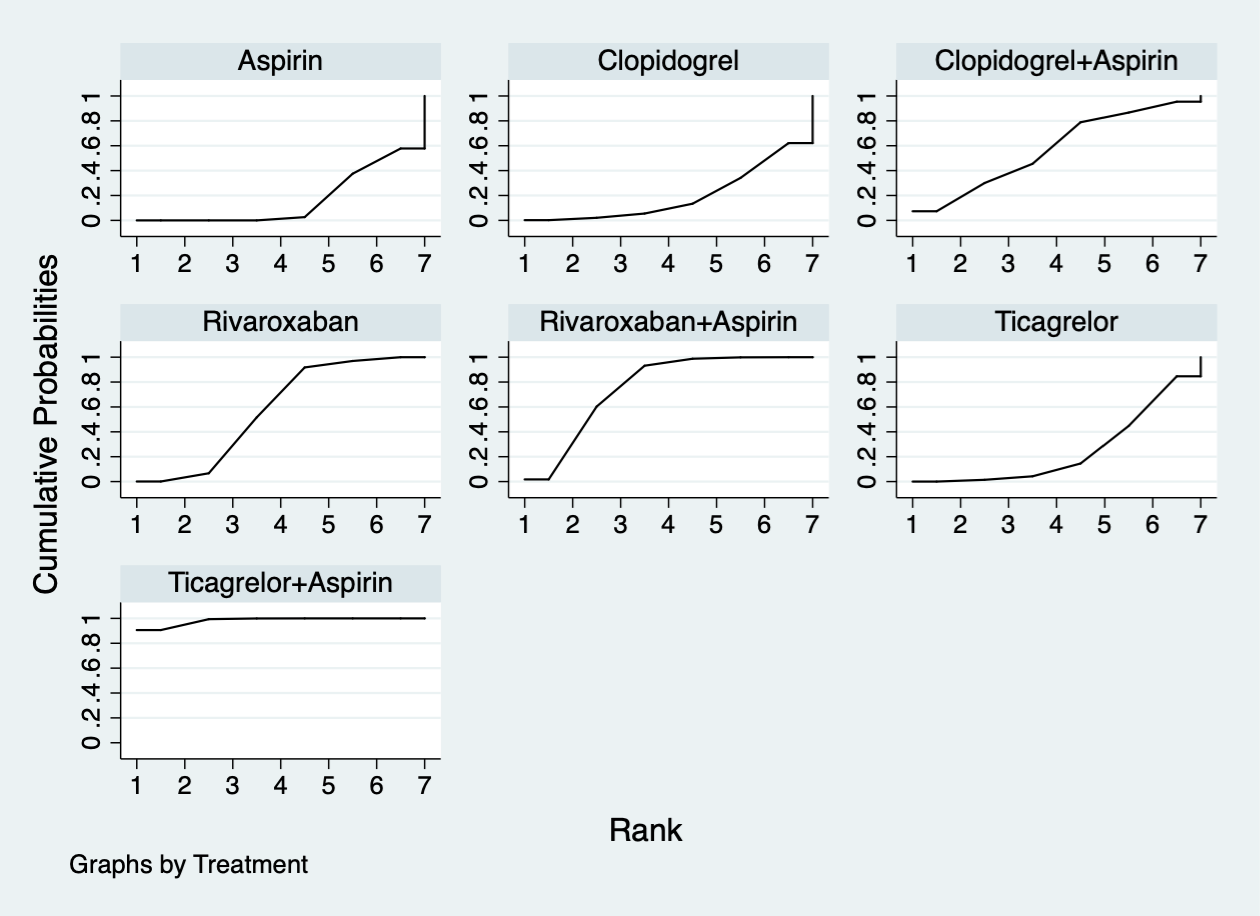


| Drug | Aspirin | Rivaroxaban | Rivaroxaban+Aspirin | Ticagrelor+Aspirin | Clopidogrel+Aspirin | Ticagrelor | Clopidogrel |
| --- | --- | --- | --- | --- | --- | --- | --- |
| Rank 1 | 0 | 0.001 | 0.018 | 0.906 | 0.073 | 0 | 0.002 |
| Rank 2 | 0 | 0.066 | 0.586 | 0.088 | 0.227 | 0.014 | 0.019 |
| Rank 3 | 0 | 0.449 | 0.328 | 0.006 | 0.154 | 0.028 | 0.034 |
| Rank 4 | 0.026 | 0.402 | 0.056 | 0 | 0.334 | 0.103 | 0.079 |
| Rank 5 | 0.349 | 0.052 | 0.011 | 0 | 0.078 | 0.303 | 0.207 |
| Rank 6 | 0.203 | 0.03 | 0.001 | 0 | 0.087 | 0.398 | 0.28 |
| Rank 7 | 0.422 | 0 | 0 | 0 | 0.046 | 0.153 | 0.379 |
| MeanRank | 6.021 | 3.528 | 2.459 | 1.1 | 3.559 | 5.498 | 5.826 |
| SUCRA | 16.3 | 57.9 | 75.6 | 98.3 | 57.3 | 25 | 19.6 |

## eTable 9. Sensitivity analyses for pairwise comparison of efficacy and safety outcomes in the total cohort

Odds ratio (95% credible intervals) between column and row treatment regimens are reported. Odds ratio smaller than 1 means that the odds of having an event for the column treatment regimen is lower than the row treatment regimen. Statistically significant results, where the 95% credible interval does not include 1.

1. Major adverse cardiovascular and cerebrovascular events for main analysis plus CHARISMA trial

| Clopidogrel | 1.14 (0.55,2.36) | 1.37 (0.65,2.88) | 1.18 (0.58,2.41) | 1.02 (0.73,1.43) | 1.31 (0.71,2.44) | 1.49 (0.76,2.89) |
| --- | --- | --- | --- | --- | --- | --- |
| 0.88 (0.42,1.81) | Clopidogrel+Aspirin | 1.20 (0.78,1.87) | 1.04 (0.70,1.52) | 0.90 (0.47,1.70) | 1.15 (0.79,1.68) | 1.30 (0.98,1.74) |
| 0.73 (0.35,1.53) | 0.83 (0.54,1.29) | Rivaroxaban | 0.86 (0.62,1.20) | 0.74 (0.38,1.44) | 0.96 (0.64,1.44) | 1.08 (0.78,1.50) |
| 0.85 (0.42,1.73) | 0.96 (0.66,1.42) | 1.16 (0.84,1.62) | Rivaroxaban+Aspirin | 0.86 (0.46,1.62) | 1.11 (0.78,1.58) | 1.26 (0.97,1.62) |
| 0.98 (0.70,1.38) | 1.12 (0.59,2.12) | 1.35 (0.70,2.60) | 1.16 (0.62,2.17) | Ticagrelor | 1.29 (0.77,2.16) | 1.45 (0.82,2.58) |
| 0.76 (0.41,1.41) | 0.87 (0.59,1.27) | 1.04 (0.70,1.57) | 0.90 (0.63,1.28) | 0.78 (0.46,1.30) | Ticagrelor+Aspirin | 1.13 (0.89,1.44) |
| 0.67 (0.35,1.31) | 0.77 (0.58,1.02) | 0.92 (0.67,1.28) | 0.80 (0.62,1.03) | 0.69 (0.39,1.22) | 0.88 (0.69,1.13) | Aspirin |

1. Major adverse cardiovascular and cerebrovascular events for main analysis plus CAPRIE trial

| Clopidogrel | 0.75 (0.53,1.08) | 1.23 (0.98,1.55) | 1.06 (0.85,1.31) | 1.01 (0.89,1.15) | 1.19 (0.97,1.45) | 1.34 (1.12,1.61) |
| --- | --- | --- | --- | --- | --- | --- |
| 1.33 (0.92,1.90) | Clopidogrel+Aspirin | 1.63 (1.16,2.30) | 1.40 (1.00,1.96) | 1.34 (0.92,1.95) | 1.57 (1.14,2.18) | 1.78 (1.30,2.43) |
| 0.81 (0.64,1.02) | 0.61 (0.43,0.86) | Rivaroxaban | 0.86 (0.74,0.99) | 0.82 (0.64,1.06) | 0.96 (0.81,1.14) | 1.09 (0.94,1.26) |
| 0.95 (0.76,1.18) | 0.71 (0.51,1.00) | 1.17 (1.01,1.35) | Rivaroxaban+Aspirin | 0.96 (0.75,1.22) | 1.12 (0.96,1.31) | 1.27 (1.12,1.43) |
| 0.99 (0.87,1.12) | 0.75 (0.51,1.08) | 1.22 (0.95,1.57) | 1.04 (0.82,1.33) | Ticagrelor | 1.17 (0.94,1.46) | 1.33 (1.08,1.63) |
| 0.84 (0.69,1.03) | 0.64 (0.46,0.88) | 1.04 (0.88,1.23) | 0.89 (0.76,1.04) | 0.85 (0.68,1.06) | Ticagrelor+Aspirin | 1.13 (1.03,1.24) |
| 0.75 (0.62,0.89) | 0.56 (0.41,0.77) | 0.92 (0.80,1.06) | 0.79 (0.70,0.89) | 0.75 (0.61,0.93) | 0.88 (0.80,0.97) | Aspirin |

1. Major adverse cardiovascular and cerebrovascular events for main analysis plus COMMANDER HF trial

| Clopidogrel | 1.08 (0.72,1.60) | 1.32 (0.77,2.27) | 0.91 (0.60,1.38) | 1.44 (0.95,2.20) | 1.28 (0.88,1.86) | 1.03 (0.81,1.29) | 1.34 (0.93,1.92) | 1.52 (1.06,2.17) |
| --- | --- | --- | --- | --- | --- | --- | --- | --- |
| 0.93 (0.63,1.38) | Clopidogrel+Aspirin | 1.23 (0.78,1.93) | 0.84 (0.62,1.14) | 1.34 (0.98,1.84) | 1.19 (0.94,1.52) | 0.95 (0.64,1.43) | 1.24 (0.94,1.64) | 1.41 (1.13,1.76) |
| 0.76 (0.44,1.30) | 0.81 (0.52,1.28) | Clopidogrel+Rivaroxaban | 0.69 (0.43,1.09) | 1.09 (0.67,1.77) | 0.97 (0.63,1.50) | 0.78 (0.45,1.34) | 1.01 (0.64,1.61) | 1.15 (0.74,1.77) |
| 1.10 (0.73,1.67) | 1.19 (0.88,1.60) | 1.46 (0.91,2.33) | Clopidogrel+Rivaroxaban+Aspirin | 1.59 (1.13,2.25) | 1.41 (1.07,1.86) | 1.13 (0.74,1.73) | 1.48 (1.08,2.03) | 1.67 (1.28,2.19) |
| 0.69 (0.45,1.06) | 0.75 (0.54,1.02) | 0.92 (0.56,1.49) | 0.63 (0.44,0.89) | Rivaroxaban | 0.89 (0.70,1.13) | 0.71 (0.46,1.09) | 0.93 (0.69,1.24) | 1.05 (0.83,1.33) |
| 0.78 (0.54,1.13) | 0.84 (0.66,1.07) | 1.03 (0.67,1.60) | 0.71 (0.54,0.93) | 1.13 (0.89,1.43) | Rivaroxaban+Aspirin | 0.80 (0.55,1.17) | 1.04 (0.83,1.31) | 1.18 (1.01,1.38) |
| 0.98 (0.77,1.23) | 1.05 (0.70,1.57) | 1.29 (0.75,2.23) | 0.88 (0.58,1.36) | 1.41 (0.92,2.16) | 1.25 (0.86,1.83) | Ticagrelor | 1.31 (0.91,1.87) | 1.48 (1.03,2.13) |
| 0.75 (0.52,1.07) | 0.80 (0.61,1.06) | 0.99 (0.62,1.57) | 0.68 (0.49,0.93) | 1.08 (0.80,1.45) | 0.96 (0.76,1.20) | 0.77 (0.54,1.09) | Ticagrelor+Aspirin | 1.13 (0.95,1.35) |
| 0.66 (0.46,0.94) | 0.71 (0.57,0.89) | 0.87 (0.56,1.35) | 0.60 (0.46,0.78) | 0.95 (0.75,1.21) | 0.84 (0.72,0.99) | 0.68 (0.47,0.97) | 0.88 (0.74,1.05) | Aspirin |

1. Major adverse cardiovascular and cerebrovascular events for main analysis plus DAVID trial

| Clopidogrel | 0.84 (0.48,1.45) | 1.37 (0.85,2.20) | 1.18 (0.74,1.87) | 1.02 (0.89,1.17) | 1.31 (0.85,2.03) | 1.20 (0.64,2.22) | 1.49 (0.95,2.34) |
| --- | --- | --- | --- | --- | --- | --- | --- |
| 1.19 (0.69,2.06) | Clopidogrel+Aspirin | 1.64 (1.15,2.32) | 1.40 (1.00,1.97) | 1.22 (0.72,2.07) | 1.57 (1.12,2.19) | 1.43 (0.84,2.43) | 1.78 (1.30,2.44) |
| 0.73 (0.45,1.17) | 0.61 (0.43,0.87) | Rivaroxaban | 0.86 (0.74,1.00) | 0.74 (0.47,1.17) | 0.96 (0.80,1.15) | 0.87 (0.55,1.37) | 1.09 (0.94,1.26) |
| 0.85 (0.53,1.35) | 0.71 (0.51,1.00) | 1.17 (1.00,1.36) | Rivaroxaban+Aspirin | 0.87 (0.56,1.35) | 1.12 (0.95,1.31) | 1.02 (0.65,1.59) | 1.27 (1.12,1.44) |
| 0.98 (0.85,1.12) | 0.82 (0.48,1.40) | 1.34 (0.85,2.11) | 1.15 (0.74,1.80) | Ticagrelor | 1.29 (0.85,1.95) | 1.17 (0.64,2.14) | 1.46 (0.95,2.24) |
| 0.76 (0.49,1.18) | 0.64 (0.46,0.89) | 1.04 (0.87,1.25) | 0.89 (0.76,1.05) | 0.78 (0.51,1.18) | Ticagrelor+Aspirin | 0.91 (0.59,1.41) | 1.14 (1.03,1.26) |
| 0.84 (0.45,1.55) | 0.70 (0.41,1.19) | 1.15 (0.73,1.80) | 0.98 (0.63,1.54) | 0.85 (0.47,1.56) | 1.10 (0.71,1.71) | Picotamide | 1.25 (0.81,1.91) |
| 0.67 (0.43,1.05) | 0.56 (0.41,0.77) | 0.92 (0.79,1.07) | 0.79 (0.69,0.89) | 0.68 (0.45,1.05) | 0.88 (0.80,0.98) | 0.80 (0.52,1.23) | Aspirin |

1. Major adverse cardiovascular and cerebrovascular events for main analysis plus CHARISMA, CAPRIE, COMMANDER HF, and DAVID trials

| Clopidogrel | 1.10 (0.83,1.46) | 1.26 (0.77,2.04) | 0.86 (0.61,1.22) | 1.33 (0.95,1.86) | 1.19 (0.91,1.57) | 1.01 (0.79,1.28) | 1.23 (0.94,1.62) | 1.11 (0.65,1.89) | 1.38 (1.09,1.74) |
| --- | --- | --- | --- | --- | --- | --- | --- | --- | --- |
| 0.91 (0.69,1.21) | Clopidogrel+Aspirin | 1.14 (0.73,1.80) | 0.78 (0.58,1.05) | 1.21 (0.90,1.64) | 1.09 (0.87,1.35) | 0.92 (0.65,1.28) | 1.12 (0.87,1.45) | 1.01 (0.60,1.69) | 1.26 (1.05,1.51) |
| 0.80 (0.49,1.29) | 0.87 (0.56,1.37) | Clopidogrel+Rivaroxaban | 0.69 (0.43,1.10) | 1.06 (0.64,1.73) | 0.95 (0.61,1.48) | 0.80 (0.48,1.35) | 0.98 (0.61,1.57) | 0.88 (0.46,1.69) | 1.10 (0.71,1.70) |
| 1.16 (0.82,1.64) | 1.27 (0.95,1.71) | 1.46 (0.91,2.35) | Clopidogrel+Rivaroxaban+Aspirin | 1.54 (1.08,2.21) | 1.38 (1.04,1.84) | 1.17 (0.79,1.73) | 1.43 (1.03,1.98) | 1.28 (0.74,2.24) | 1.60 (1.22,2.11) |
| 0.75 (0.54,1.06) | 0.83 (0.61,1.12) | 0.95 (0.58,1.55) | 0.65 (0.45,0.93) | Rivaroxaban | 0.90 (0.70,1.16) | 0.76 (0.51,1.12) | 0.93 (0.68,1.26) | 0.83 (0.48,1.44) | 1.04 (0.81,1.34) |
| 0.84 (0.64,1.10) | 0.92 (0.74,1.15) | 1.05 (0.68,1.64) | 0.72 (0.54,0.96) | 1.11 (0.87,1.43) | Rivaroxaban+Aspirin | 0.84 (0.60,1.18) | 1.03 (0.81,1.31) | 0.93 (0.56,1.54) | 1.16 (0.99,1.36) |
| 0.99 (0.78,1.26) | 1.09 (0.78,1.53) | 1.25 (0.74,2.11) | 0.86 (0.58,1.27) | 1.32 (0.90,1.95) | 1.19 (0.85,1.65) | Ticagrelor | 1.22 (0.90,1.67) | 1.10 (0.62,1.94) | 1.37 (1.02,1.85) |
| 0.81 (0.62,1.07) | 0.89 (0.69,1.15) | 1.02 (0.64,1.64) | 0.70 (0.50,0.97) | 1.08 (0.79,1.47) | 0.97 (0.76,1.23) | 0.82 (0.60,1.12) | Ticagrelor+Aspirin | 0.90 (0.54,1.50) | 1.12 (0.94,1.34) |
| 0.90 (0.53,1.55) | 0.99 (0.59,1.66) | 1.14 (0.59,2.18) | 0.78 (0.45,1.36) | 1.20 (0.70,2.07) | 1.08 (0.65,1.79) | 0.91 (0.51,1.60) | 1.11 (0.66,1.86) | Picotamide | 1.25 (0.77,2.02) |
| 0.72 (0.57,0.91) | 0.79 (0.66,0.95) | 0.91 (0.59,1.41) | 0.62 (0.47,0.82) | 0.96 (0.75,1.24) | 0.86 (0.73,1.01) | 0.73 (0.54,0.98) | 0.89 (0.74,1.07) | 0.80 (0.49,1.30) | Aspirin |

1. Major adverse cardiovascular and cerebrovascular events for network meta-analysis structure combining ticagrelor and clopidogrel into P2Y12 inhibitor

| P2Y12-inhibitor | 1.29 (0.77,2.15) | 1.49 (0.78,2.83) | 1.28 (0.70,2.36) | 1.61 (0.92,2.81) |
| --- | --- | --- | --- | --- |
| 0.78 (0.47,1.30) | P2Y12-inhibitor+Aspirin | 1.16 (0.79,1.71) | 1.00 (0.71,1.39) | 1.25 (1.00,1.56) |
| 0.67 (0.35,1.28) | 0.86 (0.59,1.27) | Rivaroxaban | 0.86 (0.63,1.18) | 1.08 (0.79,1.49) |
| 0.78 (0.42,1.44) | 1.00 (0.72,1.40) | 1.16 (0.84,1.60) | Rivaroxaban+Aspirin | 1.26 (0.98,1.61) |
| 0.62 (0.36,1.08) | 0.80 (0.64,1.00) | 0.92 (0.67,1.27) | 0.80 (0.62,1.02) | Aspirin |

1. Major bleeding for main analysis plus CHARISMA trial

| Clopidogrel | 1.60 (0.72,3.54) | 1.90 (0.87,4.13) | 2.13 (0.98,4.63) | 1.04 (0.80,1.36) | 2.58 (1.25,5.33) | 1.26 (0.59,2.68) |
| --- | --- | --- | --- | --- | --- | --- |
| 0.63 (0.28,1.38) | Clopidogrel+Aspirin | 1.19 (0.87,1.62) | 1.33 (0.98,1.81) | 0.65 (0.31,1.38) | 1.62 (1.17,2.24) | 0.79 (0.62,1.01) |
| 0.53 (0.24,1.15) | 0.84 (0.62,1.15) | Rivaroxaban | 1.12 (0.95,1.33) | 0.55 (0.26,1.14) | 1.36 (1.03,1.81) | 0.67 (0.55,0.81) |
| 0.47 (0.22,1.02) | 0.75 (0.55,1.02) | 0.89 (0.75,1.05) | Rivaroxaban+Aspirin | 0.49 (0.24,1.01) | 1.21 (0.92,1.60) | 0.59 (0.49,0.71) |
| 0.96 (0.74,1.25) | 1.54 (0.73,3.25) | 1.82 (0.88,3.79) | 2.05 (0.99,4.25) | Ticagrelor | 2.48 (1.26,4.87) | 1.21 (0.60,2.46) |
| 0.39 (0.19,0.80) | 0.62 (0.45,0.86) | 0.73 (0.55,0.97) | 0.82 (0.63,1.09) | 0.40 (0.21,0.79) | Ticagrelor+Aspirin | 0.49 (0.40,0.60) |
| 0.79 (0.37,1.69) | 1.27 (0.99,1.62) | 1.50 (1.24,1.82) | 1.69 (1.41,2.03) | 0.83 (0.41,1.67) | 2.05 (1.66,2.52) | Aspirin |

1. Major bleeding for network meta-analysis structure combining ticagrelor and clopidogrel into P2Y12 inhibitor

| P2Y12-inhibitor | 2.48 (1.26,4.87) | 1.86 (0.90,3.86) | 2.09 (1.01,4.33) | 1.24 (0.61,2.50) |
| --- | --- | --- | --- | --- |
| 0.40 (0.21,0.79) | P2Y12-inhibitor+Aspirin | 0.75 (0.57,0.99) | 0.84 (0.64,1.11) | 0.50 (0.41,0.61) |
| 0.54 (0.26,1.12) | 1.33 (1.01,1.76) | Rivaroxaban | 1.12 (0.95,1.33) | 0.67 (0.55,0.81) |
| 0.48 (0.23,0.99) | 1.19 (0.90,1.56) | 0.89 (0.75,1.05) | Rivaroxaban+Aspirin | 0.59 (0.49,0.71) |
| 0.81 (0.40,1.63) | 2.01 (1.64,2.46) | 1.50 (1.24,1.82) | 1.69 (1.41,2.03) | Aspirin |

1. TIMI major bleeding for network meta-analysis structure

| Ticagrelor+Aspirin | 0.74 (0.40,1.35) | 0.49 (0.40,0.60) |
| --- | --- | --- |
| 1.36 (0.74,2.49) | Rivaroxaban+Aspirin | 0.66 (0.38,1.17) |
| 2.05 (1.66,2.52) | 1.51 (0.85,2.67) | Aspirin |

1. BARC (2, 3, or 5) bleeding for network meta-analysis structure

| P2Y12-inhibitor+Aspirin | 0.53 (0.22,1.24) | 0.46 (0.06,3.53) |
| --- | --- | --- |
| 1.90 (0.81,4.48) | P2Y12-inhibitor | 0.87 (0.05,14.70) |
| 2.18 (0.28,16.78) | 1.15 (0.07,19.31) | Aspirin |

1. Major adverse cardiovascular and cerebrovascular events for main analysis (without DAPT subgroup) plus the whole cohort of DAPT trial

| Clopidogrel | 1.07 (0.66,1.72) | 1.37 (0.86,2.18) | 1.18 (0.74,1.86) | 1.02 (0.90,1.16) | 1.31 (0.85,2.02) | 1.49 (0.96,2.32) |
| --- | --- | --- | --- | --- | --- | --- |
| 0.94 (0.58,1.51) | Clopidogrel+Aspirin | 1.28 (1.02,1.61) | 1.10 (0.89,1.36) | 0.95 (0.60,1.51) | 1.23 (1.00,1.50) | 1.40 (1.17,1.67) |
| 0.73 (0.46,1.16) | 0.78 (0.62,0.98) | Rivaroxaban | 0.86 (0.74,0.99) | 0.74 (0.48,1.16) | 0.96 (0.81,1.14) | 1.09 (0.94,1.26) |
| 0.85 (0.54,1.34) | 0.91 (0.73,1.13) | 1.17 (1.01,1.35) | Rivaroxaban+Aspirin | 0.87 (0.56,1.35) | 1.12 (0.96,1.30) | 1.27 (1.12,1.43) |
| 0.98 (0.86,1.11) | 1.05 (0.66,1.66) | 1.34 (0.86,2.10) | 1.15 (0.74,1.79) | Ticagrelor | 1.29 (0.85,1.94) | 1.46 (0.96,2.23) |
| 0.76 (0.49,1.17) | 0.81 (0.66,1.00) | 1.04 (0.88,1.24) | 0.89 (0.77,1.04) | 0.78 (0.51,1.17) | Ticagrelor+Aspirin | 1.14 (1.03,1.25) |
| 0.67 (0.43,1.04) | 0.72 (0.60,0.86) | 0.92 (0.80,1.06) | 0.79 (0.70,0.89) | 0.68 (0.45,1.04) | 0.88 (0.80,0.97) | Aspirin |

1. Major bleeding for main analysis (without DAPT subgroup) plus the whole cohort of DAPT trial

| Clopidogrel | 1.82 (0.74,4.51) | 1.90 (0.87,4.13) | 2.13 (0.98,4.63) | 1.04 (0.80,1.36) | 2.58 (1.25,5.33) | 1.26 (0.59,2.68) |
| --- | --- | --- | --- | --- | --- | --- |
| 0.55 (0.22,1.36) | Clopidogrel+Aspirin | 1.04 (0.61,1.78) | 1.17 (0.69,1.99) | 0.57 (0.24,1.36) | 1.42 (0.82,2.44) | 0.69 (0.42,1.14) |
| 0.53 (0.24,1.15) | 0.96 (0.56,1.64) | Rivaroxaban | 1.12 (0.95,1.33) | 0.55 (0.26,1.14) | 1.36 (1.03,1.81) | 0.67 (0.55,0.81) |
| 0.47 (0.22,1.02) | 0.86 (0.50,1.46) | 0.89 (0.75,1.05) | Rivaroxaban+Aspirin | 0.49 (0.24,1.01) | 1.21 (0.92,1.60) | 0.59 (0.49,0.71) |
| 0.96 (0.74,1.25) | 1.75 (0.74,4.16) | 1.82 (0.88,3.79) | 2.05 (0.99,4.25) | Ticagrelor | 2.48 (1.26,4.87) | 1.21 (0.60,2.46) |
| 0.39 (0.19,0.80) | 0.71 (0.41,1.21) | 0.73 (0.55,0.97) | 0.82 (0.63,1.09) | 0.40 (0.21,0.79) | Ticagrelor+Aspirin | 0.49 (0.40,0.60) |
| 0.79 (0.37,1.69) | 1.45 (0.88,2.39) | 1.50 (1.24,1.82) | 1.69 (1.41,2.03) | 0.83 (0.41,1.67) | 2.05 (1.66,2.52) | Aspirin |

1. Major adverse cardiovascular and cerebrovascular events for main analysis (without THEMIS subgroup) plus the whole cohort of THEMIS trial

| Clopidogrel | 0.85 (0.51,1.44) | 1.39 (0.89,2.17) | 1.19 (0.77,1.85) | 1.02 (0.92,1.14) | 1.31 (0.86,2.00) | 1.52 (0.99,2.33) |
| --- | --- | --- | --- | --- | --- | --- |
| 1.17 (0.69,1.98) | Clopidogrel+Aspirin | 1.63 (1.17,2.27) | 1.40 (1.01,1.93) | 1.20 (0.72,2.00) | 1.54 (1.12,2.11) | 1.78 (1.31,2.41) |
| 0.72 (0.46,1.12) | 0.61 (0.44,0.85) | Rivaroxaban | 0.86 (0.75,0.98) | 0.73 (0.48,1.13) | 0.94 (0.81,1.09) | 1.09 (0.96,1.24) |
| 0.84 (0.54,1.30) | 0.72 (0.52,0.99) | 1.17 (1.02,1.33) | Rivaroxaban+Aspirin | 0.86 (0.56,1.31) | 1.10 (0.96,1.26) | 1.27 (1.14,1.42) |
| 0.98 (0.88,1.09) | 0.84 (0.50,1.40) | 1.36 (0.89,2.10) | 1.17 (0.76,1.79) | Ticagrelor | 1.29 (0.86,1.93) | 1.49 (0.99,2.25) |
| 0.76 (0.50,1.16) | 0.65 (0.47,0.89) | 1.06 (0.92,1.23) | 0.91 (0.80,1.04) | 0.78 (0.52,1.17) | Ticagrelor+Aspirin | 1.16 (1.07,1.25) |
| 0.66 (0.43,1.01) | 0.56 (0.41,0.76) | 0.92 (0.81,1.04) | 0.79 (0.70,0.88) | 0.67 (0.44,1.01) | 0.86 (0.80,0.93) | Aspirin |

1. Major bleeding for main analysis (without THEMIS subgroup) plus the whole cohort of THEMIS trial

| Clopidogrel | 1.71 (0.55,5.30) | 1.81 (0.83,3.91) | 2.03 (0.94,4.38) | 1.04 (0.80,1.36) | 2.58 (1.25,5.33) | 1.20 (0.57,2.54) |
| --- | --- | --- | --- | --- | --- | --- |
| 0.59 (0.19,1.82) | Clopidogrel+Aspirin | 1.06 (0.44,2.54) | 1.19 (0.50,2.84) | 0.61 (0.20,1.84) | 1.51 (0.63,3.63) | 0.70 (0.30,1.65) |
| 0.55 (0.26,1.20) | 0.94 (0.39,2.26) | Rivaroxaban | 1.12 (0.95,1.33) | 0.58 (0.28,1.19) | 1.43 (1.09,1.87) | 0.67 (0.55,0.81) |
| 0.49 (0.23,1.06) | 0.84 (0.35,2.01) | 0.89 (0.75,1.05) | Rivaroxaban+Aspirin | 0.51 (0.25,1.06) | 1.27 (0.98,1.65) | 0.59 (0.49,0.71) |
| 0.96 (0.74,1.25) | 1.64 (0.54,4.94) | 1.74 (0.84,3.59) | 1.95 (0.95,4.02) | Ticagrelor | 2.48 (1.26,4.87) | 1.15 (0.57,2.32) |
| 0.39 (0.19,0.80) | 0.66 (0.28,1.58) | 0.70 (0.53,0.91) | 0.79 (0.61,1.02) | 0.40 (0.21,0.79) | Ticagrelor+Aspirin | 0.46 (0.39,0.56) |
| 0.83 (0.39,1.76) | 1.42 (0.61,3.33) | 1.50 (1.24,1.82) | 1.69 (1.41,2.03) | 0.87 (0.43,1.74) | 2.15 (1.78,2.59) | Aspirin |

1. Major adverse cardiovascular and cerebrovascular events for main analysis (without VOYAGER PAD subgroup) plus the whole cohort of VOYAGER PAD trial

| Clopidogrel | 0.84 (0.48,1.46) | 1.38 (0.86,2.22) | 1.19 (0.75,1.90) | 1.02 (0.89,1.18) | 1.31 (0.85,2.04) | 1.49 (0.95,2.34) |
| --- | --- | --- | --- | --- | --- | --- |
| 1.19 (0.69,2.08) | Clopidogrel+Aspirin | 1.64 (1.16,2.34) | 1.42 (1.01,1.99) | 1.22 (0.71,2.08) | 1.57 (1.12,2.19) | 1.78 (1.29,2.45) |
| 0.73 (0.45,1.17) | 0.61 (0.43,0.87) | Rivaroxaban | 0.86 (0.74,1.01) | 0.74 (0.47,1.17) | 0.95 (0.79,1.15) | 1.08 (0.93,1.26) |
| 0.84 (0.53,1.34) | 0.70 (0.50,0.99) | 1.16 (0.99,1.35) | Rivaroxaban+Aspirin | 0.86 (0.55,1.34) | 1.10 (0.94,1.29) | 1.25 (1.12,1.41) |
| 0.98 (0.85,1.13) | 0.82 (0.48,1.40) | 1.35 (0.86,2.13) | 1.17 (0.75,1.82) | Ticagrelor | 1.29 (0.85,1.95) | 1.46 (0.95,2.24) |
| 0.76 (0.49,1.18) | 0.64 (0.46,0.89) | 1.05 (0.87,1.26) | 0.91 (0.77,1.06) | 0.78 (0.51,1.18) | Ticagrelor+Aspirin | 1.13 (1.02,1.26) |
| 0.67 (0.43,1.05) | 0.56 (0.41,0.77) | 0.92 (0.79,1.07) | 0.80 (0.71,0.89) | 0.68 (0.45,1.05) | 0.88 (0.79,0.98) | Aspirin |

1. Major bleeding for main analysis (without VOYAGER PAD subgroup) plus the whole cohort of VOYAGER PAD trial

| Clopidogrel | 1.79 (0.57,5.59) | 1.87 (0.86,4.06) | 2.08 (0.96,4.51) | 1.04 (0.80,1.36) | 2.58 (1.25,5.33) | 1.26 (0.59,2.68) |
| --- | --- | --- | --- | --- | --- | --- |
| 0.56 (0.18,1.74) | Clopidogrel+Aspirin | 1.04 (0.44,2.50) | 1.16 (0.49,2.77) | 0.58 (0.19,1.76) | 1.44 (0.60,3.47) | 0.70 (0.30,1.65) |
| 0.54 (0.25,1.17) | 0.96 (0.40,2.30) | Rivaroxaban | 1.11 (0.94,1.32) | 0.56 (0.27,1.16) | 1.38 (1.04,1.83) | 0.68 (0.56,0.82) |
| 0.48 (0.22,1.04) | 0.86 (0.36,2.06) | 0.90 (0.76,1.06) | Rivaroxaban+Aspirin | 0.50 (0.24,1.03) | 1.24 (0.95,1.63) | 0.61 (0.51,0.72) |
| 0.96 (0.74,1.25) | 1.72 (0.57,5.21) | 1.79 (0.86,3.73) | 2.00 (0.97,4.13) | Ticagrelor | 2.48 (1.26,4.87) | 1.21 (0.60,2.46) |
| 0.39 (0.19,0.80) | 0.69 (0.29,1.67) | 0.72 (0.55,0.96) | 0.80 (0.61,1.05) | 0.40 (0.21,0.79) | Ticagrelor+Aspirin | 0.49 (0.40,0.60) |
| 0.79 (0.37,1.69) | 1.42 (0.61,3.33) | 1.48 (1.23,1.79) | 1.65 (1.39,1.96) | 0.83 (0.41,1.67) | 2.05 (1.66,2.52) | Aspirin |

1. Major adverse cardiovascular and cerebrovascular events for main analysis (without TWILIGHT subgroup) plus the whole cohort of TWILIGHT trial

| Clopidogrel | 0.66 (0.42,1.03) | 1.08 (0.77,1.52) | 0.93 (0.66,1.29) | 1.02 (0.89,1.17) | 1.03 (0.77,1.38) | 1.17 (0.86,1.60) |
| --- | --- | --- | --- | --- | --- | --- |
| 1.52 (0.98,2.36) | Clopidogrel+Aspirin | 1.64 (1.15,2.32) | 1.40 (1.00,1.97) | 1.55 (1.02,2.36) | 1.57 (1.12,2.19) | 1.78 (1.30,2.44) |
| 0.93 (0.66,1.31) | 0.61 (0.43,0.87) | Rivaroxaban | 0.86 (0.74,1.00) | 0.95 (0.69,1.30) | 0.96 (0.80,1.15) | 1.09 (0.94,1.26) |
| 1.08 (0.78,1.51) | 0.71 (0.51,1.00) | 1.17 (1.00,1.36) | Rivaroxaban+Aspirin | 1.10 (0.82,1.49) | 1.12 (0.95,1.31) | 1.27 (1.12,1.44) |
| 0.98 (0.85,1.12) | 0.65 (0.42,0.98) | 1.06 (0.77,1.45) | 0.91 (0.67,1.23) | Ticagrelor | 1.01 (0.78,1.31) | 1.15 (0.87,1.52) |
| 0.97 (0.72,1.29) | 0.64 (0.46,0.89) | 1.04 (0.87,1.25) | 0.89 (0.76,1.05) | 0.99 (0.76,1.28) | Ticagrelor+Aspirin | 1.14 (1.03,1.26) |
| 0.85 (0.63,1.16) | 0.56 (0.41,0.77) | 0.92 (0.79,1.07) | 0.79 (0.69,0.89) | 0.87 (0.66,1.15) | 0.88 (0.80,0.98) | Aspirin |

1. Major bleeding for main analysis (without TWILIGHT subgroup) plus the whole cohort of TWILIGHT trial

| Clopidogrel | 1.48 (0.54,4.03) | 1.56 (0.89,2.75) | 1.75 (1.00,3.08) | 1.04 (0.80,1.36) | 2.13 (1.30,3.48) | 1.04 (0.61,1.77) |
| --- | --- | --- | --- | --- | --- | --- |
| 0.68 (0.25,1.85) | Clopidogrel+Aspirin | 1.06 (0.44,2.54) | 1.19 (0.50,2.84) | 0.71 (0.27,1.86) | 1.44 (0.60,3.47) | 0.70 (0.30,1.65) |
| 0.64 (0.36,1.13) | 0.94 (0.39,2.26) | Rivaroxaban | 1.12 (0.95,1.33) | 0.67 (0.40,1.10) | 1.36 (1.03,1.81) | 0.67 (0.55,0.81) |
| 0.57 (0.32,1.00) | 0.84 (0.35,2.01) | 0.89 (0.75,1.05) | Rivaroxaban+Aspirin | 0.59 (0.36,0.98) | 1.21 (0.92,1.60) | 0.59 (0.49,0.71) |
| 0.96 (0.74,1.25) | 1.42 (0.54,3.74) | 1.50 (0.91,2.48) | 1.69 (1.03,2.77) | Ticagrelor | 2.04 (1.35,3.09) | 1.00 (0.63,1.59) |
| 0.47 (0.29,0.77) | 0.69 (0.29,1.67) | 0.73 (0.55,0.97) | 0.82 (0.63,1.09) | 0.49 (0.32,0.74) | Ticagrelor+Aspirin | 0.49 (0.40,0.60) |
| 0.96 (0.56,1.64) | 1.42 (0.61,3.33) | 1.50 (1.24,1.82) | 1.69 (1.41,2.03) | 1.00 (0.63,1.59) | 2.05 (1.66,2.52) | Aspirin |

1. Major adverse cardiovascular and cerebrovascular events for main analysis (without DAPT, THEMIS, VOYAGER PAD and TWILIGHT subgroups) plus the whole cohort of DAPT, THEMIS, VOYAGER PAD and TWILIGHT trials

| Clopidogrel | 0.86 (0.62,1.19) | 1.10 (0.82,1.49) | 0.95 (0.71,1.28) | 1.02 (0.92,1.14) | 1.03 (0.79,1.35) | 1.20 (0.91,1.57) |
| --- | --- | --- | --- | --- | --- | --- |
| 1.17 (0.84,1.62) | Clopidogrel+Aspirin | 1.29 (1.03,1.61) | 1.11 (0.91,1.37) | 1.19 (0.87,1.63) | 1.21 (0.99,1.47) | 1.40 (1.16,1.67) |
| 0.91 (0.67,1.23) | 0.78 (0.62,0.97) | Rivaroxaban | 0.86 (0.76,0.98) | 0.92 (0.70,1.23) | 0.94 (0.81,1.08) | 1.08 (0.96,1.23) |
| 1.05 (0.78,1.40) | 0.90 (0.73,1.10) | 1.16 (1.02,1.31) | Rivaroxaban+Aspirin | 1.07 (0.82,1.40) | 1.08 (0.96,1.22) | 1.25 (1.14,1.38) |
| 0.98 (0.88,1.09) | 0.84 (0.61,1.15) | 1.08 (0.82,1.43) | 0.94 (0.71,1.23) | Ticagrelor | 1.01 (0.79,1.29) | 1.17 (0.91,1.51) |
| 0.97 (0.74,1.26) | 0.83 (0.68,1.01) | 1.07 (0.92,1.23) | 0.92 (0.82,1.04) | 0.99 (0.77,1.26) | Ticagrelor+Aspirin | 1.16 (1.07,1.25) |
| 0.84 (0.64,1.10) | 0.72 (0.60,0.86) | 0.92 (0.82,1.04) | 0.80 (0.73,0.88) | 0.85 (0.66,1.10) | 0.86 (0.80,0.93) | Aspirin |

1. Major bleeding for main analysis (without DAPT, THEMIS, VOYAGER PAD and TWILIGHT subgroups) plus the whole cohort of DAPT, THEMIS, VOYAGER PAD and TWILIGHT trials

| Clopidogrel | 1.43 (0.69,2.96) | 1.46 (0.84,2.56) | 1.63 (0.94,2.83) | 1.04 (0.80,1.36) | 2.13 (1.30,3.48) | 0.99 (0.58,1.67) |
| --- | --- | --- | --- | --- | --- | --- |
| 0.70 (0.34,1.44) | Clopidogrel+Aspirin | 1.02 (0.60,1.75) | 1.14 (0.67,1.94) | 0.73 (0.37,1.43) | 1.49 (0.87,2.54) | 0.69 (0.42,1.14) |
| 0.68 (0.39,1.19) | 0.98 (0.57,1.67) | Rivaroxaban | 1.11 (0.94,1.32) | 0.71 (0.43,1.16) | 1.45 (1.11,1.89) | 0.68 (0.56,0.82) |
| 0.61 (0.35,1.07) | 0.88 (0.52,1.49) | 0.90 (0.76,1.06) | Rivaroxaban+Aspirin | 0.64 (0.39,1.04) | 1.30 (1.01,1.68) | 0.61 (0.51,0.72) |
| 0.96 (0.74,1.25) | 1.37 (0.70,2.70) | 1.41 (0.86,2.30) | 1.57 (0.96,2.55) | Ticagrelor | 2.04 (1.35,3.09) | 0.95 (0.60,1.50) |
| 0.47 (0.29,0.77) | 0.67 (0.39,1.15) | 0.69 (0.53,0.90) | 0.77 (0.59,0.99) | 0.49 (0.32,0.74) | Ticagrelor+Aspirin | 0.46 (0.39,0.56) |
| 1.01 (0.60,1.71) | 1.45 (0.88,2.39) | 1.48 (1.23,1.79) | 1.65 (1.39,1.96) | 1.05 (0.67,1.66) | 2.15 (1.78,2.59) | Aspirin |

## eAppendix 2. Sensitivity analyses for adjustment of primary efficacy and safety outcomes by person-years in the total cohort

Odds ratio (95% credible intervals) between column and row treatment regimens are reported. Odds ratio smaller than 1 means that the odds of having an event for the column treatment regimen is lower than the row treatment regimen. Statistically significant results, where the 95% credible interval does not include 1.

1. Major adverse cardiovascular and cerebrovascular events for main analysis

| Clopidogrel | 0.84(0.49,1.44) | 1.37(0.84,2.24) | 1.18(0.73,1.91) | 1.02(0.88,1.19) | 1.31(0.84,2.07) | 1.49(0.93,2.36) |
| --- | --- | --- | --- | --- | --- | --- |
| 1.20(0.69,2.06) | Clopidogrel+Aspirin | 1.64(1.19,2.26) | 1.41(1.04,1.92) | 1.22(0.72,2.06) | 1.57(1.16,2.12) | 1.78(1.34,2.35) |
| 0.73(0.45,1.19) | 0.61(0.44,0.84) | Rivaroxaban | 0.86(0.74,1.01) | 0.74(0.47,1.19) | 0.96(0.79,1.15) | 1.08(0.93,1.26) |
| 0.85(0.52,1.37) | 0.71(0.52,0.96) | 1.16(0.99,1.36) | Rivaroxaban+Aspirin | 0.86(0.55,1.36) | 1.11(0.95,1.31) | 1.26(1.11,1.42) |
| 0.98(0.84,1.14) | 0.82(0.49,1.38) | 1.34(0.84,2.14) | 1.16(0.73,1.83) | Ticagrelor | 1.29(0.84,1.97) | 1.45(0.94,2.26) |
| 0.76(0.48,1.20) | 0.64(0.47,0.86) | 1.04(0.87,1.26) | 0.90(0.77,1.06) | 0.78(0.51,1.19) | Ticagrelor+Aspirin | 1.13(1.02,1.26) |
| 0.67(0.42,1.07) | 0.56(0.42,0.75) | 0.92(0.79,1.08) | 0.80(0.70,0.90) | 0.69(0.44,1.07) | 0.88(0.80,0.98) | Aspirin |

1. Major bleeding for main analysis

| Clopidogrel | 1.78(0.64,4.98) | 1.89(0.87,4.12) | 2.10(0.97,4.58) | 1.04(0.82,1.31) | 2.58(1.24,5.37) | 1.27(0.60,2.68) |
| --- | --- | --- | --- | --- | --- | --- |
| 0.56(0.20,1.57) | Clopidogrel+Aspirin | 1.06(0.51,2.21) | 1.18(0.57,2.46) | 0.58(0.21,1.59) | 1.45(0.70,3.00) | 0.71(0.35,1.44) |
| 0.53(0.24,1.15) | 0.94(0.45,1.96) | Rivaroxaban | 1.11(0.91,1.36) | 0.55(0.26,1.16) | 1.37(1.04,1.79) | 0.67(0.54,0.83) |
| 0.48(0.22,1.03) | 0.85(0.41,1.75) | 0.90(0.73,1.10) | Rivaroxaban+Aspirin | 0.49(0.24,1.04) | 1.23(0.95,1.59) | 0.60(0.50,0.73) |
| 0.96(0.76,1.21) | 1.71(0.63,4.66) | 1.82(0.86,3.82) | 2.02(0.96,4.24) | Ticagrelor | 2.48(1.24,4.97) | 1.22(0.60,2.48) |
| 0.39(0.19,0.81) | 0.69(0.33,1.42) | 0.73(0.56,0.96) | 0.81(0.63,1.05) | 0.40(0.20,0.81) | Ticagrelor+Aspirin | 0.49(0.41,0.58) |
| 0.79(0.37,1.67) | 1.40(0.69,2.84) | 1.49(1.21,1.84) | 1.66(1.37,2.02) | 0.82(0.40,1.68) | 2.04(1.73,2.41) | Aspirin |

1. Forest plots primary efficacy and safety outcomes for main analysis


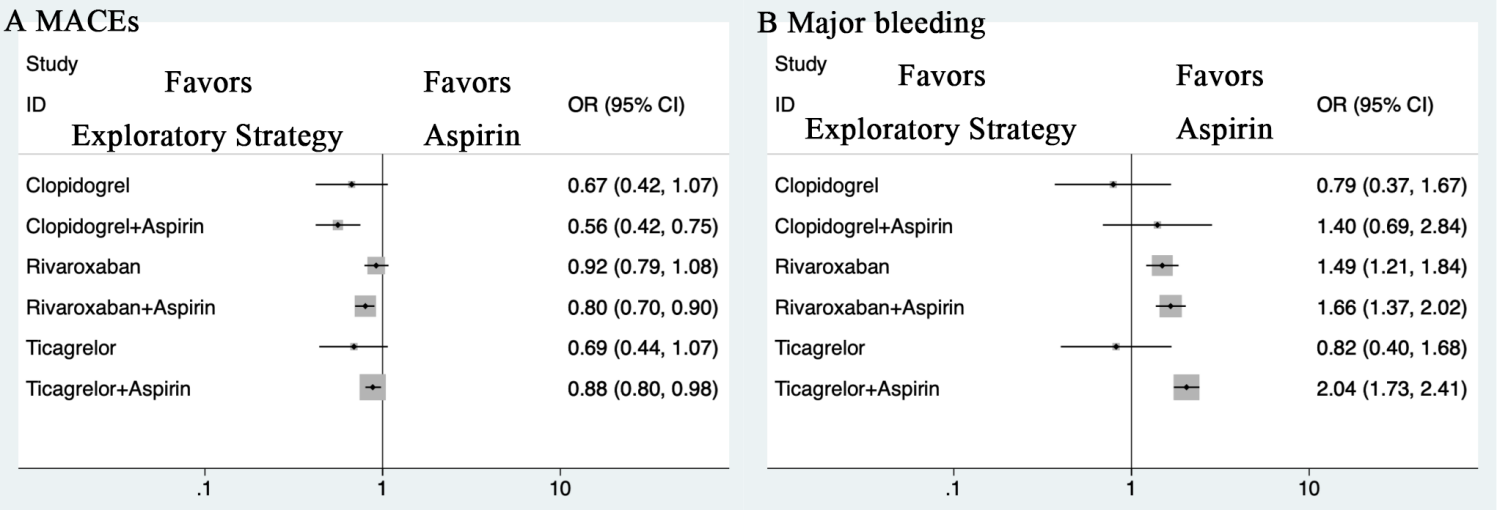


CI: credible intervals, MACEs: major adverse cardiovascular and cerebrovascular events, OR: odds ratio.
